# Effective Connectivity and Bias Entropy Improve Prediction of Dynamical Regime in Automata Networks

**DOI:** 10.3390/e25020374

**Published:** 2023-02-18

**Authors:** Felipe Xavier Costa, Jordan C. Rozum, Austin M. Marcus, Luis M. Rocha

**Affiliations:** 1 Systems Science and Industrial Engineering Department, Binghamton University (State University of New York), Binghamton, NY 13902, USA; 2 Instituto Gulbenkian de Ciência, 2780-156 Oeiras, Portugal; 3 Department of Physics, State University of New York at Albany, Albany, NY 12222, USA

**Keywords:** random Boolean networks, criticality, perturbation spreading, biomolecular networks, Derrida coefficient

## Abstract

Biomolecular network dynamics are thought to operate near the critical boundary between ordered and disordered regimes, where large perturbations to a small set of elements neither die out nor spread on average. A biomolecular automaton (e.g., gene, protein) typically has high regulatory redundancy, where small subsets of regulators determine activation via collective canalization. Previous work has shown that effective connectivity, a measure of collective canalization, leads to improved dynamical regime prediction for homogeneous automata networks. We expand this by (i) studying random Boolean networks (RBNs) with heterogeneous in-degree distributions, (ii) considering additional experimentally validated automata network models of biomolecular processes, and (iii) considering new measures of heterogeneity in automata network logic. We found that effective connectivity improves dynamical regime prediction in the models considered; in RBNs, combining effective connectivity with bias entropy further improves the prediction. Our work yields a new understanding of criticality in biomolecular networks that accounts for collective canalization, redundancy, and heterogeneity in the connectivity and logic of their automata models. The strong link we demonstrate between criticality and regulatory redundancy provides a means to modulate the dynamical regime of biochemical networks.

## 1. Introduction

The collective behavior of coupled automata is governed by the interplay between structural and dynamical parameters [1,2,3,4,5,6]. Tuning a small number of these parameters can lead to dramatic changes in the emergent properties of interlinked automata. A foundational example that illustrates this is the random Boolean network (RBN) models of gene regulation introduced by Kauffman [7], which have sustained interest over the intervening five decades (reviewed in [8,9]). In the Kauffman model, each of *N* Boolean automata (nodes) receives inputs from exactly *K* other nodes, chosen uniformly at random. An update function for each node is randomly generated by independently and randomly assigning an output value to each of the $2^{K}$ possible input configurations, such that the output is 1 with probability *P*. The probability of activation of each input, *P*, is shared among all nodes in a network and is known as bias.

At each time-step, the vector of node variable values, called the network configuration, is synchronously updated according to these update functions.

The response of RBNs to perturbations has been of particular interest and is traditionally measured by the Derrida coefficient, δ. This parameter is defined as the separation (Hamming distance) after one time-step between two network configurations that initially differ in only one node value [10,11]. In the thermodynamic limit, N→∞, RBNs undergo an order to chaos phase transition characterized by the critical boundary δ=1. In the ordered regime, when δ is below this threshold, trajectories are characterized, on average, by short transient lengths and quickly vanishing perturbations. In the chaotic regime, when δ is above this threshold, transient lengths are long and perturbations grow in time, on average. Along the critical boundary, δ=1, on average, perturbations neither grow nor decay.

Contributions to the Derrida coefficient from an individual automaton can be measured using its sensitivity, which is defined as the number of inputs that can individually toggle the output of the automaton, averaged over all possible input configurations [12]. The average sensitivity of the automata in a Boolean network gives the Derrida coefficient. In the thermodynamic limit, sensitivity can be computed as 2KP(1−P), which gives rise to the classical critical boundary [10,11]:

(1) 2KP(1−P)=1.

A particularly relevant interpretation of Equation (1) is that it decomposes the Derrida coefficient into two contributions: average in-degree (*K*), which describes the average number of inputs nodes have, and bias-variance (P(1−P)), which describes how much spread there is in the distribution of activation probability (for all automata nodes in the network or ensemble.) The infinite-size limit in which the thermodynamic theory applies is an idealization, nevertheless, characteristics of the order to chaos transition can be observed in networks of eukaryotic cells [13], gene transcription [14], and other empirical databases [15,16] that have many fewer nodes than the typical number of protein-coding genes in an organism.

Various extensions of the Kauffman model have been studied to examine features of biomolecular networks that are not emphasized in the traditional model. For instance, gene regulatory networks tend to exhibit high modularity and power-law degree distributions. As such, modifications to the network structure of the Kauffman model have been considered for any in-degree distribution [17], power-law in-degree structure [8,18], and others [19]. Furthermore, in the Kauffman model, all update functions with the same activation bias are equally likely, but the regulatory logic of real biological networks is known to have a highly non-random structure [20]. To account for this, random Boolean models that use alternate methods for generating update functions, such as nested canalizing Boolean functions [21,22] and random threshold networks [23,24], have been proposed.

Here, we take structural heterogeneity into account directly by constructing RBNs with a truncated power-law in-degree distribution. Additionally, we consider the dynamical impact of regulatory logic through the lens of collective canalization. Broadly, the term canalization, coined by Waddington [25], refers to the ability of a small subset of variables (sometimes just a single variable) to determine the outcome of a regulatory process. Various measures have been proposed to quantify this behavior [26,27,28,29]. These measures are not necessarily in agreement about which Boolean functions are more or less canalizing than others. It is generally agreed, however, that the concept of canalization is closely related to robustness to genetic perturbations, which has been shown to play a crucial role in the ensemble properties of RBNs [7,12].

Collective canalization [20,26,28,30] refers to the degree to which a small subset of jointly activated inputs renders other inputs redundant. Effective connectivity, $k_{e}$, has been proposed to measure this effect by computing the average size of the subset of inputs necessary to determine the output of an automaton [20,28]. It is obtained by computing the set of all prime implicants of a Boolean function (or the automaton’s look-up-table), which yields a maximal set of irreducible conditions for dynamical transition (see Appendix A for formal definition). This is equivalent to identifying and removing dynamical redundancy [28]. In other words, effective connectivity is the dual concept of dynamical redundancy in the logic of (collectively) canalized automata transitions. Bounded from above by in-degree, *k*, $k_{e}$ attains this maximum only when every input state must be known to determine the automaton’s next logical state. This only occurs for the parity functions (such as the case of a non-constant function of one variable or the XOR function of two variables). These are situations without any logical redundancy (or collective canalization). In the case of tautologies or contradictions (i.e., constant Boolean functions), $k_{e}=0$ by definition, which denotes that all inputs are fully redundant.

Removing dynamical redundancy has already been used to reveal an alternative dynamically effective structure that includes collective canalization effects and is useful to characterize control in biochemical signaling and regulatory pathways [4,20]. Certainly, network controllability is an important aspect of automata models of biochemical regulation [31,32]. It is equally important to understand how perturbations spread in such models. Therefore, we focus here on the relevance of effective connectivity in determining the dynamical regime of Boolean networks and characterizing the critical boundary between order and chaos. Revising Equation (1) to utilize effective connectivity ($k_{e}$) instead of in-degree (*k*), previous work has shown a significant improvement in dynamical regime prediction (as chaotic, critical, or ordered) of finite-size RBNs with homogeneous in-degree [33]. In other words, collective canalization (as measured by effective connectivity) explains the dynamical regime better than the apparent (structural) connectivity of such networks.

Here, we build upon that work to study RBNs with power-law in-degree distributions and study a larger set of experimentally validated Boolean network models of biomolecular processes. We show that in finite random networks and experimentally validated models, effective connectivity and bias-variance provide a better prediction of the dynamical regime—as measured by the Derrida coefficient and sensitivity—than the classical boundary of Equation (1) defined by the in-degree and bias-variance in the thermodynamic limit. We also show that the prediction of the Derrida coefficient is further improved in random networks by measuring the spread in bias using the entropy instead of the variance. In empirical models, the difference between the entropy and the bias is less pronounced, and the two measures perform similarly in predicting the dynamical regime.

## 2. Materials and Methods

### 2.1. Boolean Network Models

A Boolean automaton, $x_{i}$, is represented by a time-dependent binary variable $x_{i}^{t}\in \left\{0,1\right\}$ whose state at a subsequent time-step is determined by an update function $x_{i}^{t+1}=f_{i}\left(x_{j}^{t},\ldots ,x_{l}^{t}\right)$ with time-dependent binary arguments. Combining multiple automata, we construct a Boolean network, which is a directed graph G=(X,E) with nodes $x_{i}\in X$ corresponding to the N=|X| Boolean automata, while the edges $(x_{j},x_{i})\in E$ denotes that $x_{j}^{t}$ is an argument to the update function $f_{i}$. Following the most common convention in the study of RBNs, we consider automata that update synchronously. The number of automata considered for the update of $x_{i}$ (i.e., the number of arguments in $f_{i}$) is called the in-degree of the node $x_{i}$ and is denoted $k_{i}$.

We study two different ensembles of Boolean networks: RBNs with truncated power-law in-degree distributions, and empirical network models from the Cell Collective [34].

The RBNs are generated with an in-degree distribution given by

(2) $$P_{in}\left(k_{i}\right)=\left\{\begin{array}{cc} \frac{k_{i}^{-\gamma }}{\sum_{\kappa =1}^{k_{max}}\kappa^{-\gamma }} & ,\;1\leq k_{i}\leq k_{max}\\ 0 & ,\;\mathrm{otherwise} \end{array}\right..$$

In this study, we set a cut-off $k_{max}=15$, a value that is inspired by the maximum in-degree of real models from the Cell Collective. In the thermodynamic limit (N→∞), the cut-off can be set to infinity and an exact formula for $K=\langle k_{i}\rangle$ in terms of γ is found [8]. However, finite-size effects are an intrinsic feature of empirical Boolean network models. To investigate these effects, we consider networks of sizes N=20,50,100, and 200. We sweep the P−γ parameter space using P∈{0.05,0.10,…0.45} and γ∈{1.5,1.6,…,2.4}. For each combination of *N*, *P*, and γ, we generate 400 networks.

The Cell Collective is a collection of experimentally validated Boolean networks modeling various cellular processes, created by aggregating detailed empirical knowledge of cellular mechanisms. Each edge in the Cell Collective models is associated with experimental results from the literature. This study uses these models to explore how RBN-based arguments on criticality apply to empirical models of biology. We analyze 74 of the Cell Collective models, including various networks related to cancer, and the immune system, among others, in humans and other organisms.

### 2.2. Characterizing the Critical Boundary

The Derrida Coefficient, δ [10,11], is a measure of trajectory divergence in response to perturbations, commonly used to estimate the degree to which a system is chaotic [17,33,35,36]. We calculate δ in the generated RBNs and the Cell Collective models via the Hamming distance between trajectories of the network. Specifically, for each network, we estimate the Derrida coefficient, δ, using a sample of 1000 initial states for each randomly generated network and 8000 samples for each network from the Cell Collective. We apply a single variable perturbation to each initial state and compute the Hamming distance between the perturbed and unperturbed states after one time-step, averaging over all initial states. A value of δ<1 corresponds to the ordered regime, while δ>1 corresponds to the chaotic regime. Therefore, δ=1 gives the critical boundary.

We fit the Derrida coefficient δ to the structural and dynamical properties of RBNs and Cell Collective models. We consider connectivity, effective connectivity, bias-variance, and bias entropy. The dynamical simulations and these network measures were performed using the Python package CANA [37].

Effective connectivity, defined in [20,28], generalizes in-degree to account for redundancy present in Boolean functions; essentially, it is the extent to which subsets of input variables collectively determine the output of a Boolean automaton. It was found by [33] that $k_{e}$, the average effective connectivity of a network, predicts criticality better than the average in-degree in an ensemble of homogeneous Kauffman-like networks, and we consider it here for heterogeneous RBNs and Cell Collective network models. See Appendix A for more detail on effective connectivity.

The average bias, *p*, of a Boolean network is computed as the average bias of the automata in the network: $p=\frac{1}{N}\sum_{i}^{N}p_{i}$, where $p_{i}$ is the proportion of input configurations to the update function of node *i* that result in an output of 1. The bias entropy *H* of a Boolean network is the Shannon entropy of a Bernoulli random variable whose success probability is the average bias *p*. That is, *H* is given by

(3) $$H=-p\log_{2}p-\left(1-p\right)\log_{2}\left(1-p\right).$$

Interpreting *p* in this way leads to a similar definition for bias variance as

(4) $$\sigma^{2}=p\left(1-p\right).$$

For the Cell Collective models, we also consider an additional averaging scheme to compute the entropy and variance of a network, in which the bias entropy and variance are calculated separately for each node and then averaged. We call these the average node entropy and average node variance, denoted by $H^{\prime }$ and ${\left(\sigma^{2}\right)}^{\prime }$, respectively, to distinguish them from the network entropy and variance, which are computed from the average node bias *p*. The average node entropy can be calculated as

(5) $$H^{\prime }=\frac{1}{N}\sum_{i}^{N}\left[-p_{i}\log_{2}p_{i}-\left(1-p_{i}\right)\log_{2}\left(1-p_{i}\right)\right],$$

and the average node variance can be computed as

(6) $${\left(\sigma^{2}\right)}^{\prime }=\frac{1}{N}\sum_{i}^{N}p_{i}\left(1-p_{i}\right).$$

Note that because the networks considered here are finite, the average bias *p* and average network degree *k* for a given sampled network may differ from the population averages *P* and *K*, respectively.

The computational complexity of computing effective connectivity for a network is dominated by the Quine–McCluskey algorithm for computing the prime implicants, which scales exponentially with *k*, and linearly with *N*. However, we consider a maximum *k* of 15, which is tractable; computation becomes difficult around k=25. Bias entropy is dominated by the complexity of computing the bias of a Boolean function: the same complexity as the traditional measure of bias-variance. In our implementation, this is exponential in *k* and linear in *N*. Therefore, the complexity of our methods overall is exponential in *k* and linear in *N*: the same as the traditional methods.

In Section 3, we illustrate that the relationship between these various connectivity and bias spread parameters can predict the dynamical regime of a network.

## 3. Results

### 3.1. Critical Boundaries in Finite Heterogeneous Random Networks

**Figure 1:**
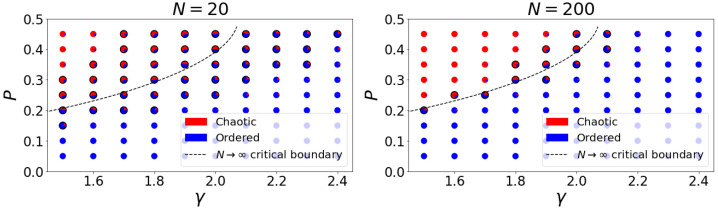
Proportion of chaotic (red) and ordered (blue) networks from the 400 samples at each point in the P−γ parameter space for two values of *N*. Plots for N=50 and N=100 are provided in the Supplemental Notebook (see Figure S1). The dashed curve is the critical boundary in the thermodynamic limit, N→∞ [8]. Black borders are added to the points for which between 15% and 85% of networks are chaotic (or, equivalently, ordered). These points form a critical region that shrinks as *N* increases, apparently converging to the thermodynamic critical boundary.

In the thermodynamic limit, N→∞, the critical boundary 2KP(1−P)=1 separating order and chaos becomes infinitely sharp. In finite networks, however, the “critical” regime becomes less clear-cut: the boundary is blurred and smudged. Finite networks for which 2KP(1−P)>1 holds may quickly extinguish perturbations, and networks for which 2KP(1−P)<1 may exhibit high sensitivity to perturbations. In Figure 1, we depict, for N=20 and N=200, the proportion of the random networks we have generated that show perturbation growth (Derrida coefficient greater than one) or decay (Derrida coefficient less than one) for each P−γ pair we sampled. We highlight a “critical region”, in which sampled networks exhibit both chaotic and ordered behaviors (i.e., between 15% and 85% of sampled networks have Derrida coefficients greater than one). As the network size increases, the critical region shrinks, converging toward the thermodynamic boundary, as guaranteed by [8] (see Supplemental Figure S1 for N=50 and N=100 plots).

As Figure 1 depicts, it is not straightforward to use thermodynamic boundaries to separate finite networks into dynamical regimes using structural and bias parameters alone. In the results that follow, we consider alternative parameters that sharpen the boundary between order and chaos by incorporating canalization into the characterization of the network structure. In particular, we consider dynamical boundaries determined by the effective connectivity, $k_{e}$ which may be thought of as an effective in-degree. Because $k_{e}$ differs from *k*, it is not clear that bias variance is the appropriate measure of spread in the node outputs, though earlier work [33] suggests that it is the best polynomial measure of spread for homogeneous RBNs. We therefore consider both the variance of the sampled bias Equation (4), and its entropy Equation (3) as competing measures of spread to be paired with effectiveness.

**Figure 2:**
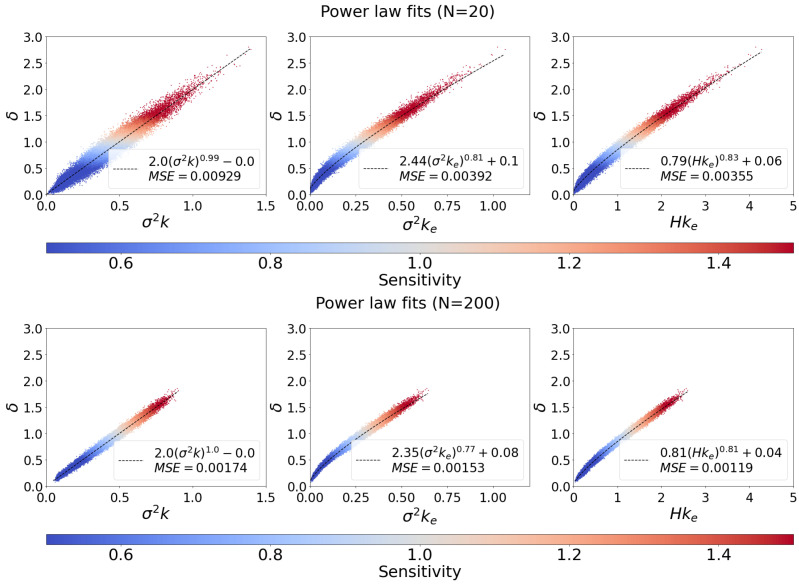
Ability of each measure to predict the Derrida coefficient of heterogeneous random networks. Each point corresponds to a sampled network, and its color indicates the network’s sensitivity. The curves are obtained by fitting a power-law function of various measures to the Derrida coefficient, δ: kp(1−p) to the δ (left), the traditional comparison, $k_{e}p\left(1-p\right)$ to δ (center), and $Hk_{e}$ to δ (right), our proposed measures. Plots for two sizes of networks (N=20, top; N=200, bottom) are presented here. Plots for N=50 and N=100 are available in the Supplemental Figure S3. Plots combine networks sampled from all considered *P* and γ parameter values; thus each plot depicts 36,000 networks. An alternate curve fit is also investigated in the Supplemental Figure S2. All additional plots show qualitatively similar results to those captured in the images presented in this figure.

We consider the extent to which disorder, as measured by the Derrida coefficient δ, can be predicted by structural measures (*k* or $k_{e}$) and measures of bias spread ($\sigma^{2}$ or *H*) by fitting power-law functions to the distributions of points in the $\sigma^{2}k-\delta$, $\sigma^{2}k_{e}-\delta$, and $Hk_{e}-\delta$ parameters spaces for various values of *N* Figure 2. Notably, the only significant nonlinearity in these plots occurs for low values of δ. If these low values are excluded, e.g., to focus more closely on the critical regime, a linear fit is sufficient, which presents similar qualitative features (see Supplemental Figure S3). Nevertheless, the power-law dependence we encountered is not far from linear, having exponents ranging from 0.77 to 1.0. Generally, the ability of $\sigma^{2}k$, $\sigma^{2}k_{e}$, and $Hk_{e}$ to predict δ improves as *N* increases (compare the two rows of panels in Figure 2; see Supplemental Figure S2 for additional values of *N*). The $\sigma^{2}k_{e}$ fit to δ consistently provides a more accurate estimate of δ than the $\sigma^{2}k$ fit, and the performance of the $Hk_{e}$ fit is better than either of them. This pattern persists across all network sizes considered, and for both the power-law fits and the restricted linear fits.

**Figure 3:**
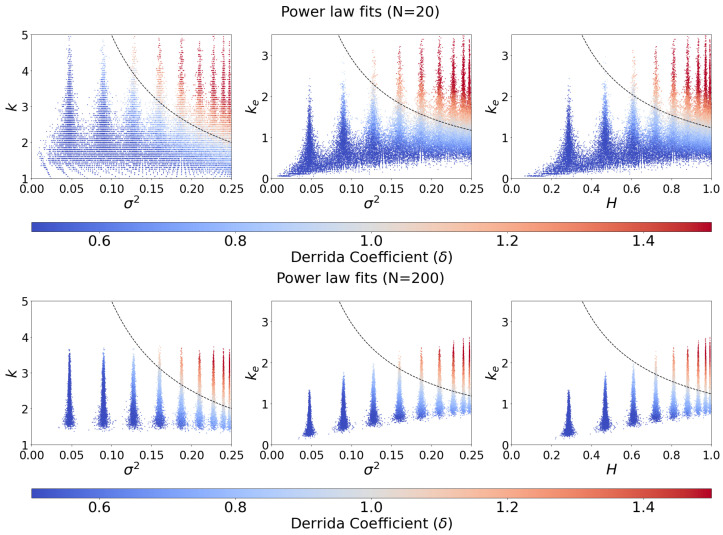
Critical boundaries were obtained from fitting connectivity-spread products to the Derrida coefficient. The color of each node indicates the network’s Derrida coefficient. The critical boundary (dashed curve) is obtained by setting the power-law fit found in Figure 2 to 1 and inverting for *k* (left), what is traditionally considered, or $k_{e}$ (center and right), our proposed measures. Plots for N=50 and N=100 are provided in the Supplemental Figure S4.

The critical boundary obtained from the power-law fits is provided in Figure 3. These boundaries are found by setting the fitted power-law functions to one and inverting for the connectivity parameter (either *k* or $k_{e}$). Note that the finite number of nodes in the RBN leads to a spread in the sample bias, *p*, shown as the spread in $\sigma^{2}$ and *H*, centered at the population bias parameter *P*. This effect becomes less pronounced as *N* increases.

In all cases, the critical boundaries obtained from the power-law fits accurately predict the dynamical regime (between 93.8% and 97.5% accuracy; see Supplemental Figure S5). This is reflected in Figure 3 as the sharpness of the separation between ordered (blue) and chaotic (red) points provided by the dashed curves (fit critical boundary).

### 3.2. Estimating the Critical Boundary for Empirical Models

As reviewed in the introduction, empirical Boolean models of biomolecular processes differ in significant ways from random ensembles. Thus, it is not always clear which results derived for the latter are extensible to the domain of the former. In this section, we examine this question using the Cell Collective [34] as a case study.

In empirical models, the measures considered in Figure 2 and Figure 3 do not align as neatly with the Derrida coefficient as they did in the randomly generated networks (see Supplemental Figures S7 and S9), and accordingly, caution is required when attempting to fit the critical boundary. Thus, when considering $\sigma^{2}$ and *H* for those models, we optimize the binary classification of the dynamical regime instead of the mean squared error of a curve-fit. We consider three metrics to select the optimal boundary for discriminating between the ordered and chaotic regimes: the Matthews correlation coefficient (MCC) [38,39], the accuracy, and the Cohen kappa. There are more ordered models in the Cell Collective (46) than chaotic (28). Because they are in the minority, chaotic models are assigned the positive label, but all three metrics are insensitive to this choice. The MCC and Cohen kappa metrics more harshly penalize differences between the false positive and false negative rates than the accuracy does, making them better-suited to situations in which the class frequency is imbalanced. In this case, the imbalance is appreciable, but not extreme, so the accuracy is also meaningful. The performance of the thresholds for the connectivity spread products are summarized as confusion matrices provided in the Supplemental Figure S8, and illustrate that the $\sigma^{2}k_{e}$ and $Hk_{e}$ measures perform similarly to one another and much better than the $\sigma^{2}k$ measure. The critical boundaries estimated from the N=50 RBNs (which are closest in size to the average size of the Cell Collective networks) show good agreement with the most accurate classification boundaries for the Cell Collective in the cases where $k_{e}$ is used, though the boundaries are more widely separated when *k* is considered (see Supplemental Figure S9).

The theory of RBNs considers $\sigma^{2}$ as computed from the overall bias of the network, rather than computed from the average of each node’s output variance. This traditional approach gives rise to the classical results in the theory. However, the second, less-conventional approach we propose here, Equations (5) and (6), has dramatically better performance in the Cell Collective than the traditional averaging approach (especially when paired with effective connectivity). In the remainder of this section, we use the prime to denote that the parameters are computed using this alternate averaging scheme.

**Figure 4:**
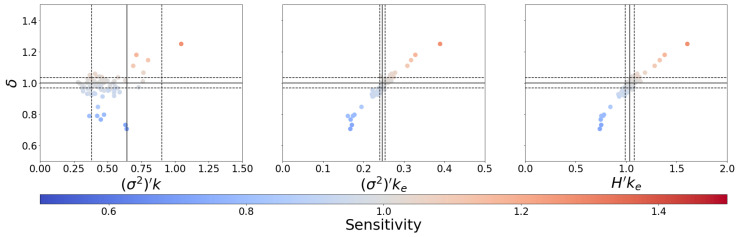
Relationship between the Derrida coefficient and connectivity-spread products for Cell Collective [34] models using an alternate averaging scheme. The leftmost panel represents the traditional in-degree approach, while the central and rightmost ones consider our proposed measures. The activation spread parameters ${\left(\sigma^{2}\right)}^{\prime }$ and $H^{\prime }$ are computed by averaging node activation variance and entropy, respectively. The color of each point indicates the network’s sensitivity. The region between the dotted horizontal lines indicates a critical region centered at δ=1(solid horizontal line) of width equal to the IQR of the δ distribution (0.06). Each vertical line corresponds to an optimal binary (solid) or ternary (dotted) classification threshold as described in the text.

**Figure 5:**
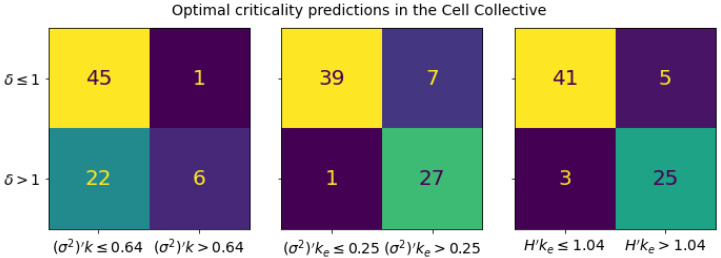
Confusion matrices for the optimal critical boundaries in the Cell Collective computed using average node activation spread measures. Each boundary was optimized to maximize the Matthews correlation coefficient (MCC), the accuracy, and the Cohen kappa metric. Each matrix corresponds to a given threshold parameter that is evaluated to predict the dynamical regime. From left to right, these are ${\left(\sigma^{2}\right)}^{\prime }k$, the traditional connectivity-spread product, ${\left(\sigma^{2}\right)}^{\prime }k_{e}$, and $H^{\prime }k_{e}$, our proposed measures. The predicted regime is given by the horizontal labels, and the ground truth regime, as computed from the Derrida coefficient, is given by the vertical labels. In all cases, the obtained boundary simultaneously maximized all three performance metrics (see Supplemental Figure S11). From left to right, the MCCs are 0.32, 0.79, 0.77; the accuracies are 0.69, 0.89, 0.89; and the Cohen kappa scores are 0.23, 0.78, 0.77.

**Figure 6:**
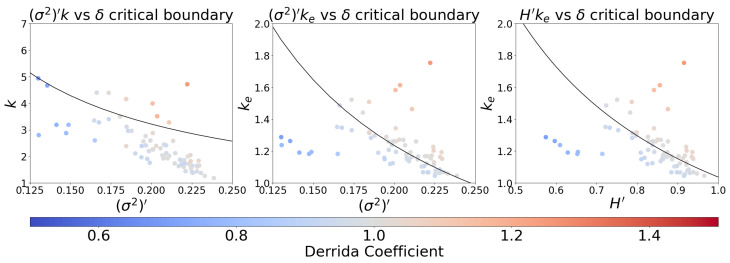
Estimated critical boundaries for an ensemble of empirical models, using an alternative averaging scheme. The leftmost panel represents the traditional in-degree approach, while the central and rightmost ones consider our proposed measures. The activation spread parameters ${\left(\sigma^{2}\right)}^{\prime }$ and $H^{\prime }$ are computed by averaging node activation variance and entropy, respectively. The color of each point indicates the network’s Derrida coefficient. The curves are estimates of the critical boundary obtained from the optimal boundary in the Cell Collective data, which simultaneously maximizes the MCC, accuracy, and Cohen kappa metric in all three cases.

We produce the distribution of network parameters using this alternate averaging scheme in Figure 4. The correlation between δ and the new measures in Figure 4 is dramatically improved relative to the traditionally averaged measures in all cases (cf. Supplemental Figures S10 through S13). Figure 5 demonstrates the performance of an optimal criticality boundary obtained for each connectivity-spread measure, which can be seen as solid lines in Figure 4. For all three measures, the same boundary simultaneously optimizes the MCC, accuracy, and Cohen kappa metrics (see Supplemental Figure S11). The optimal critical boundaries are plotted in Figure 6.

**Figure 7:**
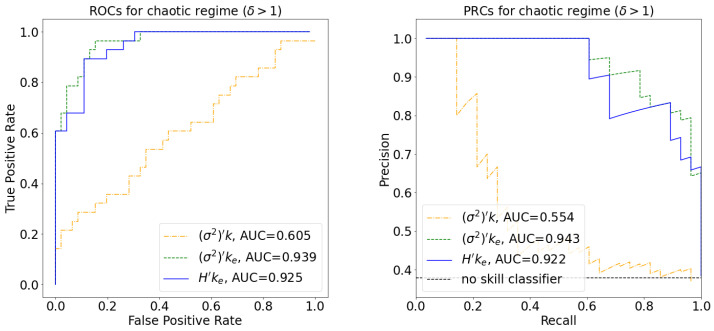
Receiver operating characteristic (**left**) and precision recall curve (**right**) for the classifiers in Figure 5. Empirical networks with δ>1 are considered positives. Performance is measured on automata networks from Cell Collective, and the areas under the curve are depicted in the legend. A random classifier has AUROC=0.5 and AUPRC≈0.38.

Though the boundaries we have obtained are optimal according to the metrics considered, we also explored their dependence on the identified thresholds by constructing the receiver operating characteristic (ROC) and precision recall curve (PRC) for each classifier, depicted in Figure 7. The performance of the two classifiers that use $k_{e}$ is similar and significantly better than the measure that uses *k* as the connectivity parameter.

Figure 5 and Figure 7 illustrate the performance of ${\left(\sigma^{2}\right)}^{\prime }k$, ${\left(\sigma^{2}\right)}^{\prime }k_{e}$, and $H^{\prime }k_{e}$ in predicting the dynamical regime as measured by the Derrida coefficient. Analogous figures provided in the Supplemental Notebook (see Figures S18 through S20) demonstrate that these products can achieve up to 20% better performance (except for Cohen kappa involving *k*, with a 35% increase) when the dynamical regime is determined by sensitivity, rather than the Derrida coefficient. These similarly illustrate significantly better performance for ${\left(\sigma^{2}\right)}^{\prime }k_{e}$ and $H^{\prime }k_{e}$ boundaries than for ${\left(\sigma^{2}\right)}^{\prime }k$.

**Figure 8:**
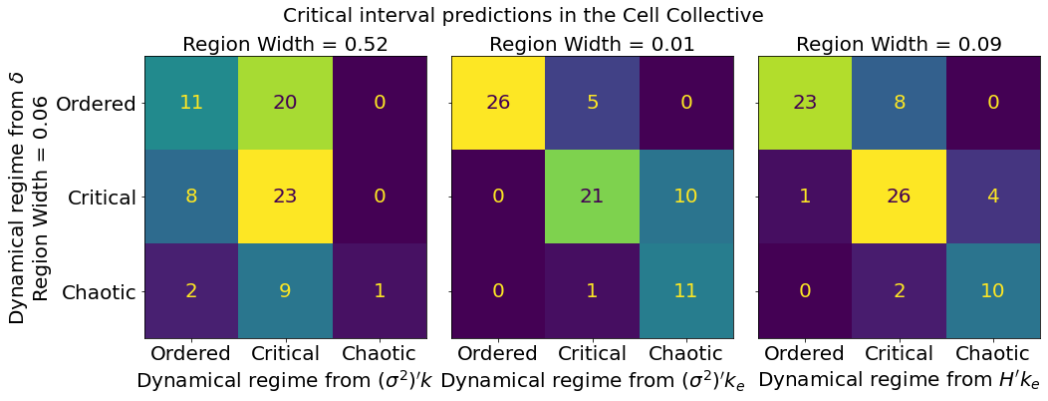
Confusion matrices for various critical boundaries in the Cell Collective computed using average node activation spread measures. Each matrix corresponds to a given threshold parameter that is evaluated to predict the dynamical regime. From left to right, these are ${\left(\sigma^{2}\right)}^{\prime }k$, as in the traditional in-degree approach, followed by ${\left(\sigma^{2}\right)}^{\prime }k_{e}$, and $H^{\prime }k_{e}$, our proposed measures. The predicted regime is given by the horizontal labels, and the ground truth regime, as computed from the Derrida coefficient, is given by the vertical labels. The center of each predicted critical regime is taken to be the corresponding binary classification boundary in Figure 5, and the width of the predicted critical regime is chosen to maximize the accuracy of each classifier. The ground truth critical regime is defined as the range of Derrida coefficient values centered at δ=1 with a width equal to the IQR of the Derrida coefficient distribution in the Cell Collective.

To examine the ability of these measures to identify a critical regime, rather than only the separation between regimes, we take the interquartile range (IQR) of the Derrida coefficient distribution to define the width of a critical regime centered on δ=1. From this interval, we construct three classes: critical (δ in this interval), ordered (δ below this interval), and chaotic (δ above this interval). We maximize the classification accuracy for each connectivity-spread product in terms of the critical interval’s width, centered on the optimal separation obtained from Figure 5. Those intervals can be observed as dashed lines in Figure 4. The confusion matrices for these classes (Figure 8) again show that the classifiers using $k_{e}$ outperform the ones using *k* alone.

## 4. Discussion

Theories about the dynamical regime of Boolean networks were originally considered in the thermodynamic limit (N→∞) in random homogeneous networks [7,10]. However, degree heterogeneity [8,17] and finite-size effects are important, especially in experimentally-validated models of biochemical regulation [13,14,16]. Such systems have update rules (for each Boolean automaton) that are highly canalizing, meaning that there is a tendency for combinations of some inputs to render other inputs redundant. We have shown that the amount of this type of redundancy is highly predictive of the dynamical regime of Boolean networks. In ensembles of both experimentally-validated automata models and heterogeneous RBNs, we have shown that a measure of collective canalization, the network average effective connectivity $k_{e}$, is a more accurate predictor of the network’s dynamical regime (as determined by the Derrida coefficient and sensitivity) than the widely-used average in-degree *k* of a network (see Section 3). Furthermore, the prediction of perturbation response using $k_{e}$ remains more accurate far from the critical boundary: The mean squared error between the Derrida coefficient δ and a power-law fit to $\sigma^{2}k_{e}$ is smaller than that for $\sigma^{2}k$ in random networks (see Figure 2).

We have also shown that measuring bias spread via its entropy *H* (Equation (3)) rather than variance $\sigma^{2}$ (Equation (4)) improves the prediction of the dynamical regime. Combined with $k_{e}$, *H* provides the best fit (and classification performance) against the Derrida coefficient observed for finite heterogeneous RBNs. Over a broad range of the bias values *p* considered, *H* and $\sigma^{2}$ are approximately linearly related, but this relationship is weaker at more extreme values of $\sigma^{2}$, and the departure is particularly pronounced when *p* is near zero. Near this low-bias regime, the distribution of RBNs in the δ-$\sigma^{2}k_{e}$ plane becomes kinked (see Figure 2). Considering *H* in place of $\sigma^{2}$ appears to partially compensate for this kink and results in a more accurate fit to the Derrida coefficient (an improvement of more than 20% in mean square error for the N=200 case). This suggests that *H* and $k_{e}$ contain complementary information about perturbation response in the ordered regime that is not captured by $\sigma^{2}$ and *k*.

In predicting the dynamical regime of the experimentally-validated automata models in the Cell Collective, the difference in performance between *k* and $k_{e}$ is dramatic: $k_{e}$ provides a much better estimate of the dynamical regime as measured by both the Derrida coefficient and sensitivity (see Section 3). This suggests that collective canalization (and its dual, redundancy) is an important factor in the dynamics of biochemical regulation and signaling. Interestingly, we also found that the dynamical regime is better predicted (by any measure) in the Cell Collective models if we compute the spread in the bias using the average node output variance ${\left(\sigma^{2}\right)}^{\prime }$(Equation(6)) and average node output entropy $H^{\prime }$(Equation (5)), instead of the variance entropy of the average node bias, $\sigma^{2}$ and *H* (see Section 3 for results). Unlike for the RBNs, in the experimentally-validated models, $\sigma^{2}k_{e}$ and ${\left(\sigma^{2}\right)}^{\prime }k_{e}$ yield similar or slightly improved performance when compared to $Hk_{e}$ or $H^{\prime }k_{e}$ overall. This is unsurprising because the primary difference between these measures observed for the RBNs occurs for Derrida coefficients near 0, but the smallest Derrida coefficient value found in the Cell Collective is ≈0.7.

We determined the dynamical regime by computing the Derrida coefficient δ using a synchronous update scheme for a single time-step, following the convention of the literature [33,35,36]. The synchronous update scheme is analytically and computationally simple and is assumed in the construction and validation of many of the models in the Cell Collective. Computing the Derrida coefficient in this way allows for straightforward comparison with prior results in the theory of RBNs and the study of empirical models. However, various extensions to this approach are possible. For example, one may consider additional time-steps to measure the deviation captured by δ. A single time-step may not be long enough to fully characterize the dynamical regime of the network: Trajectories that initially diverge may converge after additional time-steps, especially in networks with highly canalized functions. It is also possible to consider modifications to the updating scheme. The synchronous update we use in the computation of the Derrida coefficient offers a deterministic baseline for future comparisons to asynchronous schemes that may introduce stochasticity into the update schedule. Such schemes attempt to account for the fact that the various biomolecular processes in a cell are not executed simultaneously. Researchers have studied alternative update schemes in RBNs [40,41], and shown that the long-term behaviors of the network depend strongly on the updating scheme [42,43].

Such concerns are partially addressed in the present work by also considering the sensitivity measure, which is ostensibly an update-independent measure of criticality. Unfortunately, the relationship between sensitivity and the dynamical regime has always been studied by way of the Derrida coefficient itself, so the extent to which the Derrida coefficient and the sensitivity parameter are independent measures of the dynamical regime in alternative updating schemes is unclear. To attempt to step out of this circularity, in future work we will consider more direct measures of the dynamical regime (e.g., transient length and long-term robustness to perturbations), and distinct update schemes. Indeed, the effective connectivity parameter of Boolean automata (together with average bias entropy) provides a new perspective on criticality that is complementary to the Derrida coefficient and sensitivity. This new method measures the expected output of an automaton from perturbations to subsets of inputs, not just individual ones. In other words, it measures how automata are collectively canalized. Unlike sensitivity, it does not assume input independence, which hitherto has underpinned the traditional framework of the study of criticality via Boolean networks. Therefore, to fully study the role of collective canalization in predicting criticality, future work should use measures of the dynamical regime that supplement the assumptions of sensitivity and the Derrida coefficient.

We have provided experimental evidence that the transition from order to chaos in realistic (finite) automata networks with a corresponding critical boundary region is more accurately characterized by measuring collective canalization (removing logical redundancy). We obtain additional improvements by accounting for bias via the network’s entropy. This more characteristic decomposition of the dynamical regime suggests that redundancy and collective effects of inputs, whereby subsets of inputs jointly control automata dynamics, is an important factor in biochemical regulation and signaling dynamics. Indeed, our results reveal that realistic networks predicted to be chaotic when considering only their connectivity and bias at the thermodynamic limit, can exist in critical and even stable regimes. The prevalence of critical behavior in biological systems is believed to be due to their efficiency in task performance and resilience to environmental constraints [15]. The existence of much dynamical redundancy in random and experimentally-validated networks [33,44], and the more accurate prediction of dynamical regime shown here when collective canalization is accounted for, suggests that biological systems use interaction redundancy to obtain an underlying effective structure that buffers excessive dynamical propagation of perturbations while maintaining high connectivity.

## Data Availability

This work analyzed models in the Cell Collective dataset available publicly at https://cellcollective.org (accessed on 12 March 2021) or https://github.com/rionbr/CANA/tree/master/cana/datasets/cell_collective (acessed on 10 February 2023). This study also generated data for analysis, which is available at https://github.com/fcphysics/CriticalityHeteroBNets (accessed on 10 February 2023).

## Supplementary Materials

The following supporting information can be downloaded at: https://www.mdpi.com/article/10.3390/e25020374/s1; The Supplemental Notebook is available in PDF format as a supplemental file, and an interactive version can be downloaded at: https://github.com/fcphysics/CriticalityHeteroBNets/blob/main/results_for_manuscript.ipynb (accessed on 10 February 2023).

## Abbreviations

The following abbreviations are used in this manuscript:

RBN	Random Boolean Network
MCC	Matthews correlation coefficient
ROC	Receiver operating characteristic
PRC	Precision recall curve
AUC	Area under curve
AUROC	Area under receiver operating characteristic
AUPRC	Area under precision recall curve
IQR	Interquartile range

## Appendix A. Formal Definition of *k_e_*

The effective connectivity, $k_{e}$, defined in [28], is a measure of the in-degree of a Boolean function that accounts for redundancy in the function. In this setting, the redundancy of a Boolean function is determined by the structure of its prime implicants and of its negation’s prime implicants. In each input configuration, the average size (number of literals) of the prime implicants of the function (or its negation) that are consistent with the input configuration is computed. This average prime implicant size is then averaged across all input configurations to obtain effective connectivity. Somewhat informally, this describes the average number of variables required to determine the output of the function.

More precisely, the effective connectivity of a Boolean function, *f* is the average size of the prime implicants that cover a given input configuration, averaged over all input configurations:

$$k_{e}\left(f\right)=\underset{x\in B^{k}}{\mathrm{avg}}\;\underset{i\in I_{f}\left(x\right)}{\mathrm{avg}}l\left(i\right)$$

where B={0,1}, *k* is the number of inputs to the Boolean function *f*, $I_{f}\left(x\right)$ is the set of prime implicants of *f* and of its negation that covers input configuration *x*, and l(i) is the number of Boolean literals (variables) in prime implicant *i*.

As an example, consider the simple AND function $f\left(x_{1},x_{2}\right)=x_{1}x_{2}$. The effectiveness of this function is 1.25, which reflects that, on average, selecting a prime implicant to describe the result of a given input constrains 1.25 variables (either ${\bar{x}}_{1}$ or ${\bar{x}}_{2}$ for the (0,0) state, ${\bar{x}}_{1}$ for the (0,1) state, ${\bar{X}}_{2}$ for the (1,0) state, and $x_{1}x_{2}$ for the (1,1) state, giving an average prime implicant size of $\frac{1+1+1+2}{4}=1.25$).

We compute $k_{e}$ using the Python library CANA [37], which does so using the look-up tables of *f*. See [20,28] for more details.
