# Supplementary material for: Effective Connectivity and Bias Entropy Improve Prediction of Dynamical Regime in Automata Networks
Source: Entropy (Basel). 2023 Feb 18;25(2):374. doi: 10.3390/e25020374 (PMC9955587; doi:10.3390/e25020374)
Supplement: Supplementary file 1 [file entropy-25-00374-s001.zip › entropy-2136519-supplementary.pdf]

results_for_manuscript

February 2, 2023

1 Effective Connectivity and Bias Entropy Improve Predition of Dyamical Regime in Automata Networks

1.1 Supplemental Notebook

1.1.1 Input data:

This notebook reads RBN metadata stored in folders `Results20`, `Results50`, `Results100`, and `Results200`, which are merged and stored in `full_RBN_data.csv` with some preprocessing. Cell Collective network data is also read from the `cc_network_data.csv` file.

The RBN results can be generated using the `PowerLaw_Experiment.py` script. Cell Collective summary data can be generated using the `cc_analysis.py` script.

1.1.2 Analysis and requirements:

All figures from the accompanying manuscript are reproducible by running this notebook. Additional figures are also produced here to supplement the main text. This notebook require `panda`, `numpy`, `sklearn`, `scipy`, and `matplotlib` to be installed.

We begin by importing various libraries and functions we will need for the analysis.

[ ]:
```
import pandas as pd
import numpy as np

import itertools

from os import listdir

from sklearn import metrics
from sklearn.metrics import RocCurveDisplay
from scipy import stats, special

import matplotlib as mpl
import matplotlib.pyplot as plt
from matplotlib.patches import Patch
from matplotlib.lines import Line2D
from matplotlib.colors import CenteredNorm
%matplotlib inline
```

2 RBN analysis

2.1 Data Import

We define various helper functions for preprocessing the data we will import.

[ ]:
```
def merge_results_frames(df,df2):
    for idx,r in df.iterrows():
        g = r['gamma']
        p = r['bias']
        for col in df.columns:
            if col == 'gamma' or col == 'bias':
                continue
            df.loc[(df['gamma']==g) & (df['bias']==p),col]+=df2.
 ↪loc[(df2['gamma']==g) & (df2['bias']==p),col]

def str2list(strarray):
    return [float(x) for x in strarray.strip('[] ').split()]

def df_str2list(df):
    for col in df.columns:
        if col == 'gamma' or col == 'bias':
            continue
        df[col] = df[col].apply(str2list)

def expand_shared_properties_in_dict(df):
    dictall = {}
    for _,row in df.iterrows():
        for col in df.columns:
            if col in ['gamma','bias']:
                rowlist = [row[col]]*len(row['Derrida'])
            else:
                rowlist = row[col]
            if col in dictall:
                dictall[col] += rowlist
            else:
                dictall[col] = rowlist.copy()
    return dictall

def append_extra_columns(dictall):
    dfall = pd.DataFrame(dictall)

    dfall['entropy'] = -(dfall['bias']*np.
 ↪log2(dfall['bias']))-((1-dfall['bias'])*np.log2(1-dfall['bias']))
    dfall['variance'] = dfall['bias']*(1-dfall['bias'])
```

```
    dfall['regime']=(dfall['Derrida']>1).astype(int) - (dfall['Derrida']<1).
 ↪astype(int)
    dfall['avgS']=dfall['avgKe']-dfall['avgKc']
    dfall['avgH'] = -dfall['avgP']*np.log2(dfall['avgP'])-(1-dfall['avgP'])*np.
 ↪log2(1-dfall['avgP'])
    dfall['avgV'] = (1-dfall['avgP'])*dfall['avgP']

    return dfall
```

We now import the RBN data.

Note that the variable `REGENERATE_DATA` determines whether the data will be read from the individual results folder to create a new `full_RBN_data.csv` file (if `True`) or read from from a previously assembled `full_RBN_data.csv` file (if `False`).

[ ]:
```
REGENERATE_DATA=False # takes a couple of minutes to regenerate data frame from␣
 ↪individual csv files

results_dir_dict={20:'Results20/',50:'Results50/',100:'Results100/',200:
 ↪'Results200/'}
if REGENERATE_DATA:
    dfall = pd.DataFrame()
    for N,results_dir in results_dir_dict.items():
        first_in_N = True
        for filename in listdir(results_dir):
            if first_in_N:
                df = pd.read_csv(results_dir+filename)
                df_str2list(df)
                first_in_N = False
            else:
                df2 = pd.read_csv(results_dir+filename)
                df_str2list(df2)
                merge_results_frames(df,df2)
        dictall = expand_shared_properties_in_dict(df)
        dfallN = append_extra_columns(dictall)
        dfallN['N']=N
        dfall = pd.concat([dfall,dfallN],axis=0)
    dfall.to_csv('full_RBN_data.csv')
else:
    dfall=pd.read_csv('full_RBN_data.csv')
Nvals = results_dir_dict.keys()
dfall['gamma'] = dfall['gamma'].round(2) # to fix some entries recorded, e.g.,␣
 ↪like 2.100000005
dfall['bias'] = dfall['bias'].round(2)
# shuffle data
dfall=dfall.sample(frac=1.0)
```

```
dfall
```

[ ]:

```
        Unnamed: 0  gamma  bias  Derrida   avgK  medK     avgKe  medKe  \
40437         4437    1.6  0.15    0.611  2.560   1.0  1.133424    0.0
123590       15590    1.9  0.15    0.531  2.105   1.0  0.878037    0.0
129078       21078    2.0  0.40    1.095  2.155   1.0  1.306785    1.0
104475       32475    2.4  0.05    0.219  1.700   1.0  0.354121    0.0
9022          9022    1.7  0.25    0.765  1.850   1.0  0.939621    1.0
…              …   …    …     …   …   …       …    …
51485        15485    1.9  0.15    0.430  2.260   1.0  0.854496    0.0
93813        21813    2.1  0.05    0.210  2.250   1.0  0.514603    0.0
124505       16505    1.9  0.30    0.946  2.225   1.0  1.333862    1.0
100708       28708    2.2  0.45    0.913  1.820   1.0  1.107765    1.0
122934       14934    1.9  0.10    0.470  2.305   1.0  0.882323    0.0

           avgKc  medKc      avgP   entropy  variance  regime      avgS  \
40437   0.512113    0.0  0.151626  0.609840    0.1275      -1  0.621311
123590  0.345137    0.0  0.150616  0.609840    0.1275      -1  0.532900
129078  0.296745    0.0  0.397539  0.970951    0.2400       1  1.010040
104475  0.145254    0.0  0.054072  0.286397    0.0475      -1  0.208867
9022    0.167746    0.0  0.213333  0.811278    0.1875      -1  0.771875
…            …   …       …       …       …      …   …
51485   0.394574    0.0  0.147890  0.609840    0.1275      -1  0.459922
93813   0.331315    0.0  0.048848  0.286397    0.0475      -1  0.183289
124505  0.344590    0.0  0.299920  0.881291    0.2100      -1  0.989271
100708  0.205949    0.0  0.445099  0.992774    0.2475      -1  0.901816
122934  0.445789    0.0  0.101062  0.468996    0.0900      -1  0.436534

            avgH      avgV    N
40437   0.613893  0.128635   50
123590  0.611380  0.127931  200
129078  0.969493  0.239502  200
104475  0.303449  0.051148  100
9022    0.747806  0.167822   20
…            …       …  …
51485   0.604534  0.126018   50
93813   0.281481  0.046461  100
124505  0.881194  0.209968  200
100708  0.991286  0.246986  100
122934  0.472354  0.090849  200

[144000 rows x 18 columns]
```

We define a helper function for finding the critical boundary.

The thermodynamic critical boundary depends upon the average in-degree. We therefore define a function to calculate this mean (in the thermodynamic limit) from the generateing parameter `gamma` (γ) assuming a truncated power-law distribution with exponent γ and cutoff m (here explicitly

called `cutoff`).

[ ]:
```
def kmean(gamma,cutoff=None):
    if cutoff is None:
        return special.zeta(gamma-1)/special.zeta(gamma)
    else:
        ks = np.arange(1,cutoff+1).astype(float)
        return np.sum(ks**(1-gamma)) / np.sum(ks**(-gamma))
```

2.2 Finite Size and the Critical Boundary

We explore how finite-size effects influence the critical boundary.

The figures produced here show the fraction of networks for each sampled point in parameter space that have Derrida coefficients (δ) greater than 1 (chaotic, in red) or less than 1 (ordered, in blue). Bold outlined points have at least a $15-85\%$ split between ordered and chaotic networks. These allow us to visualize a "fuzzy" critical boundary for various sizes of networks.

[ ]:
```
biases = np.arange(0.05,0.5,0.05).round(2)
gammas = np.arange(1.5,2.5,0.1).round(2)
fs=32
fsa=24
fsl=20
for N in Nvals:
    fig, ax = plt.subplots(figsize=(10, 10),facecolor='white')

    xvals = np.arange(1.4, 2.6, 0.005)
    ax.plot(xvals,[0.5-0.5*np.sqrt(1-2/kmean(xg,cutoff=15)) for xg in␣
 ↪xvals],'k--')

    for g,p in itertools.product(gammas,biases):
        g=g.round(2)
        p=p.round(2)
        ones = (dfall[(dfall['N'] == N) & (dfall['gamma'] == g) &␣
 ↪(dfall['bias'] == p)]['Derrida']>1).mean()
        ax.pie([ones, 1-ones], center=(g, p), radius=0.01, colors=['r', 'b'],␣
 ↪frame=True)
        if ones > 0.85 or ones < 0.15:
            ax.pie([ones, 1-ones], center=(g, p), radius=0.01, colors=['r',␣
 ↪'b'], frame=True)
        else:
            ax.pie([ones, 1-ones], center=(g, p), radius=0.013, colors=['k',␣
 ↪'k'], frame=True)
            ax.pie([ones, 1-ones], center=(g, p), radius=0.01, colors=['r',␣
 ↪'b'], frame=True)
        ax.set_xlim(1.45,2.45)
        ax.set_ylim(0,0.5)
```

```
legend_elements = [Patch(facecolor='r', label='Chaotic'),␣
↪Patch(facecolor='b', label='Ordered'),␣
↪Line2D([0],[0],color='k',linestyle='--',label=r'$N\rightarrow\infty$␣
↪critical boundary')]
plt.title(f'$N=${N}',fontsize=fs)
plt.xlabel('$\gamma$',fontsize=fs)
plt.ylabel('$P$',fontsize=fs)
ax.tick_params(axis='both', which='major', labelsize=fsa)
ax.legend(handles=legend_elements, loc='lower right',fontsize=fsl)
plt.savefig(f'figures/PieChartFigure_{N}.pdf',bbox_inches='tight')
plt.savefig(f'figures/PieChartFigure_{N}.png',bbox_inches='tight')
plt.show()
```

```
/tmp/ipykernel_35093/1460200497.py:10: RuntimeWarning: invalid value encountered
in sqrt
  ax.plot(xvals,[0.5-0.5*np.sqrt(1-2/kmean(xg,cutoff=15)) for xg in
xvals],'k--')
```

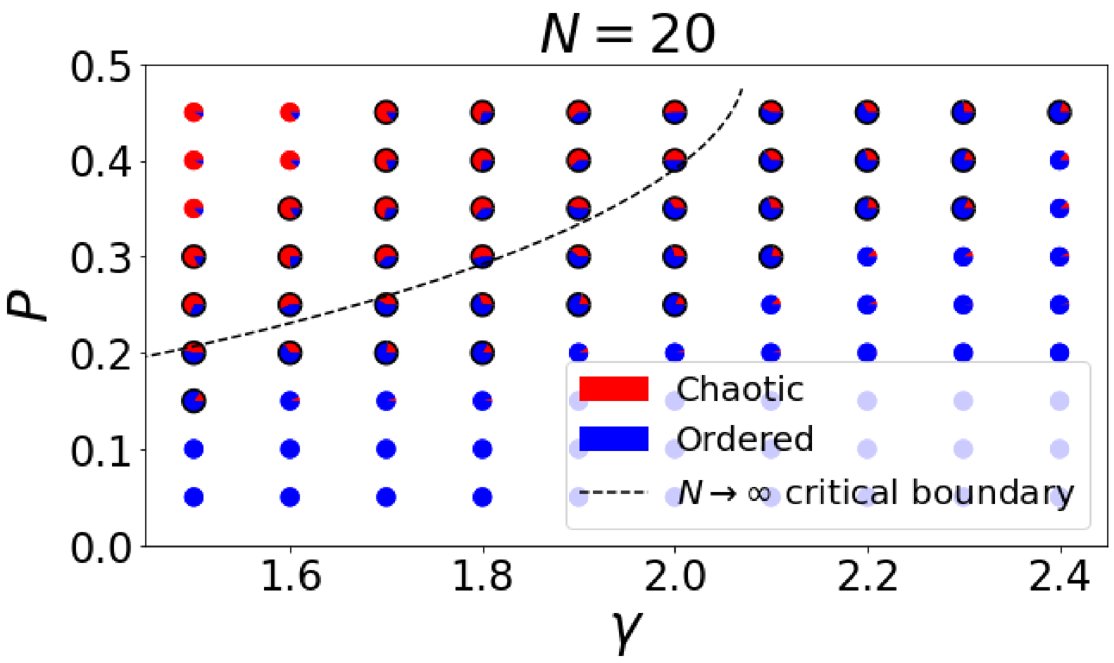


```
/tmp/ipykernel_35093/1460200497.py:10: RuntimeWarning: invalid value encountered
in sqrt
  ax.plot(xvals,[0.5-0.5*np.sqrt(1-2/kmean(xg,cutoff=15)) for xg in
xvals],'k--')
```

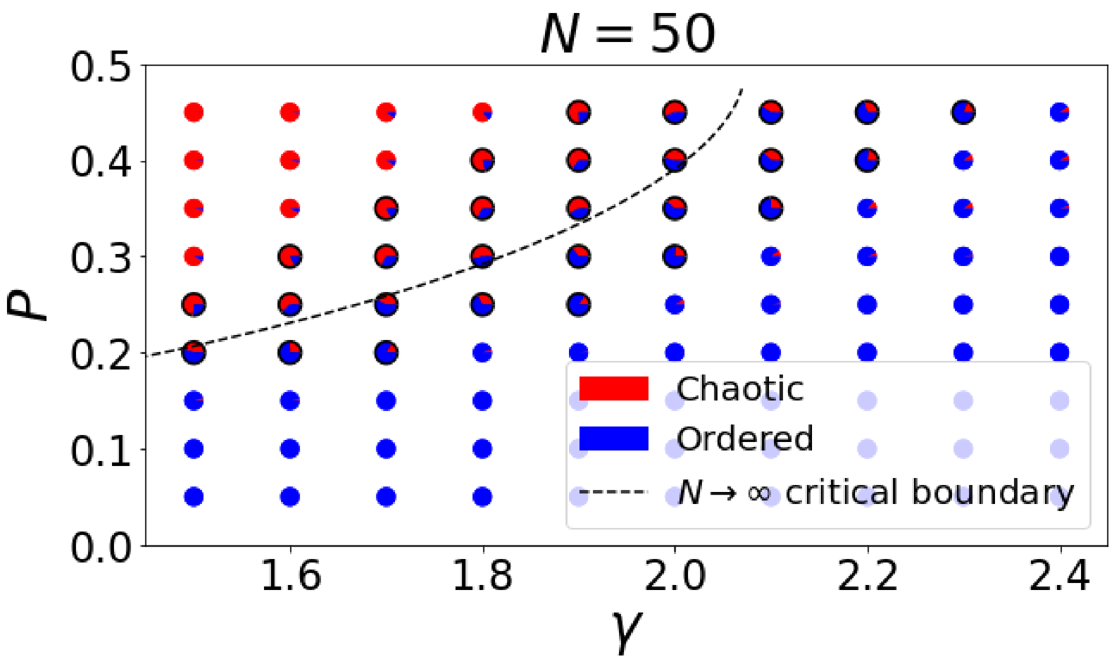


```
/tmp/ipykernel_35093/1460200497.py:10: RuntimeWarning: invalid value encountered
in sqrt
  ax.plot(xvals,[0.5-0.5*np.sqrt(1-2/kmean(xg,cutoff=15)) for xg in
xvals],'k--')
```

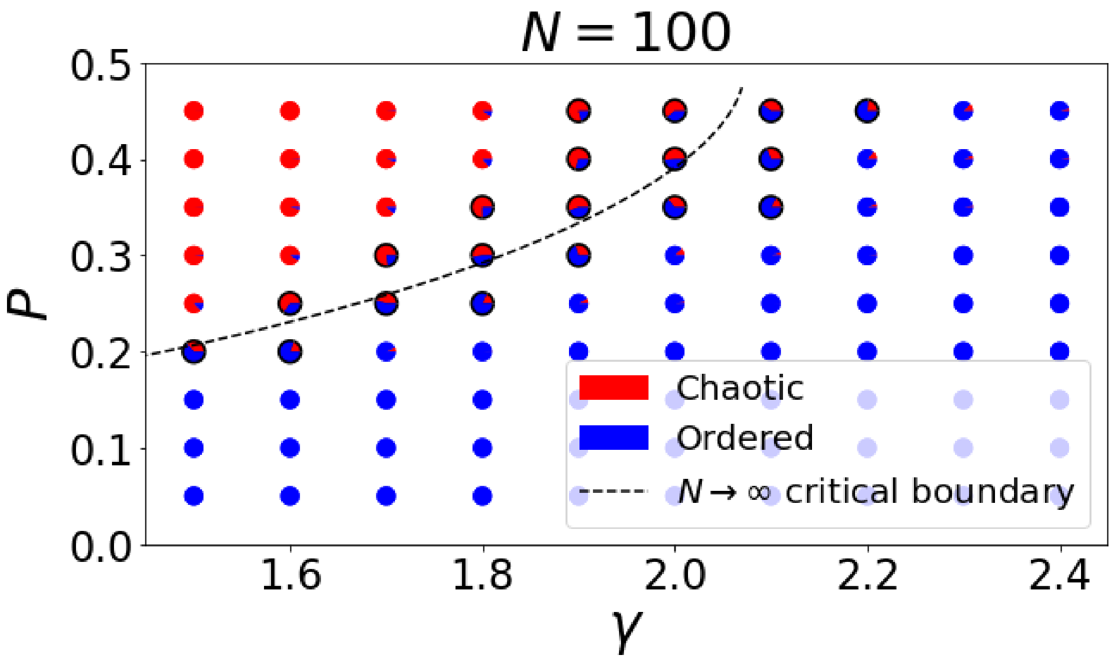

```
/tmp/ipykernel_35093/1460200497.py:10: RuntimeWarning: invalid value encountered
in sqrt
  ax.plot(xvals,[0.5-0.5*np.sqrt(1-2/kmean(xg,cutoff=15)) for xg in
xvals],'k--')
```

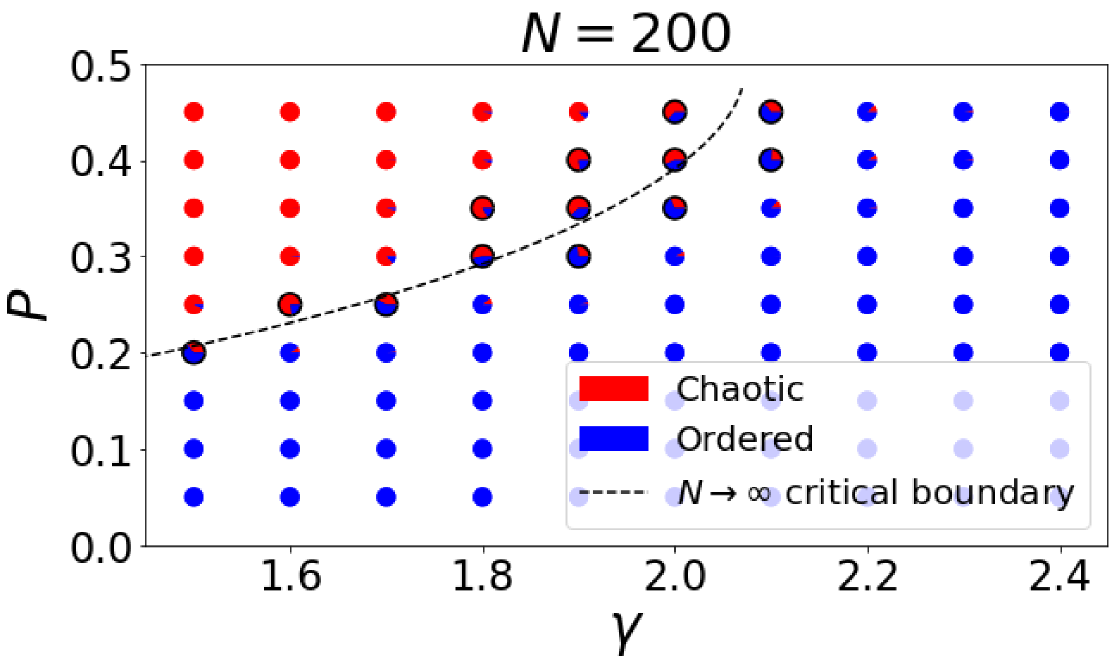

Figure S1: Proportion of chaotic (red) and ordered (blue) networks from the 400 samples at each point in the $P-\gamma$ parameter space for two values of N. The dashed curve is the critical boundary in the thermodynamic limit, $N \to \infty$. Black borders are added to the points for which between 15% and 85% of networks are chaotic (or, equivalently, ordered). These points form a critical region that shrinks as N increases, apparently converging to the thermodynamic critical boundary. (See Figure 1 in main text)

As expected, the fuzzy boundary becomes sharper as the size of the networks increases, and the boundary appears to be converging to the theoretical prediciton.

2.3 Fitting the Critical Boundary

We consider various ways to fit the critical boundary. We begin by defining the functional forms we will attempt to fit to and associated helper functions.

[ ]:
```
from scipy.optimize import curve_fit
def powerlaw(x,c,m,b):
    return c*x**m + b

def linear_law(x, m, b):
    return m*x + b
```

```
def critical_kappa(rho,c,m,b):
    return (1/rho)*((1-b)/c)**(1/m)

def critical_kappa_lin(rho,m,b):
    return (1-b)/(m*rho)

def mse(x,y,f):
    return np.nanmean((f(x)-y)**2)
```

We fit power law functions to the critical boundary in RBNs.

As described in the main text, we fit $\sigma^2 k$, $\sigma^2 k_e$, and Hk_e to δ using a power law function for various sizes of network.

[ ]:
```
fs = 36
fsl = 28
fsa = 28
for N in Nvals:
    dfN = dfall[dfall['N']==N]

    fig, ax = plt.subplots(1,3,figsize=(30, 10),facecolor='white')
    cmap = plt.cm.get_cmap('coolwarm')#.reversed()
    x=dfN['avgK']*dfN['avgV']
    y=dfN['Derrida']
    popt, pcov = curve_fit(powerlaw,x[np.isfinite(x)],dfN['Derrida'][np.
 ↪isfinite(x)])
    c, m, b = popt
    xfit=np.arange(x.min(),x.max(),0.01)
    yfit=powerlaw(xfit,c,m,b)
    gof = mse(x,dfN['Derrida'],lambda xi: powerlaw(xi,c,m,b))
    mstr='{'+str(np.round(m,2))+'}'
    label = f'${np.round(c,2)}(\sigma^2 k)^{mstr}{np.round(b,2):+}$\n$MSE={np.
 ↪round(gof,5)}$'
    ax[0].plot(xfit,yfit,'k--', label=label)
    sc=ax[0].scatter(x, y, s=1,
                     c=dfN['Derrida'], cmap=cmap,
                     norm=CenteredNorm(vcenter=1,halfrange=0.5))
    ax[0].set_xlabel("$\sigma^2 k$",fontsize=fs)
    ax[0].set_ylabel("$\delta$",fontsize=fs)
    ax[0].tick_params(axis='both', which='major', labelsize=fsa)
    ax[0].legend(fontsize=fsl, loc = 'lower right')
    ax[0].set_ylim(0,3)
    ax[0].set_xlim(0,1.5)

    x=dfN['avgKe']*dfN['avgV']
    popt, pcov = curve_fit(powerlaw,x[np.isfinite(x)],dfN['Derrida'][np.
 ↪isfinite(x)])
```

```
    c, m, b = popt
    xfit=np.arange(x.min(),x.max(),0.01)
    yfit=powerlaw(xfit,c,m,b)
    gof = mse(x,dfN['Derrida'],lambda xi: powerlaw(xi,c,m,b))
    mstr='{'+str(np.round(m,2))+'}'
    label = f'${np.round(c,2)}(\sigma^2 k_e)^{mstr}{np.round(b,2):+}$\n$MSE={np.
↪round(gof,5)}$'
    ax[1].plot(xfit,yfit,'k--', label=label)
    sc=ax[1].scatter(x, y, s=1,
                     c=dfN['Derrida'], cmap=cmap,
                     norm=CenteredNorm(vcenter=1,halfrange=0.5))
    ax[1].set_xlabel("$\sigma^2 k_e$",fontsize=fs)
    ax[1].set_ylabel("$\delta$",fontsize=fs)
    ax[1].tick_params(axis='both', which='major', labelsize=fsa)
    ax[1].legend(fontsize=fsl, loc = 'lower right')
    ax[1].set_ylim(0,3)
    ax[1].set_xlim(0,1.2)

    x=dfN['avgKe']*dfN['avgH']
    popt, pcov = curve_fit(powerlaw,x[np.isfinite(x)],dfN['Derrida'][np.
↪isfinite(x)])
    c, m, b = popt
    xfit=np.arange(x.min(),x.max(),0.01)
    yfit=powerlaw(xfit,c,m,b)
    gof = mse(x,dfN['Derrida'],lambda xi: powerlaw(xi,c,m,b))
    mstr='{'+str(np.round(m,2))+'}'
    label = f'${np.round(c,2)}(Hk_e)^{mstr}{np.round(b,2):+}$\n$MSE={np.
↪round(gof,5)}$'
    ax[2].plot(xfit,yfit,'k--',label=label)
    sc=ax[2].scatter(x, y, s=1,
                     c=dfN['Derrida'], cmap=cmap,
                     norm=CenteredNorm(vcenter=1,halfrange=0.5))
    ax[2].set_xlabel("$Hk_e$",fontsize=fs)
    ax[2].set_ylabel("$\delta$",fontsize=fs)
    ax[2].tick_params(axis='both', which='major', labelsize=fsa)
    ax[2].legend(fontsize=fsl, loc = 'lower right')
    ax[2].set_ylim(0,3)
    ax[2].set_xlim(0,5)

    cbar=fig.colorbar(sc,ax=ax,location='bottom',aspect=75)
    cbar.ax.tick_params(axis='both',labelsize=fs)
    cbar.set_label('Derrida Coefficient ($\delta$)',fontsize=fs)
    plt.suptitle(f'Power law fits ({N=})',fontsize=fs)
#     plt.savefig(f'figures/PowerLawFits_RBN_{N}.pdf',bbox_inches='tight')
    plt.savefig(f'figures/PowerLawFits_RBN_{N}.png',bbox_inches='tight')
    plt.show()
```

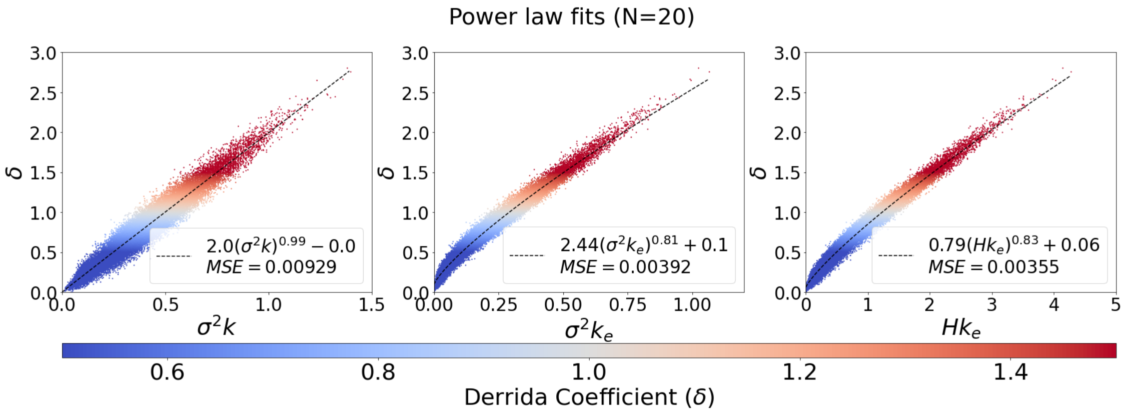
Power law fits (N=20)
$2.0(\sigma^2 k)^{0.99} - 0.0$
$MSE = 0.00929$
$2.44(\sigma^2 k_e)^{0.81} + 0.1$
$MSE = 0.00392$
$0.79(Hk_e)^{0.83} + 0.06$
$MSE = 0.00355$
δ
$\sigma^2 k$
$\sigma^2 k_e$
Hk_e
Derrida Coefficient (δ)

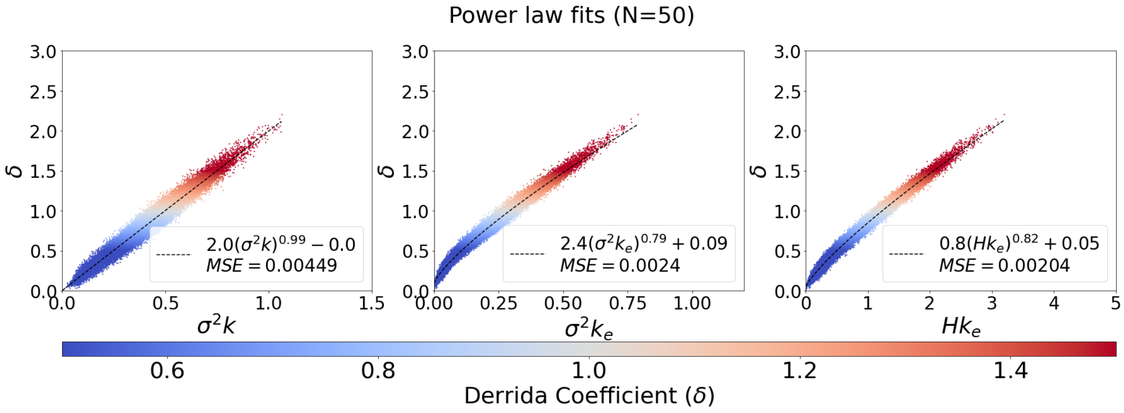
Power law fits (N=50)
$2.0(\sigma^2 k)^{0.99} - 0.0$
$MSE = 0.00449$
$2.4(\sigma^2 k_e)^{0.79} + 0.09$
$MSE = 0.0024$
$0.8(Hk_e)^{0.82} + 0.05$
$MSE = 0.00204$
δ
$\sigma^2 k$
$\sigma^2 k_e$
Hk_e
Derrida Coefficient (δ)

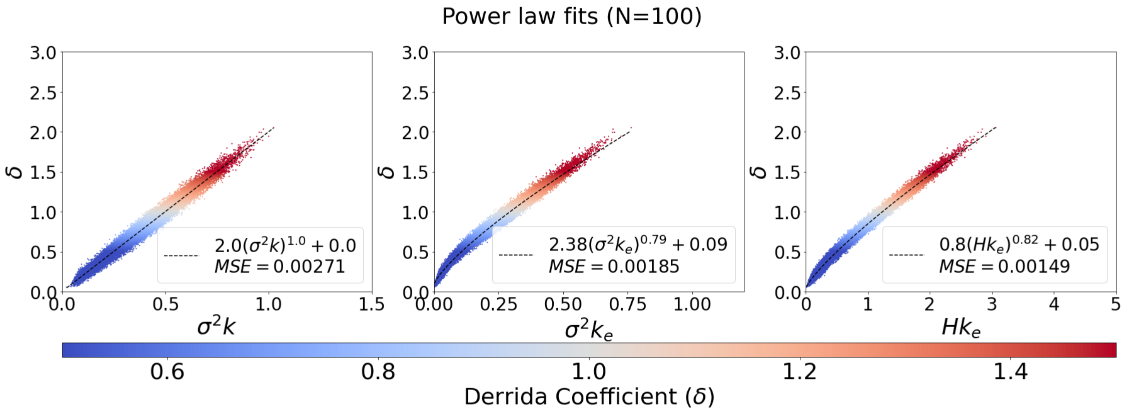
Power law fits (N=100)
$2.0(\sigma^2 k)^{1.0} + 0.0$
$MSE = 0.00271$
$2.38(\sigma^2 k_e)^{0.79} + 0.09$
$MSE = 0.00185$
$0.8(Hk_e)^{0.82} + 0.05$
$MSE = 0.00149$
δ
$\sigma^2 k$
$\sigma^2 k_e$
Hk_e
Derrida Coefficient (δ)

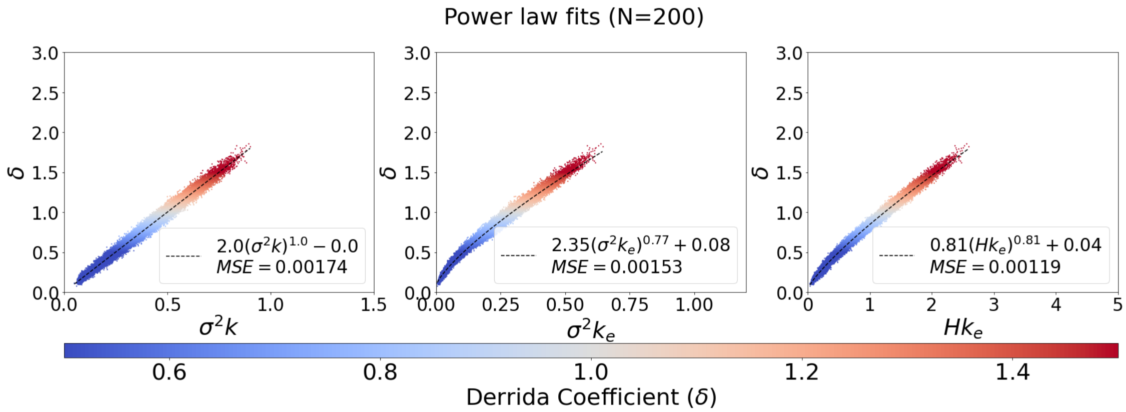

Figure S2: Ability of each measure to predict the Derrida coefficient of heterogeneous random networks. Each point corresponds to a sampled network, and its color indicates the network's average node sensitivity. The curves are obtained by fitting a power law function of various measures to the Derrida coefficient, δ: $kp(1-p)$ to the δ (left), $k_e p(1-p)$ to δ (center), and Hk_e to δ (right). Plots combine networks sampled from all considered P and γ parameter values; thus each plot depicts $36,000$ networks.

Note a small but consistent improvement in the mean squared error when moving from left to right in the above figures.

We repeat the procedure using a partial linear fit.

The values of δ for the partial fit are chosen so that the data are approximately linear in all cases and for agreement with values calculated for empirical models, which will be analyzed later.

[ ]:
```
fs = 36
fsl = 28
fsa = 28
for N in Nvals:
    dfN = dfall[dfall['N']==N]
    dselect = (1.5>dfN['Derrida']) & (dfN['Derrida']>0.5)

    fig, ax = plt.subplots(1,3,figsize=(30, 10),facecolor='white')
    cmap = plt.cm.get_cmap('coolwarm')#.reversed()
    x=dfN['avgK']*dfN['avgV'] / (dselect)
    y=dfN['Derrida']
    popt, pcov = curve_fit(linear_law,x[np.isfinite(x)],dfN['Derrida'][np.
↪isfinite(x)])
    x=dfN['avgK']*dfN['avgV']
    m, b = popt
    xfit=np.arange(x.min(),x.max(),0.01)
    yfit=linear_law(xfit,m,b)
    gof = mse(x[dselect],dfN['Derrida'][dselect],lambda xi: linear_law(xi,m,b))
```

```
    label = f'${np.round(m,2)}(\sigma^2 k){np.round(b,2):+}$\n$MSE={np.
↪round(gof,5)}$'
    ax[0].plot(xfit,yfit,'k--', label=label)
    sc=ax[0].scatter(x, y, s=1,
                     c=dfN['Derrida'], cmap=cmap,
                     norm=CenteredNorm(vcenter=1,halfrange=0.5))
    ax[0].set_xlabel("$\sigma^2 k$",fontsize=fs)
    ax[0].set_ylabel("$\delta$",fontsize=fs)
    ax[0].tick_params(axis='both', which='major', labelsize=fsa)
    ax[0].legend(fontsize=fsl, loc = 'lower right')
    ax[0].set_ylim(0,3)
    ax[0].set_xlim(0,1.5)

    x=dfN['avgKe']*dfN['avgV'] / (dselect)
    popt, pcov = curve_fit(linear_law,x[np.isfinite(x)],dfN['Derrida'][np.
↪isfinite(x)])
    x=dfN['avgKe']*dfN['avgV']
    m, b = popt
    xfit=np.arange(x.min(),x.max(),0.01)
    yfit=linear_law(xfit,m,b)
    gof = mse(x[dselect],dfN['Derrida'][dselect],lambda xi: linear_law(xi,m,b))
    label = f'${np.round(m,2)}(\sigma^2 k_e){np.round(b,2):+}$\n$MSE={np.
↪round(gof,5)}$'
    ax[1].plot(xfit,yfit,'k--', label=label)
    sc=ax[1].scatter(x, y, s=1,
                     c=dfN['Derrida'], cmap=cmap,
                     norm=CenteredNorm(vcenter=1,halfrange=0.5))
    ax[1].set_xlabel("$\sigma^2 k_e$",fontsize=fs)
    ax[1].set_ylabel("$\delta$",fontsize=fs)
    ax[1].tick_params(axis='both', which='major', labelsize=fsa)
    ax[1].legend(fontsize=fsl, loc = 'lower right')
    ax[1].set_ylim(0,3)
    ax[1].set_xlim(0,1.2)

    x=dfN['avgKe']*dfN['avgH']
    x=dfN['avgKe']*dfN['avgH'] / (dselect)
    popt, pcov = curve_fit(linear_law,x[np.isfinite(x)],dfN['Derrida'][np.
↪isfinite(x)])
    x=dfN['avgKe']*dfN['avgH']
    m, b = popt
    xfit=np.arange(x.min(),x.max(),0.01)
    yfit=linear_law(xfit,m,b)
    gof = mse(x[dselect],dfN['Derrida'][dselect],lambda xi: linear_law(xi,m,b))
    label = f'${np.round(m,2)}(Hk_e){np.round(b,2):+}$\n$MSE={np.round(gof,5)}$'
    ax[2].plot(xfit,yfit,'k--',label=label)
    sc=ax[2].scatter(x, y, s=1,
                     c=dfN['Derrida'], cmap=cmap,
```

```
                          norm=CenteredNorm(vcenter=1,halfrange=0.5))
    ax[2].set_xlabel("$Hk_e$",fontsize=fs)
    ax[2].set_ylabel("$\delta$",fontsize=fs)
    ax[2].tick_params(axis='both', which='major', labelsize=fsa)
    ax[2].legend(fontsize=fsl, loc = 'lower right')
    ax[2].set_ylim(0,3)
    ax[2].set_xlim(0,5)

    cbar=fig.colorbar(sc,ax=ax,location='bottom',aspect=75)
    cbar.ax.tick_params(axis='both',labelsize=fs)
    cbar.set_label('Derrida Coefficient ($\delta$)',fontsize=fs)
    plt.suptitle(f'Linear fits ({N=}), $1.5>\delta > 0.5$ only',fontsize=fs)
#     plt.savefig(f'figures/LinearFits_RBN_{N}.pdf',bbox_inches='tight')
    plt.savefig(f'figures/LinearFits_RBN_{N}.png',bbox_inches='tight')
    plt.show()
```

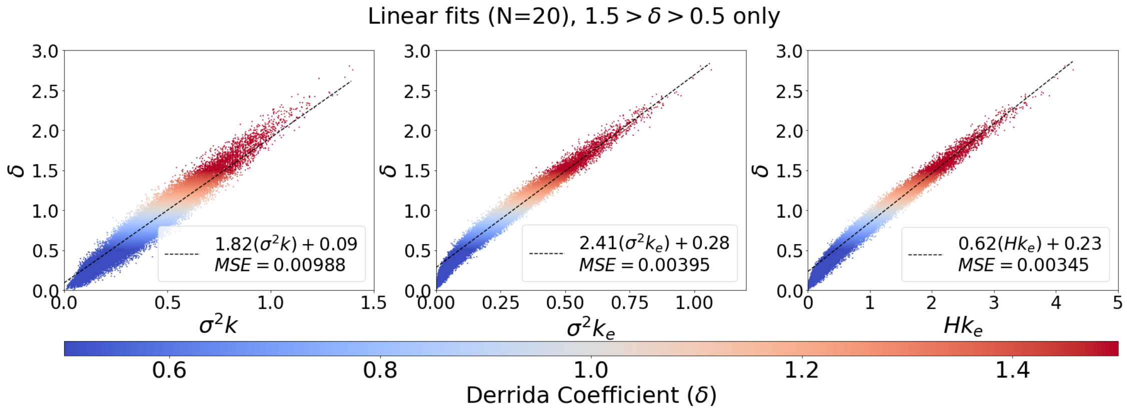

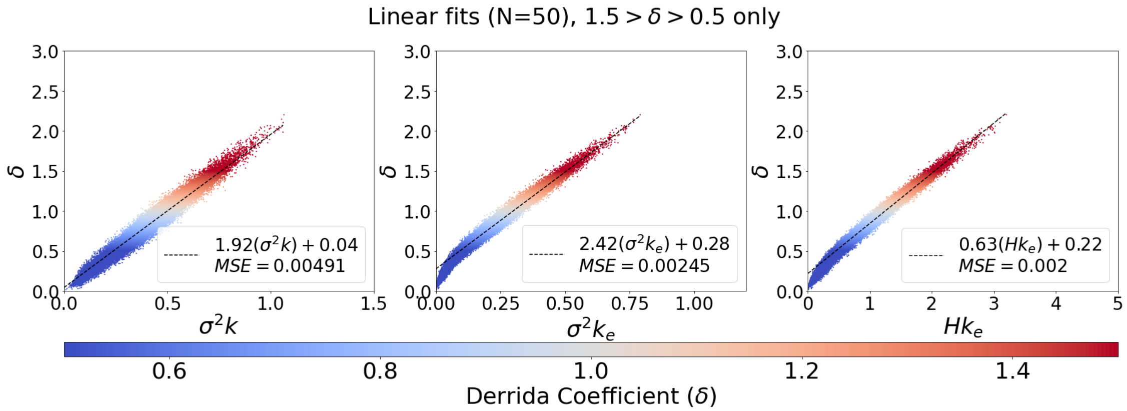

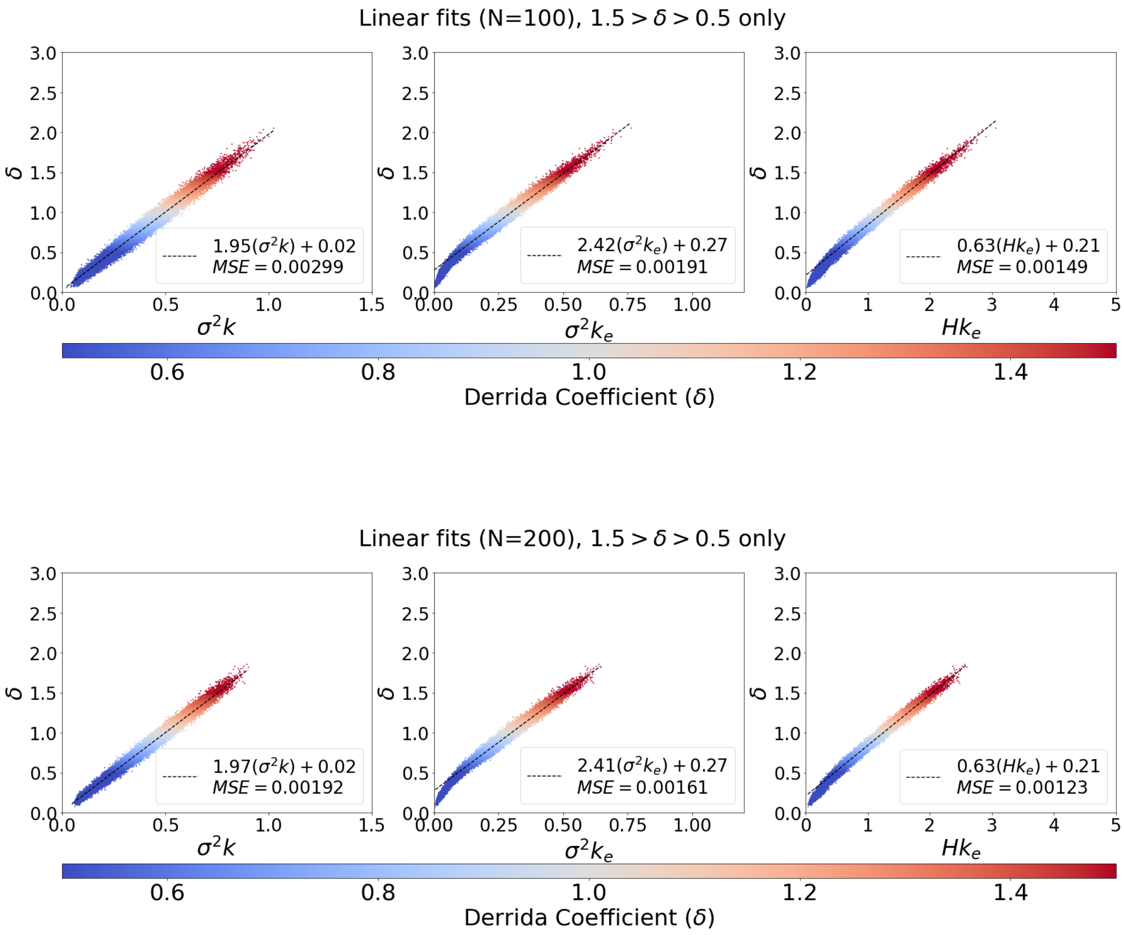

Figure S3: Ability of each measure to predict the Derrida coefficient of heterogeneous random networks. Each point corresponds to a sampled network, and its color indicates the network's average node sensitivity. The curves are obtained by fitting a linear function of various measures to a range of the Derrida coefficient, $0.5 < \delta < 1.5$: $kp(1-p)$ to the δ (left), $k_e p(1-p)$ to δ (center), and Hk_e to δ (right). Plots combine networks sampled from all considered P and γ parameter values; thus each plot depicts $36,000$ networks.

Again, there is a small but consistent improvement in mean squared error when moving from the left to the right.

2.4 Power Law Fits in the Connectivity/Bias-Spread Planes.

[ ]:
```
fs = 36
fsa = 28
for N in Nvals:
    dfN = dfall[dfall['N']==N]

    fig, ax = plt.subplots(1,3,figsize=(30, 10),facecolor='white')
```

```
    cmap = plt.cm.get_cmap('coolwarm')#.reversed()
    x=dfN['avgK']*dfN['avgV']
    y=dfN['Derrida']
    popt, pcov = curve_fit(powerlaw,x[np.isfinite(x)],dfN['Derrida'][np.
↪isfinite(x)])
    c, m, b = popt
    xfit=np.arange(0.005,0.255,0.005)
    yfit=critical_kappa(xfit,c,m,b)
    label = 'estimated critical boundary'
    ax[0].plot(xfit,yfit,'k--', label=label)
    sc=ax[0].scatter(dfN['avgV'], dfN['avgK'], s=1,
                     c=dfN['Derrida'], cmap=cmap,
                     norm=CenteredNorm(vcenter=1,halfrange=0.5))
    ax[0].set_xlabel("$\sigma^2$",fontsize=fs)
    ax[0].set_ylabel("$k$",fontsize=fs)
    ax[0].tick_params(axis='both', which='major', labelsize=fsa)
    #ax[0].legend(fontsize=fs, loc = 'lower right')
    ax[0].set_xlim(0,0.25)
    ax[0].set_ylim(1,5)


    x=dfN['avgKe']*dfN['avgV']
    popt, pcov = curve_fit(powerlaw,x[np.isfinite(x)],dfN['Derrida'][np.
↪isfinite(x)])
    c, m, b = popt
    xfit=np.arange(0.01,0.255,0.005)
    yfit=critical_kappa(xfit,c,m,b)
    label = 'estimated critical boundary'
    ax[1].plot(xfit,yfit,'k--', label=label)
    sc=ax[1].scatter(dfN['avgV'], dfN['avgKe'], s=1,
                     c=dfN['Derrida'], cmap=cmap,
                     norm=CenteredNorm(vcenter=1,halfrange=0.5))
    ax[1].set_xlabel("$\sigma^2$",fontsize=fs)
    ax[1].set_ylabel("$k_e$",fontsize=fs)
    ax[1].tick_params(axis='both', which='major', labelsize=fsa)
    #ax[1].legend(fontsize=fs, loc = 'lower right')
    ax[1].set_ylim(0,3.5)
    ax[1].set_xlim(0,0.25)

    x=dfN['avgKe']*dfN['avgH']
    popt, pcov = curve_fit(powerlaw,x[np.isfinite(x)],dfN['Derrida'][np.
↪isfinite(x)])
    c, m, b = popt
    xfit=np.arange(x.min(),x.max(),0.01)
    xfit=np.arange(0.01,1.01,0.01)
    yfit=critical_kappa(xfit,c,m,b)
    label = 'estimated critical boundary'
```

```
    ax[2].plot(xfit,yfit,'k--',label=label)
    sc=ax[2].scatter(dfN['avgH'], dfN['avgKe'], s=1,
                     c=dfN['Derrida'], cmap=cmap,
                     norm=CenteredNorm(vcenter=1,halfrange=0.5))
    ax[2].set_xlabel("$H$",fontsize=fs)
    ax[2].set_ylabel("$k_e$",fontsize=fs)
    ax[2].tick_params(axis='both', which='major', labelsize=fsa)
    #ax[2].legend(fontsize=fs, loc = 'lower right')
    ax[2].set_ylim(0,3.5)
    ax[2].set_xlim(0,1)

    cbar=fig.colorbar(sc,ax=ax,location='bottom',aspect=75)
    cbar.ax.tick_params(axis='both',labelsize=fs)
    cbar.set_label('Derrida Coefficient ($\delta$)',fontsize=fs)
    plt.suptitle(f'Power law fits ({N=})',fontsize=fs)
#     plt.savefig(f'figures/PowerLawFitsAlt_RBN_{N}.pdf',bbox_inches='tight')
    plt.savefig(f'figures/PowerLawFitsAlt_RBN_{N}.png',bbox_inches='tight')
    plt.show()
```

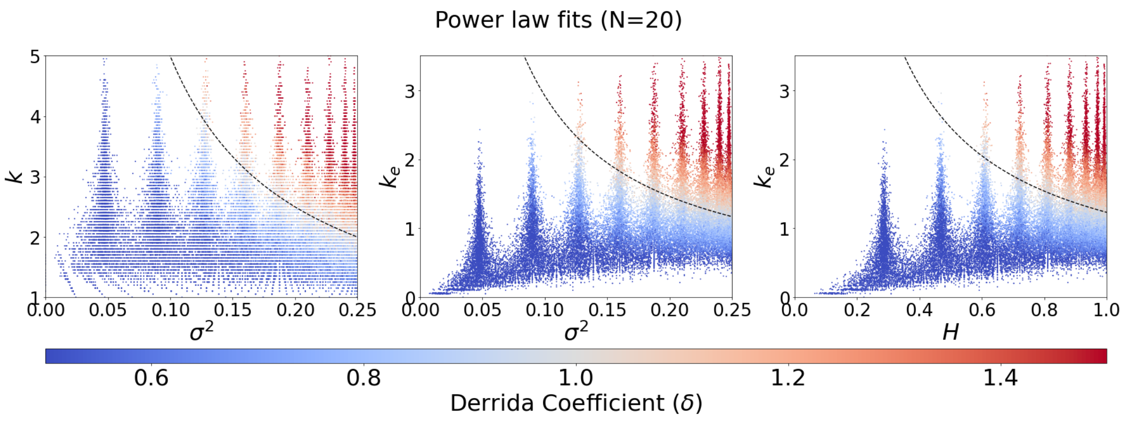

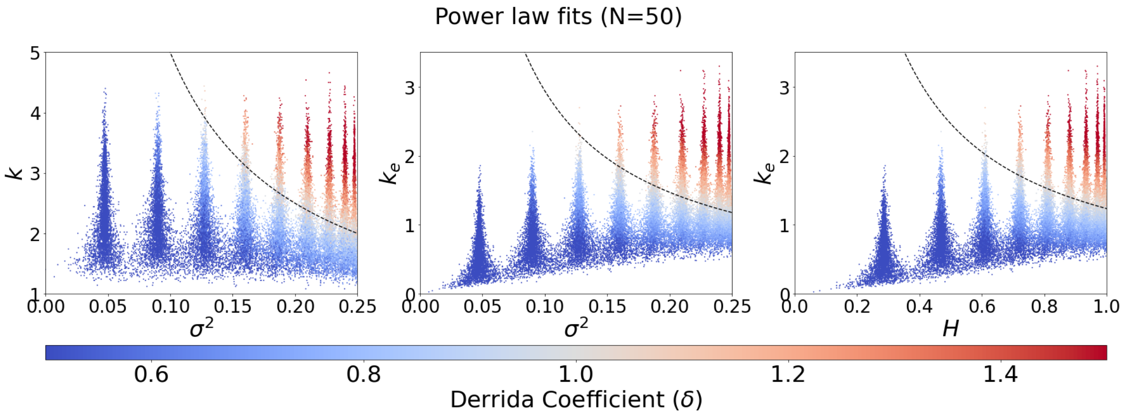

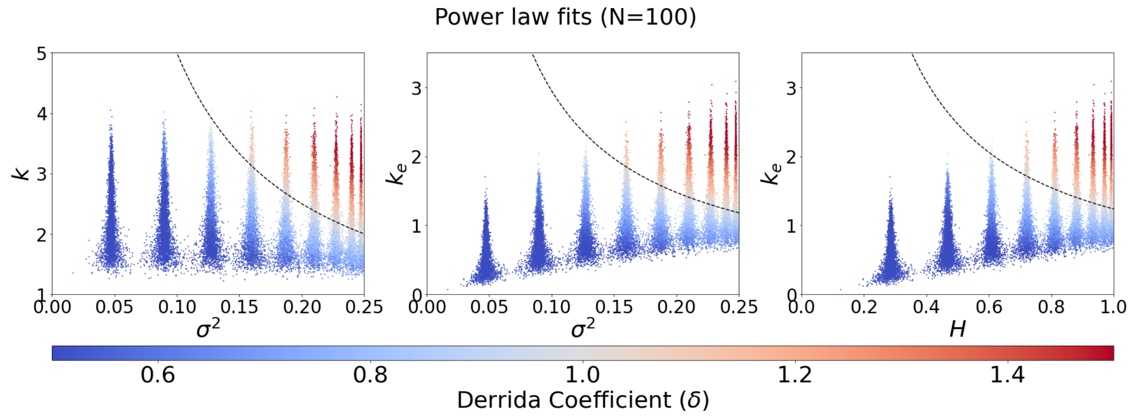

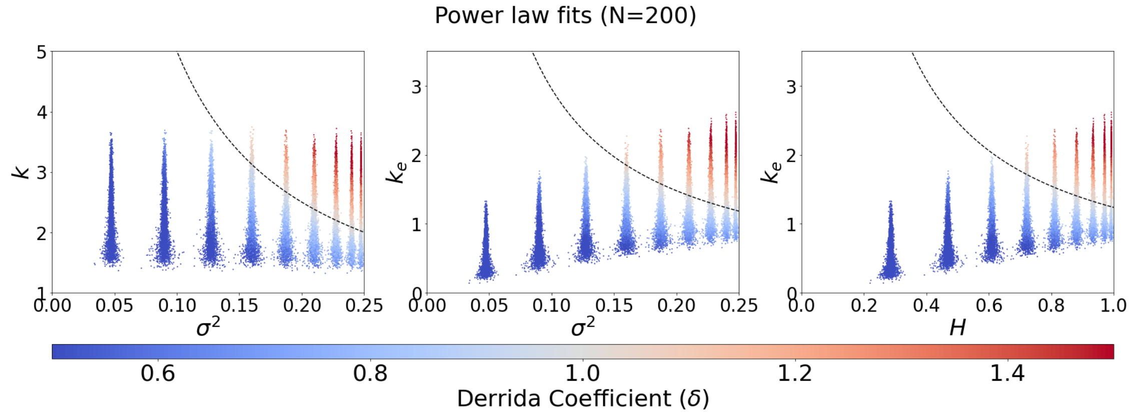

Figure S4: Critical boundaries obtained from fitting connectivity-spread products to the Derrida coefficient. The color of each node indicates the network's Derrida coefficient. The critical boundary (dashed curve) is obtained by setting the power law fit found in *Figure S3* to 1 and inverting for k (left) or k_e (center and right).

2.5 Dynamical Regime Classification Confusion Matrices

Next, we examine confusion matrices describing the ability of these critical boundaries to discriminate between dynamical regimes

[ ]:
```
fs = 24
for N in Nvals:
    dfN = dfall[dfall['N']==N]

    fig, ax = plt.subplots(2, 2, figsize=(10, 10), sharey='row', sharex='col',␣
    ↪facecolor='white')
    cmap = plt.cm.get_cmap('coolwarm')#.reversed()

    #x=dfN['avgS']
```

```
    x=dfN['Derrida']
    y=dfN['Derrida']
    popt, pcov = curve_fit(powerlaw,x[np.isfinite(x)],dfN['Derrida'][np.
↪isfinite(x)])
    c, m, b = popt
    yfit=powerlaw(x,1,1,0)
    truth = (y>1).astype(int) - (y<1).astype(int)
    preds = (yfit>1).astype(int)-(yfit<1).astype(int)
    confusion_matrix = metrics.confusion_matrix(truth,preds)
    cm_display = metrics.ConfusionMatrixDisplay(confusion_matrix =␣
↪confusion_matrix, display_labels = ['Ordered', 'Critical', 'Chaotic'])
    cm_display.plot(ax=ax[0,0])
    cm_display.im_.colorbar.remove()
    ax[0,0].set_title('Sensitivity')

    x=dfN['avgK']*dfN['avgV']
    y=dfN['Derrida']
    popt, pcov = curve_fit(powerlaw,x[np.isfinite(x)],dfN['Derrida'][np.
↪isfinite(x)])
    c, m, b = popt
    yfit=powerlaw(x,c,m,b)
    truth = (y>1).astype(int) - (y<1).astype(int)
    preds = (yfit>1).astype(int)-(yfit<1).astype(int)
    confusion_matrix = metrics.confusion_matrix(truth,preds)
    cm_display = metrics.ConfusionMatrixDisplay(confusion_matrix =␣
↪confusion_matrix, display_labels = ['Ordered', 'Critical', 'Chaotic'])
    cm_display.plot(ax=ax[0,1])
    cm_display.im_.colorbar.remove()
    mstr='{'+str(np.round(m,2))+'}'
    label = f'${np.round(c,2)}(\sigma^2 k)^{mstr}+{np.round(b,2)}$'
    ax[0,1].set_title(label)

    x=dfN['avgKe']*dfN['avgV']
    y=dfN['Derrida']
    popt, pcov = curve_fit(powerlaw,x[np.isfinite(x)],dfN['Derrida'][np.
↪isfinite(x)])
    c, m, b = popt
    yfit=powerlaw(x,c,m,b)
    truth = (y>1).astype(int) - (y<1).astype(int)
    preds = (yfit>1).astype(int)-(yfit<1).astype(int)
    confusion_matrix = metrics.confusion_matrix(truth,preds)
    cm_display = metrics.ConfusionMatrixDisplay(confusion_matrix =␣
↪confusion_matrix, display_labels = ['Ordered', 'Critical', 'Chaotic'])
    cm_display.plot(ax=ax[1,0])
    cm_display.im_.colorbar.remove()
    mstr='{'+str(np.round(m,2))+'}'
```

```
    label = f'${np.round(c,2)}(\sigma^2 k_e)^{mstr}+{np.round(b,2)}$'
    ax[1,0].set_title(label)

    x=dfN['avgKe']*dfN['avgH']
    y=dfN['Derrida']
    popt, pcov = curve_fit(powerlaw,x[np.isfinite(x)],dfN['Derrida'][np.
↪isfinite(x)])
    c, m, b = popt
    yfit=powerlaw(x,c,m,b)
    truth = (y>1).astype(int) - (y<1).astype(int)
    preds = (yfit>1).astype(int)-(yfit<1).astype(int)
    confusion_matrix = metrics.confusion_matrix(truth,preds)
    cm_display = metrics.ConfusionMatrixDisplay(confusion_matrix =␣
↪confusion_matrix, display_labels = ['Ordered', 'Critical', 'Chaotic'])
    cm_display.plot(ax=ax[1,1])
    cm_display.im_.colorbar.remove()
    mstr='{'+str(np.round(m,2))+'}'
    label = f'${np.round(c,2)}(H k_e)^{mstr}+{np.round(b,2)}$'
    ax[1,1].set_title(label)

    fig.suptitle(f'Criticality predictions ({N=})',fontsize=fs)
    plt.savefig(f'figures/PowerLawConfusion_RBN_{N}.pdf',bbox_inches='tight')
    plt.savefig(f'figures/PowerLawConfusion_RBN_{N}.png',bbox_inches='tight')
    plt.show()
```

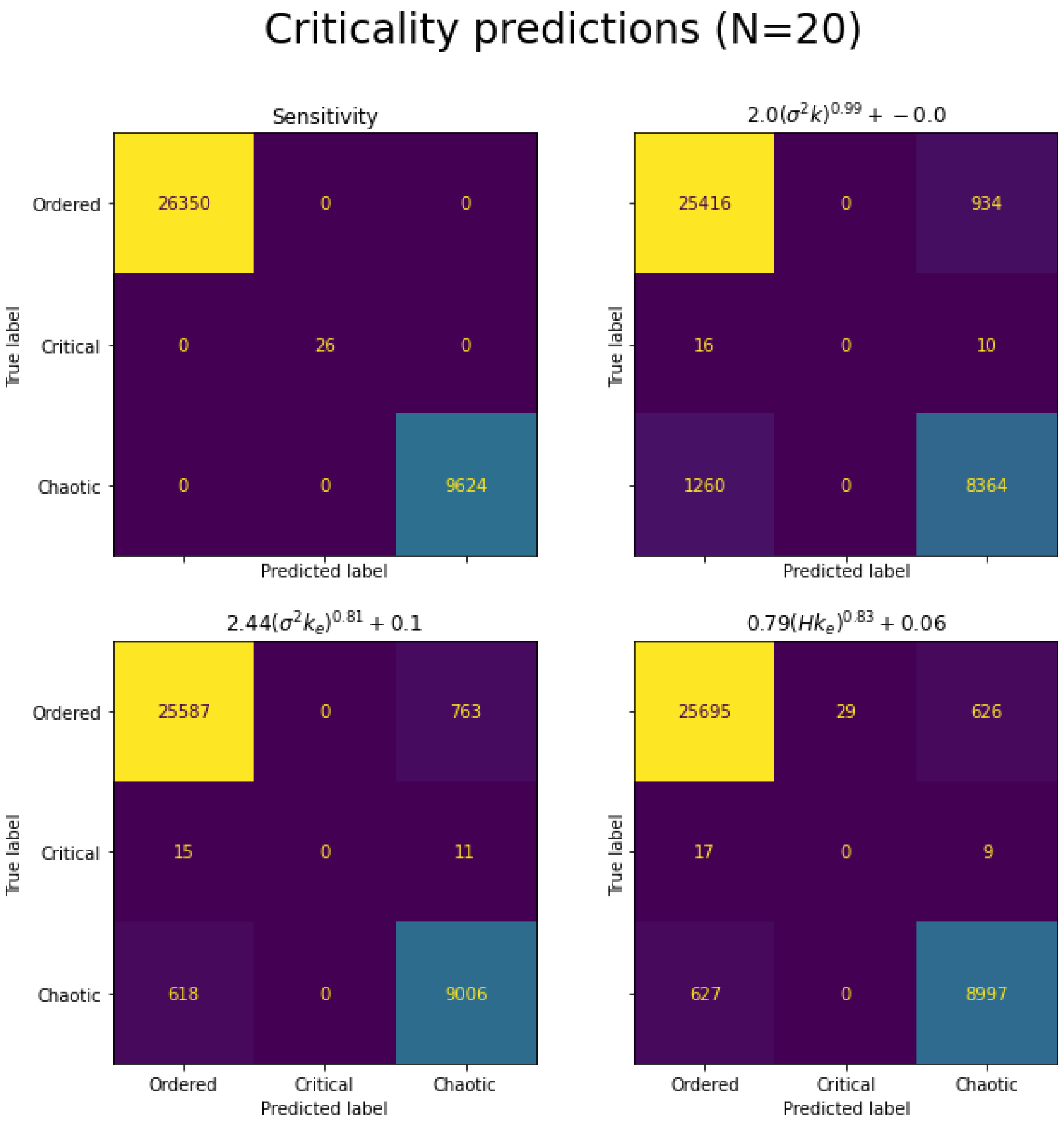
Criticality predictions (N=20)
Sensitivity
$2.0(\sigma^2 k)^{0.99} + -0.0$
$2.44(\sigma^2 k_e)^{0.81} + 0.1$
$0.79(Hk_e)^{0.83} + 0.06$
True label
Predicted label
Ordered
Critical
Chaotic
26350
0
0
0
26
0
0
0
9624
25416
0
934
16
0
10
1260
0
8364
25587
0
763
15
0
11
618
0
9006
25695
29
626
17
0
9
627
0
8997

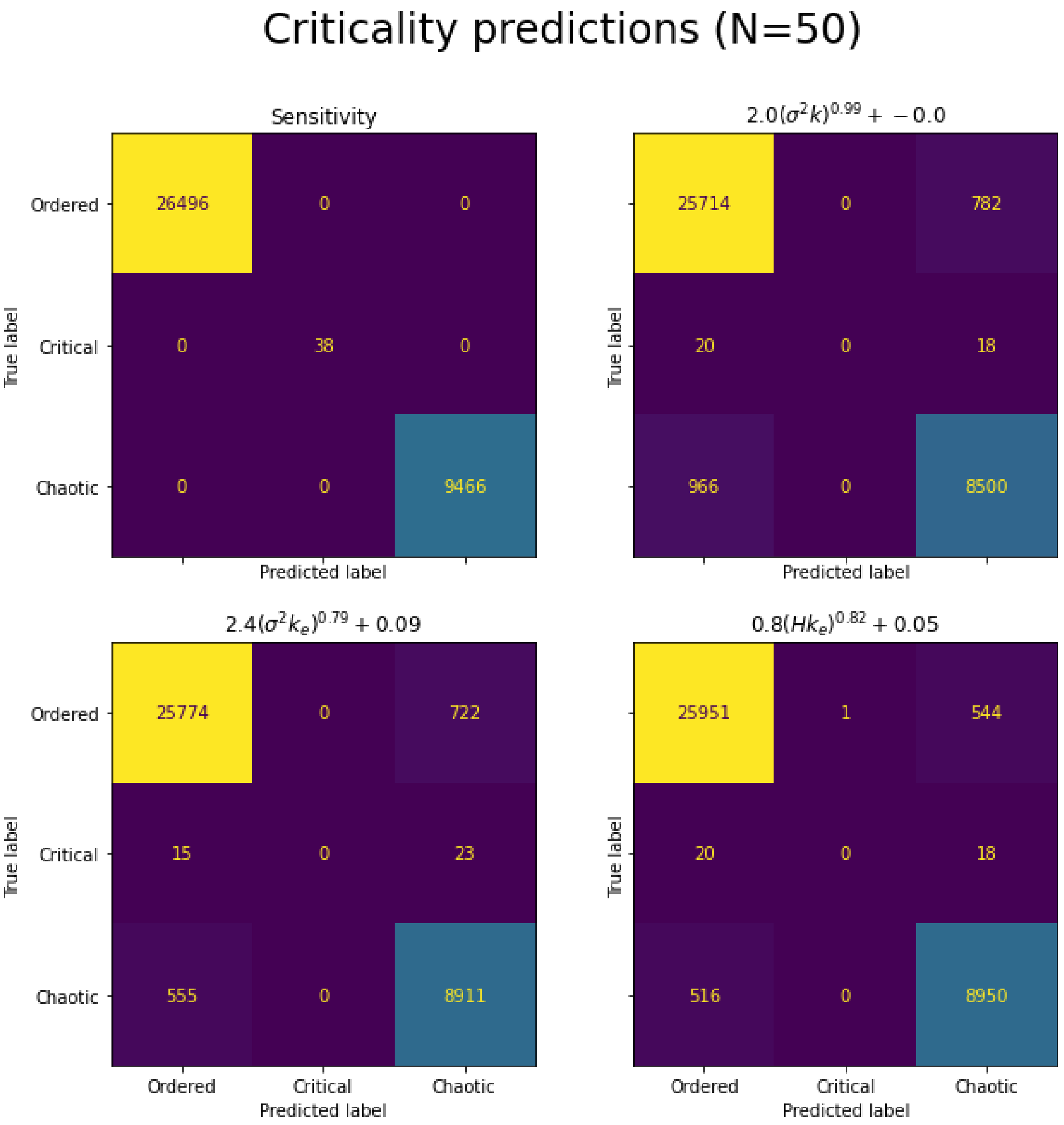
Criticality predictions (N=50)
Sensitivity
$2.0(\sigma^2 k)^{0.99} + -0.0$
$2.4(\sigma^2 k_e)^{0.79} + 0.09$
$0.8(Hk_e)^{0.82} + 0.05$
True label
Predicted label
Ordered
Critical
Chaotic
26496
0
0
0
38
0
0
0
9466
25714
0
782
20
0
18
966
0
8500
25774
0
722
15
0
23
555
0
8911
25951
1
544
20
0
18
516
0
8950

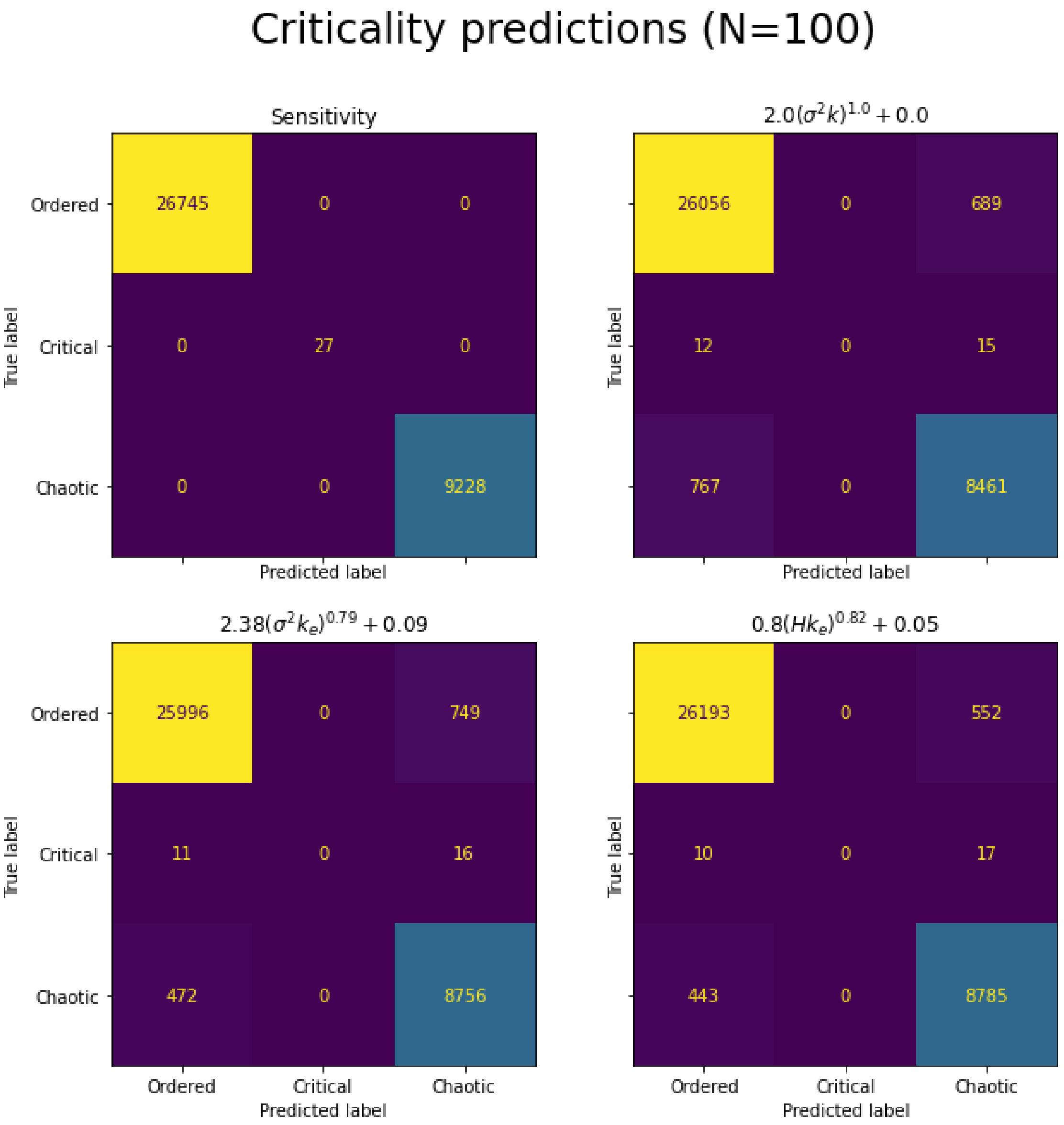

Criticality predictions (N=100)
Sensitivity
$2.0(\sigma^2 k)^{1.0} + 0.0$
$2.38(\sigma^2 k_e)^{0.79} + 0.09$
$0.8(H k_e)^{0.82} + 0.05$
True label
Predicted label
Ordered
Critical
Chaotic
26745
0
0
0
27
0
0
0
9228
26056
0
689
12
0
15
767
0
8461
25996
0
749
11
0
16
472
0
8756
26193
0
552
10
0
17
443
0
8785

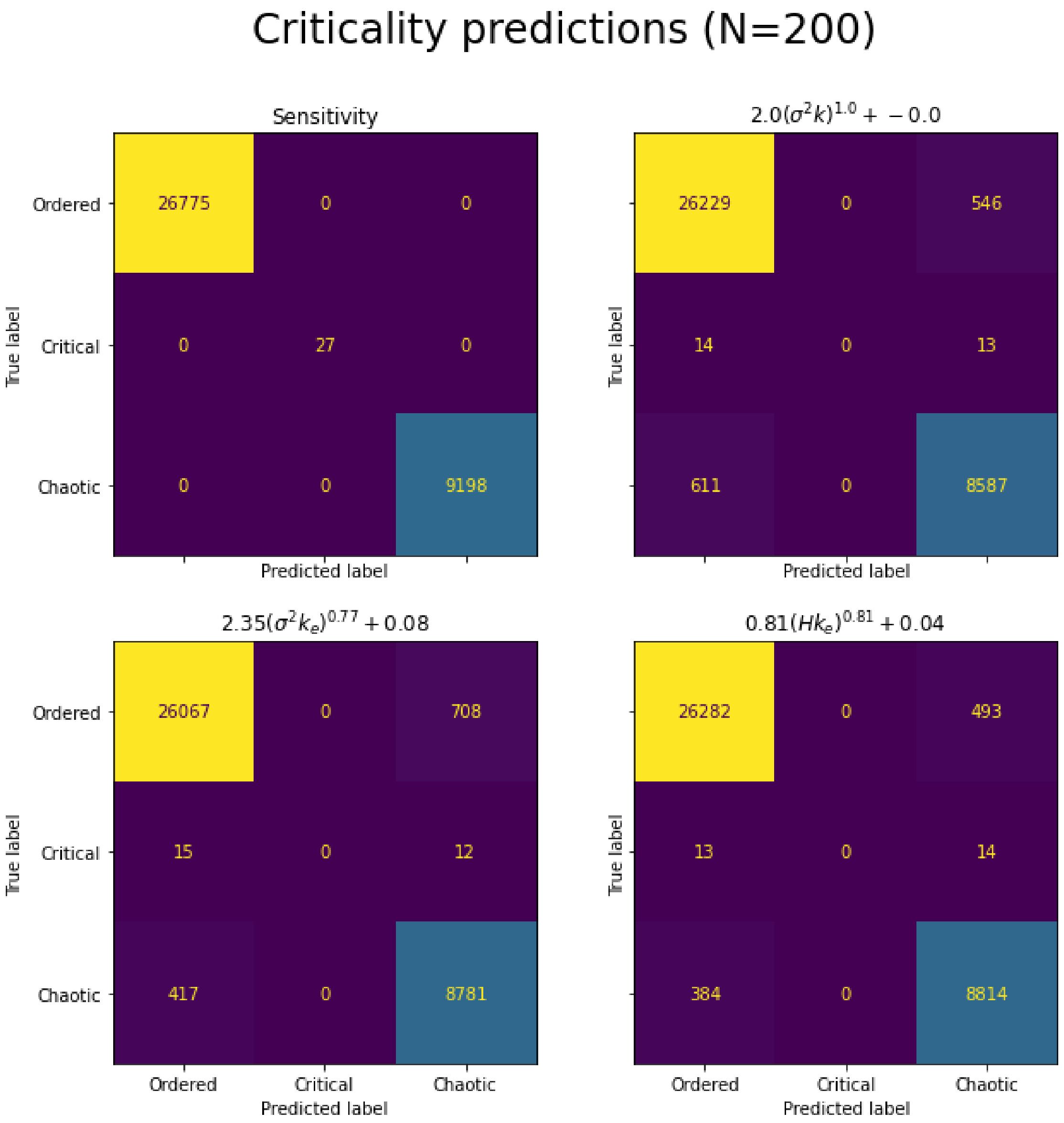

Figure S5: Confusion matrices for the boundary depicted in *Figure S4*. The true label correspond to the Derrida coefficient δ and predicted label to the formula in the figure title relative to the curves in *Figure S4*.

From the diagonal elements in the confusion matrices of Figure S5, we find the percent accuracies of the boundaries to be:

N	20	50	100	200
$\sigma^2 k$	93.83	95.04	95.88	96.71
$\sigma^2 k_e$	96.09	96.35	96.53	96.8
H k_e	96.36	96.95	97.16	97.49

3 Cell Collective Analysis

We now begin analysis of the Cell Collective models. We begin by importing the data and defining the bias entropy and bias variance.

```
[ ]: dfcc = pd.read_csv('cc_network_data.csv')
     dfcc['avgV_est']=dfcc['biasUnweighted']*(1-dfcc['biasUnweighted'])
     dfcc['avgH_est']=-dfcc['biasUnweighted']*np.
      ↪log2(dfcc['biasUnweighted'])-(1-dfcc['biasUnweighted'])*np.
      ↪log2(1-dfcc['biasUnweighted'])
```

We will need a helper function for optimizing the critical boundary.

```
[ ]: def optimize_cut(x,truth,method='Cohen kappa'):
         best_score = 0
         best_cut = 0
         for cut in sorted(x):
             cpreds = (x > cut)
             tp = np.sum(cpreds & truth)
             fp = np.sum(cpreds & ~truth)
             tn = np.sum(~cpreds & ~truth)
             fn = np.sum(~cpreds & truth)

             if method == 'MCC':
                 denom = np.sqrt((tn+fn)*(fp+tp)*(tn+fp)*(fn+tp))
                 if denom == 0:
                     continue
                 score = (tn*tp-fp*fn)/denom

             elif method == 'Accuracy':
                 score = (tp+tn)/(tp+tn+fn+fp)

             elif method == 'Cohen kappa':
                 denom = (tp+fp)*(fp+tn)+(tp+fn)*(fn+tn)
                 if denom == 0:
                     continue
                 score = 2*(tp*tn-fn*fp)/denom
             else:
                 raise ValueError
             if score > best_score:
                 best_score = score
                 best_cut = cut
                 preds = np.copy(cpreds)
         return preds, best_cut, best_score
```

3.1 Summary Statistics of the Cell Collective Networks

[ ]:
```
print('average number of nodes:', dfcc['Nnodes'].mean())
print('Derrida coefficient quartiles:', np.percentile(dfcc['dc'], [0, 25, 50,␣
↪75, 100]))
print('Derrida coefficient mean and standard deviation:', dfcc['dc'].mean(),␣
↪',',  dfcc['dc'].std())
```

```
average number of nodes: 46.78378378378378
Derrida coefficient quartiles: [0.7055    0.9470625 0.9793125 1.0119375 1.249
]
Derrida coefficient mean and standard deviation: 0.9736300675675675 ,
0.08681282869300079
```

We highlight that sensitivity and the Derrida coefficient are in good agreement in these models.

[ ]:
```
fig, ax = plt.subplots(figsize=(10, 10),facecolor='white')
ax.set_facecolor('white')
varx = 's'
vary = 'dc'
cmap = plt.cm.get_cmap('coolwarm')#.reversed()
sc=ax.scatter(dfcc[varx],dfcc[vary],s=100,c=dfcc['dc'],cmap=cmap,
              norm=CenteredNorm(vcenter=1,halfrange=0.5),alpha=1.0)
ax.vlines(1.0,0,2,label='$s=1$',color='k',linestyles='--')
ax.hlines(1.0,0,2,label='$\delta=1$',color='k',linestyles='--')
ax.set_ylim(0.5,1.5)
ax.set_xlim(0.5,1.5)
plt.colorbar(sc)
plt.xlabel('$s$')
plt.ylabel('$\delta$')
plt.title('Derrida coefficient and sensitivity in Cell Collective models')
#plt.legend()
plt.savefig(f'figures/DerridaSensitivity_CC.pdf',bbox_inches='tight')
plt.savefig(f'figures/DerridaSensitivity_CC.png',bbox_inches='tight')
plt.show()
```

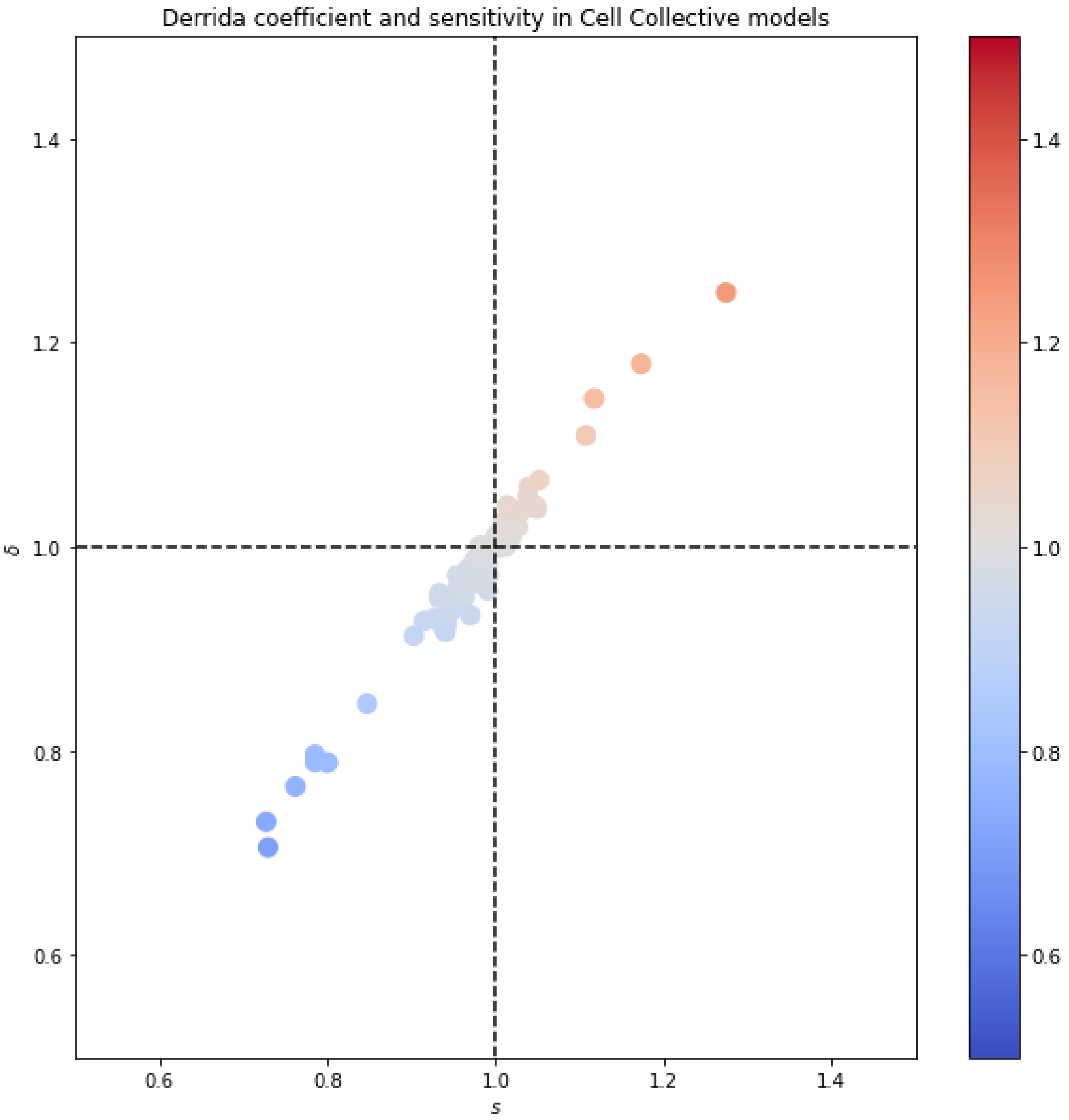

Figure S6: Comparison between average node sensitivity, s, and Derrida coefficient, δ,for the Cell Collective models.

3.2 Cell Collective Analysis Using Naive Bias Averaging

3.2.1 Distribution of Prediction Metrics and Dynamical Regime

We show the dependence of δ and s on the connectivity-spread products considered earlier.

We highlight a critical region between dotted lines. This region is centered on $\delta = 1$ and has width equal to the width of the IQR of the δ distribution.

[ ]:

```
fs = 24
fig, ax = plt.subplots(1,3,figsize=(30, 10),facecolor='white',sharey='row')
cmap = plt.cm.get_cmap('coolwarm')

dd=np.subtract(*np.percentile(dfcc['dc'], [75, 25]))/2
sc=ax[0].
↪scatter(dfcc['avgV_est']*dfcc['k'],dfcc['dc'],s=100,c=dfcc['s'],cmap=cmap,
              norm=CenteredNorm(vcenter=1,halfrange=0.5),alpha=1.0)
ax[0].set_ylim(0.5,1.5)
ax[0].set_xlim(0,1.5)
#ax[0].legend(loc='upper left',fontsize=fs)
ax[0].set_xlabel('$\sigma^2 k$',fontsize=fs)
ax[0].set_ylabel('$\delta$',fontsize=fs)
ax[0].tick_params(axis='both', which='major', labelsize=fs)
ax[0].hlines([1-dd,1+dd],xmin=0,xmax=1.5,colors='k',linestyles='--')

sc=ax[1].
↪scatter(dfcc['avgV_est']*dfcc['ke'],dfcc['dc'],s=100,c=dfcc['s'],cmap=cmap,
              norm=CenteredNorm(vcenter=1,halfrange=0.5),alpha=1.0)
ax[1].set_ylim(0.5,1.5)
ax[1].set_xlim(0,0.5)
#ax[1].legend(loc='upper left',fontsize=fs)
ax[1].set_xlabel('$\sigma^2 k_e$',fontsize=fs)
#ax[1].set_ylabel('$\delta$',fontsize=fs)
ax[1].tick_params(axis='both', which='major', labelsize=fs)
ax[1].hlines([1-dd,1+dd],xmin=0,xmax=0.5,colors='k',linestyles='--')

sc=ax[2].
↪scatter(dfcc['avgH_est']*dfcc['ke'],dfcc['dc'],s=100,c=dfcc['s'],cmap=cmap,
              norm=CenteredNorm(vcenter=1,halfrange=0.5),alpha=1.0)
ax[2].set_ylim(0.5,1.5)
ax[2].set_xlim(0,2)
#ax[2].legend(loc='upper left',fontsize=fs)
ax[2].set_xlabel('$H k_e$',fontsize=fs)
#ax[2].set_ylabel('$\delta$',fontsize=fs)
ax[2].tick_params(axis='both', which='major', labelsize=fs)
ax[2].hlines([1-dd,1+dd],xmin=0,xmax=2,colors='k',linestyles='--')

cbar=fig.colorbar(sc,ax=ax,location='bottom',aspect=75)
cbar.ax.tick_params(axis='both',labelsize=fs)
cbar.set_label('Sensitivity',fontsize=fs)

plt.savefig(f'figures/DerridaConnectivitySpread_CC_from_bias_no_thresh.
↪pdf',bbox_inches='tight')
plt.savefig(f'figures/DerridaConnectivitySpread_CC_from_bias_no_thresh.
↪png',bbox_inches='tight')
plt.show()
```

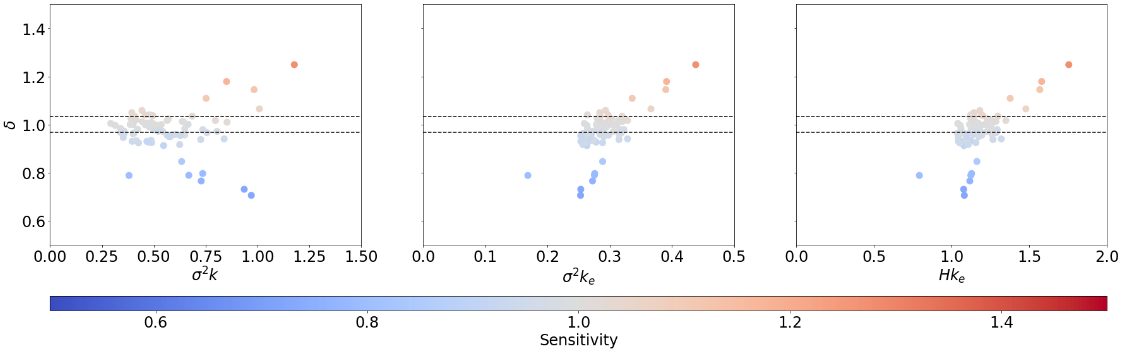

Figure S7: Ability of each measure to predict the Derrida coefficient of the Cell Collective networks. Each point corresponds to a network, and its color indicates the network's average node sensitivity. We plot $\sigma^2 k$ (left), $\sigma^2 k_e$ (center), and Hk_e (right) with respect to the Derrida coefficient δ. The region between the dotted lines indicates a critical region centered at $\delta = 1$ of width equal to the IQR of the δ distribution.

3.2.2 Critical Boundary Prediction Optimization

[ ]:
```
fs = 24
fig, ax = plt.subplots(3,3, figsize=(
        10, 10), sharey='row', sharex='col', facecolor='white')
truth = (dfcc['dc'] > 1)
for row, method in enumerate(['MCC','Accuracy','Cohen kappa']):

    x = dfcc['k']*dfcc['avgV_est']
    preds,cut,score=optimize_cut(x,truth,method=method)
    confusion_matrix = metrics.confusion_matrix(truth, preds)
    cm_display = metrics.ConfusionMatrixDisplay(
        confusion_matrix=confusion_matrix, display_labels=['$\delta \leq 1$',␣
 ↪'$\delta > 1$'])
    cm_display.plot(ax=ax[row,0])
    cm_display.im_.colorbar.remove()
    #mstr = '{'+str(np.round(m, 2))+'}'
    label = f'$\sigma^2 k$={np.round(cut,3)}, {method}={np.round(score,2)}'
    ax[row,0].set_title(label)

    x = dfcc['ke']*dfcc['avgV_est']
    preds,cut,score=optimize_cut(x,truth,method=method)
    confusion_matrix = metrics.confusion_matrix(truth, preds)
    cm_display = metrics.ConfusionMatrixDisplay(
        confusion_matrix=confusion_matrix, display_labels=['$\delta \leq 1$',␣
 ↪'$\delta > 1$'])
    cm_display.plot(ax=ax[row,1])
    cm_display.im_.colorbar.remove()
```

```
    #mstr = '{'+str(np.round(m, 2))+'}'
    label = f'$\sigma^2 k_e$={np.round(cut,3)}, {method}={np.round(score,2)}'
    ax[row,1].set_title(label)

    x = dfcc['ke']*dfcc['avgH_est']
    preds,cut,score=optimize_cut(x,truth,method=method)
    confusion_matrix = metrics.confusion_matrix(truth, preds)
    cm_display = metrics.ConfusionMatrixDisplay(
        confusion_matrix=confusion_matrix, display_labels=['$\delta \leq 1$',␣
↪'$\delta > 1$'])
    cm_display.plot(ax=ax[row,2])
    cm_display.im_.colorbar.remove()
    #mstr = '{'+str(np.round(m, 2))+'}'
    label = f'$H k_e$={np.round(cut,3)}, {method}={np.round(score,2)}'
    ax[row,2].set_title(label)


fig.tight_layout()
plt.savefig(f'figures/ConfusionOptimized_CC_from_bias.png',bbox_inches='tight')
plt.savefig(f'figures/ConfusionOptimized_CC_from_bias.pdf',bbox_inches='tight')
plt.show()
```

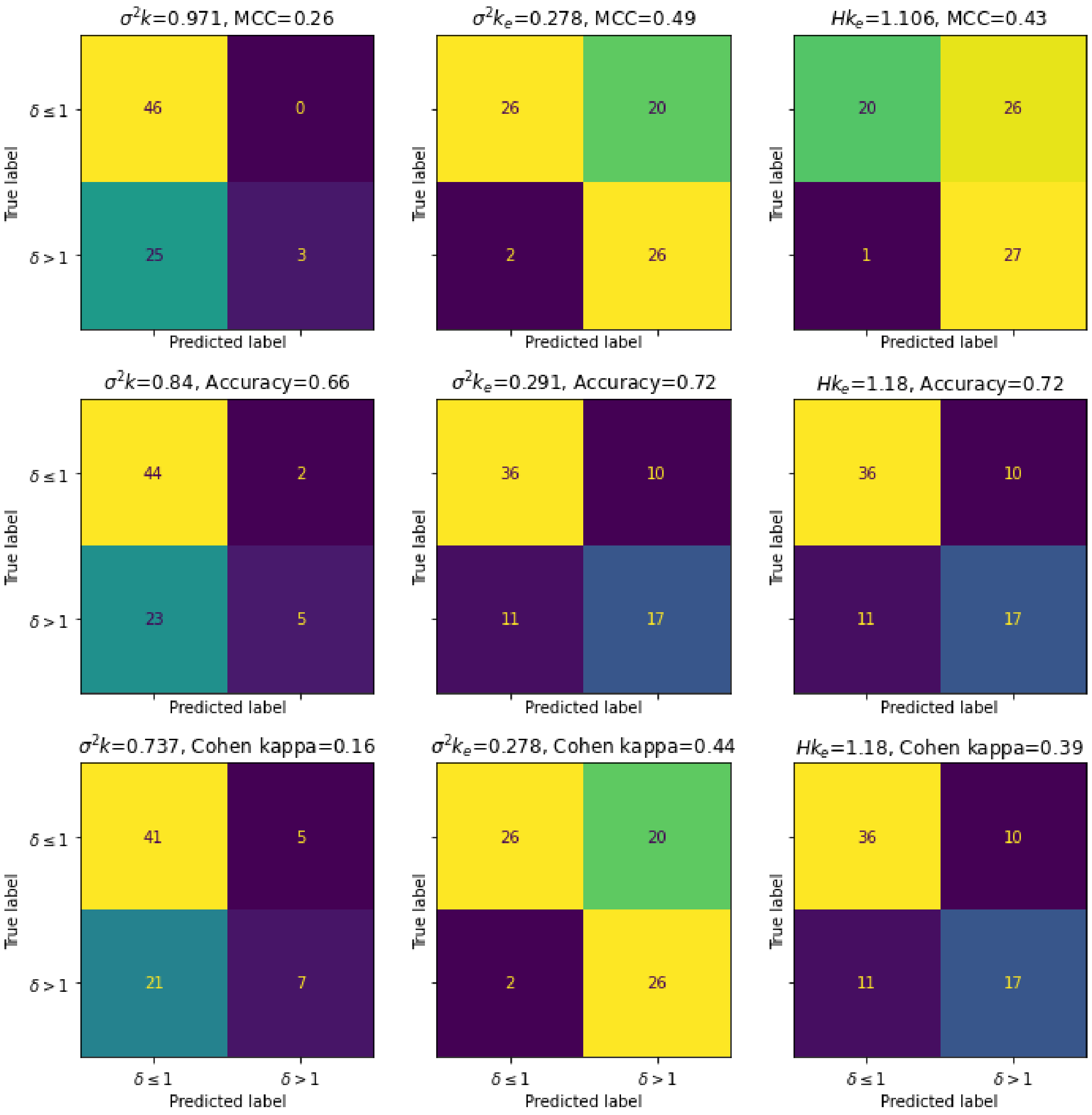

Figure S8: Confusion matrices for the optimal critical boundaries in the Cell Collective computed using variance and entropy of the average bias. Each boundary was optimized to maximize the Matthews correlation coefficient (MCC), the accuracy, and the Cohen kappa metric. Each matrix corresponds to a given threshold parameter that is evaluated to predict the dynamical regime. From left to right, these are $\sigma^2 k$, $\sigma^2 k_e$, and Hk_e. The predicted regime is given by the horizontal labels, and the ground truth regime, as computed from the Derrida coefficient, is given by the vertical labels.

We will record the values that give rise to the greatest accuracy.

[ ]:
```
acut_KV_fb = 0.84
acut_KeV_fb = 0.291
acut_KeH_fb = 1.18
```

3.3 Comparison of Optimized Boundaries to RBN Boundary

[ ]:
```
fig, ax = plt.subplots(1,3,figsize=(30, 10),facecolor='white')
bounds = ([0,0,-10],[10,10,10]) #c,m,b
fs = 24
cmap = plt.cm.get_cmap('coolwarm')

varx = 'avgV_est'
vary = 'k'

sc=ax[0].scatter(dfcc[varx],dfcc[vary],s=100,c=dfcc['dc'],cmap=cmap,
              norm=CenteredNorm(vcenter=1,halfrange=0.5),alpha=1.0)
px = np.arange(0.01,1,0.01)
py=acut_KV_fb/px
py2=critical_kappa(px,2,1,0)
ax[0].plot(px,py2,'--k',label='Fit from RBNs (N=50)')
ax[0].plot(px,py,'-k',label='Most accurate for CC')
ax[0].set_ylim(1,7)
ax[0].set_xlim(0.125,0.25)
ax[0].legend(loc='upper left',fontsize=fs)
ax[0].set_xlabel('$\sigma^2$',fontsize=fs)
ax[0].set_ylabel('$k$',fontsize=fs)
ax[0].tick_params(axis='both', which='major', labelsize=fs)
ax[0].set_title('$\sigma^2 k$ vs $\delta$ critical boundary',fontsize=fs)

varx = 'avgV_est'
vary = 'ke'

sc=ax[1].scatter(dfcc[varx],dfcc[vary],s=100,c=dfcc['dc'],cmap=cmap,
              norm=CenteredNorm(vcenter=1,halfrange=0.5),alpha=1.0)
px = np.arange(0.01,1,0.01)
py=acut_KeV_fb/px
py2=critical_kappa(px,2.4,0.8,0.09)
ax[1].plot(px,py2,'--k',label='Fit from RBNs (N=50)')
ax[1].plot(px,py,'-k',label='Most accurate for CC')
ax[1].set_ylim(1,2)
ax[1].set_xlim(0.125,0.25)
ax[1].legend(loc='upper left',fontsize=fs)
ax[1].set_xlabel('$\sigma^2$',fontsize=fs)
ax[1].set_ylabel('$k_e$',fontsize=fs)
ax[1].tick_params(axis='both', which='major', labelsize=fs)
ax[1].set_title('$\sigma^2 k_e$ vs $\delta$ critical boundary',fontsize=fs)

varx = 'avgH_est'
vary = 'ke'

sc=ax[2].scatter(dfcc[varx],dfcc[vary],s=100,c=dfcc['dc'],cmap=cmap,
```

```
                norm=CenteredNorm(vcenter=1,halfrange=0.5),alpha=1.0,label=None)
px = np.arange(0.01,1.01,0.01)
py=acut_KeH_fb/px
py2=critical_kappa(px,0.8,0.83,0.05)
ax[2].plot(px,py2,'--k',label='Fit from RBNs (N=50)')
ax[2].plot(px,py,'-k',label='Most accurate for CC')
ax[2].set_ylim(1,2)
ax[2].set_xlim(0.5,1)
ax[2].legend(loc='upper left',fontsize=fs)
ax[2].set_xlabel('$H$',fontsize=fs)
ax[2].set_ylabel('$k_e$',fontsize=fs)
ax[2].tick_params(axis='both', which='major', labelsize=fs)
ax[2].set_title('$H k_e$ vs $\delta$ critical boundary',fontsize=fs)

cbar=fig.colorbar(sc,ax=ax,location='bottom',aspect=75)
cbar.ax.tick_params(axis='both',labelsize=fs)
cbar.set_label('Derrida Coefficient',fontsize=fs)

plt.savefig(f'figures/PowerLawFits_CC_from_bias.pdf',bbox_inches='tight')
plt.savefig(f'figures/PowerLawFits_CC_from_bias.png',bbox_inches='tight')
plt.show()
```

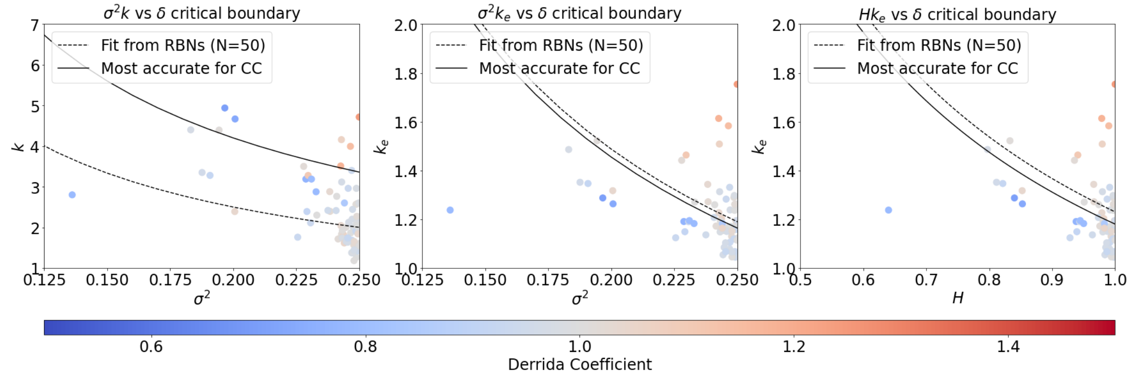

Figure S9: Critical boundaries obtained from RBNs fit of with $N = 50$, average number of nodes in CC, compared to the relation between the measures found from the binary classification.

3.4 Cell Collective Analysis Using Improved Bias Averaging

We now consider an alternate averaging scheme for the Cell Collective models. The theory of RBNs considers σ^2 as computed from the overall bias of the network, rather than computed from the average of each node's output variance. This traditional approach gives rise to the classical results in the theory. However, the second, less-convential approach we propose here performs better in the Cell Collective (especially when paired with effective connectivity), as we will demonstrate. We use the prime to denote that the parameters are computed using this alternate averaging scheme.

3.4.1 Distribution of Prediction Metrics and Dynamical Regime

[ ]:
```
fs = 36
fsa = 24
fig, ax = plt.subplots(1,3,figsize=(30, 10),facecolor='white',sharey='row')
cmap = plt.cm.get_cmap('coolwarm')

dd=np.subtract(*np.percentile(dfcc['dc'], [75, 25]))/2
sc=ax[0].scatter(dfcc['avgV']*dfcc['k'],dfcc['dc'],s=100,c=dfcc['s'],cmap=cmap,
              norm=CenteredNorm(vcenter=1,halfrange=0.5),alpha=1.0)
ax[0].set_ylim(0.5,1.5)
ax[0].set_xlim(0,1.5)
ax[0].set_xlabel('$(\sigma^2)^\prime k$',fontsize=fs)
ax[0].set_ylabel('$\delta$',fontsize=fs)
ax[0].tick_params(axis='both', which='major', labelsize=fsa)
ax[0].hlines([1-dd,1+dd],xmin=0,xmax=1.5,colors='k',linestyles='--')

sc=ax[1].scatter(dfcc['avgV']*dfcc['ke'],dfcc['dc'],s=100,c=dfcc['s'],cmap=cmap,
              norm=CenteredNorm(vcenter=1,halfrange=0.5),alpha=1.0)
ax[1].set_ylim(0.5,1.5)
ax[1].set_xlim(0,0.5)
ax[1].set_xlabel('$(\sigma^2)^\prime k_e$',fontsize=fs)
ax[1].tick_params(axis='both', which='major', labelsize=fsa)
ax[1].hlines([1-dd,1+dd],xmin=0,xmax=0.5,colors='k',linestyles='--')

sc=ax[2].scatter(dfcc['avgH']*dfcc['ke'],dfcc['dc'],s=100,c=dfcc['s'],cmap=cmap,
              norm=CenteredNorm(vcenter=1,halfrange=0.5),alpha=1.0)
ax[2].set_ylim(0.5,1.5)
ax[2].set_xlim(0,2)
ax[2].set_xlabel('$H^\prime k_e$',fontsize=fs)
ax[2].tick_params(axis='both', which='major', labelsize=fsa)
ax[2].hlines([1-dd,1+dd],xmin=0,xmax=2,colors='k',linestyles='--')

cbar=fig.colorbar(sc,ax=ax,location='bottom',aspect=75)
cbar.ax.tick_params(axis='both',labelsize=fsa)
cbar.set_label('Sensitivity',fontsize=fs)

plt.savefig(f'figures/DerridaConnectivitySpread_CC.pdf',bbox_inches='tight')
plt.savefig(f'figures/DerridaConnectivitySpread_CC.png',bbox_inches='tight')
plt.show()
```

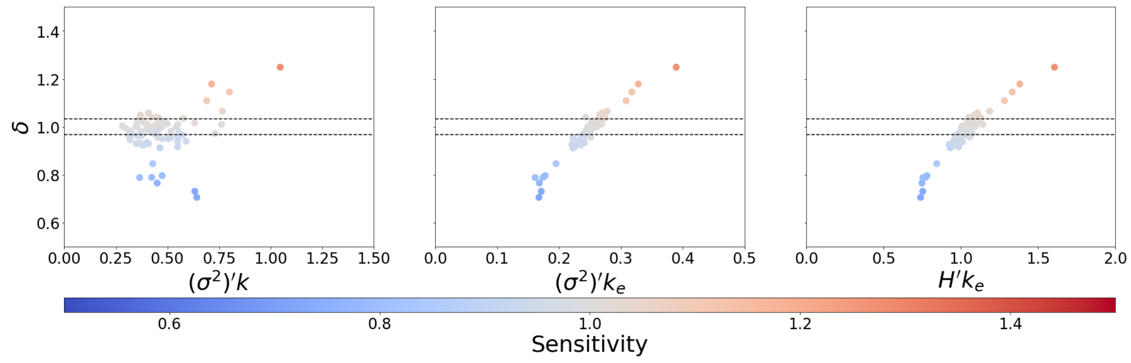

Figure S10: Same as Figure 4, but without classification boundary lines.

The first indication that the alternate averaging scheme is more appropriate in this setting is the qualitatively improved correlation relative to the previously explored averaging scheme.

3.4.2 Critical Boundary Prediction Optimization

[ ]:
```
fs = 24
mpl.rcParams["font.size"] = 20
tfs = 12
fig, ax = plt.subplots(3,3, figsize=(
        10, 10), sharey='row', sharex='col', facecolor='white')
truth = (dfcc['dc'] > 1)
for row, method in enumerate(['MCC','Accuracy','Cohen kappa']):

    x = dfcc['k']*dfcc['avgV']
    preds,cut,score=optimize_cut(x,truth,method=method)
    confusion_matrix = metrics.confusion_matrix(truth, preds)
    cm_display = metrics.ConfusionMatrixDisplay(
        confusion_matrix=confusion_matrix, display_labels=['$\delta \leq 1$',␣
↪'$\delta > 1$'])
    cm_display.plot(ax=ax[row,0])
    cm_display.im_.colorbar.remove()
    label = f'$(\sigma^2)^\prime k$={np.round(cut,3)}, {method}={np.
↪round(score,2)}'
    ax[row,0].set_title(label, fontsize=tfs)

    x = dfcc['ke']*dfcc['avgV']
    preds,cut,score=optimize_cut(x,truth,method=method)
    confusion_matrix = metrics.confusion_matrix(truth, preds)
    cm_display = metrics.ConfusionMatrixDisplay(
        confusion_matrix=confusion_matrix, display_labels=['$\delta \leq 1$',␣
↪'$\delta > 1$'])
    cm_display.plot(ax=ax[row,1])
    cm_display.im_.colorbar.remove()
```

```
    label = f'$(\sigma^2)^\prime k_e$={np.round(cut,3)}, {method}={np.
↪round(score,2)}'
    ax[row,1].set_title(label, fontsize=tfs)

    x = dfcc['ke']*dfcc['avgH']
    preds,cut,score=optimize_cut(x,truth,method=method)
    confusion_matrix = metrics.confusion_matrix(truth, preds)
    cm_display = metrics.ConfusionMatrixDisplay(
        confusion_matrix=confusion_matrix, display_labels=['$\delta \leq 1$',␣
↪'$\delta > 1$'])
    cm_display.plot(ax=ax[row,2])
    cm_display.im_.colorbar.remove()
    label = f'$H^\prime k_e$={np.round(cut,3)}, {method}={np.round(score,2)}'
    ax[row,2].set_title(label, fontsize=tfs)


fig.tight_layout()
plt.savefig(f'figures/ConfusionOptimized_CC_all.png',bbox_inches='tight')
plt.savefig(f'figures/ConfusionOptimized_CC_all.pdf',bbox_inches='tight')
plt.show()
```

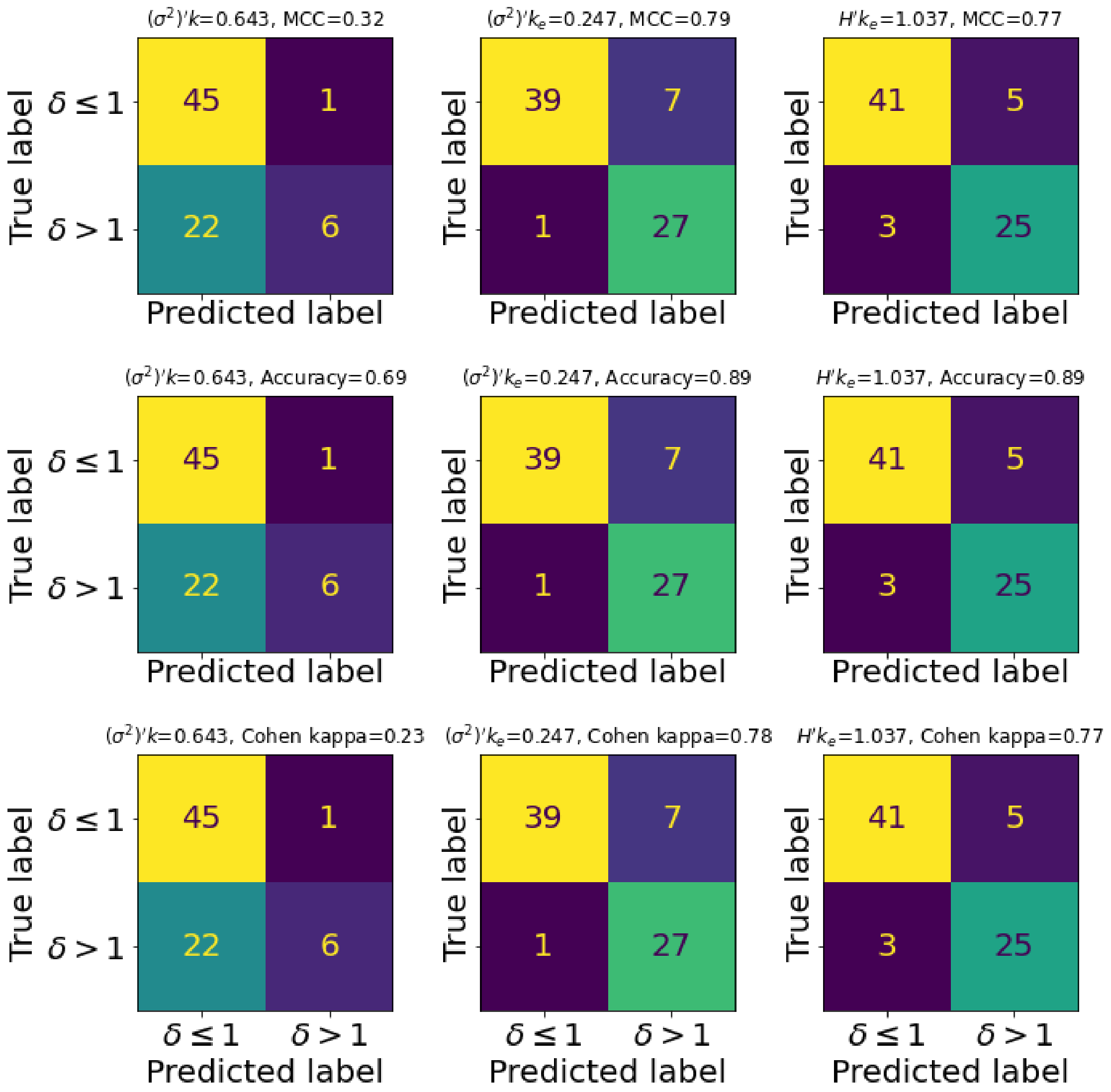

Figure S11: Confusion matrices for the optimal critical boundaries in the Cell Collective computed using average node activation spread measures. Each boundary was optimized to maximize the Matthews correlation coefficient (MCC), the accuracy, and the Cohen kappa metric. Each matrix corresponds to a given threshold parameter that is evaluated to predict the dynamical regime. From left to right, these are $(\sigma^2)'k$, $(\sigma^2)'k_e$, and $H'k_e$.

We record the most accurate thresholds (which also optimize all other metrics considered, in this case).

[ ]:
```
acut_KV = 0.643
acut_KeV = 0.247
acut_KeH = 1.037
```

Because these metrics are all optimized by the same boundary, we can condense the above figure for more convenient presentation.

[ ]:
```
fs = 14
mpl.rcParams["font.size"] = 20
fig, ax = plt.subplots(1,3, figsize=(
        10, 10), sharey='row', sharex='col', facecolor='white')
truth = (dfcc['dc'] > 1)

x = dfcc['k']*dfcc['avgV']
preds,cut,score=optimize_cut(x,truth,method='Accuracy')
confusion_matrix = metrics.confusion_matrix(truth, preds)
cm_display = metrics.ConfusionMatrixDisplay(
    confusion_matrix=confusion_matrix, display_labels=[ f'$(\sigma^2)^\prime␣
↪k\leq ${np.round(cut,2)}', f'$(\sigma^2)^\prime k >${np.
↪round(cut,2)}'])#['$\delta \leq 1$', '$\delta > 1$'])
cm_display.plot(ax=ax[0])
cm_display.im_.colorbar.remove()
mstr = '{'+str(np.round(m, 2))+'}'
label = f'$(\sigma^2)^\prime k$={np.round(cut,3)}'
#ax[0].set_title(label,fontsize=fs)

x = dfcc['ke']*dfcc['avgV']
preds,cut,score=optimize_cut(x,truth,method='Accuracy')
confusion_matrix = metrics.confusion_matrix(truth, preds)
cm_display = metrics.ConfusionMatrixDisplay(
    confusion_matrix=confusion_matrix, display_labels=[ f'$(\sigma^2)^\prime␣
↪k_e\leq ${np.round(cut,2)}', f'$(\sigma^2)^\prime k_e >${np.
↪round(cut,2)}'])#['$\delta \leq 1$', '$\delta > 1$'])['$\delta \leq 1$',␣
↪'$\delta > 1$'],)
cm_display.plot(ax=ax[1])
cm_display.im_.colorbar.remove()
mstr = '{'+str(np.round(m, 2))+'}'
label = f'$(\sigma^2)^\prime k_e$={np.round(cut,3)}'
#ax[1].set_title(label,fontsize=fs)

x = dfcc['ke']*dfcc['avgH']
preds,cut,score=optimize_cut(x,truth,method='Accuracy')
confusion_matrix = metrics.confusion_matrix(truth, preds)
cm_display = metrics.ConfusionMatrixDisplay(
    confusion_matrix=confusion_matrix, display_labels=[ f'$H^\prime k_e\leq␣
↪${np.round(cut,2)}', f'$H^\prime k_e >${np.round(cut,2)}'])#['$\delta \leq␣
↪1$', '$\delta > 1$'])['$\delta \leq 1$', '$\delta > 1$'])
cm_display.plot(ax=ax[2])
cm_display.im_.colorbar.remove()
mstr = '{'+str(np.round(m, 2))+'}'
label = f'$H^\prime k_e$={np.round(cut,3)}'
```

```
#ax[2].set_title(label,fontsize=fs)


# fix font sizes
for axis in ax:
    axis.tick_params(axis='both', which='major', labelsize=fs)
    axis.set_xlabel('');
    axis.set_ylabel('');
    #axis.xaxis.set_ticklabels(['Below', 'Above']);
    axis.yaxis.set_ticklabels(['$\delta \leq 1$', '$\delta > 1$']);

fig.suptitle(f'Optimal criticality predictions in the Cell␣
 ↪Collective',fontsize=fs)
fig.tight_layout()
fig.subplots_adjust(top=1.6)
plt.savefig(f'figures/ConfusionOptimized_CC.png',bbox_inches='tight')
plt.savefig(f'figures/ConfusionOptimized_CC.pdf',bbox_inches='tight')
plt.show()
```

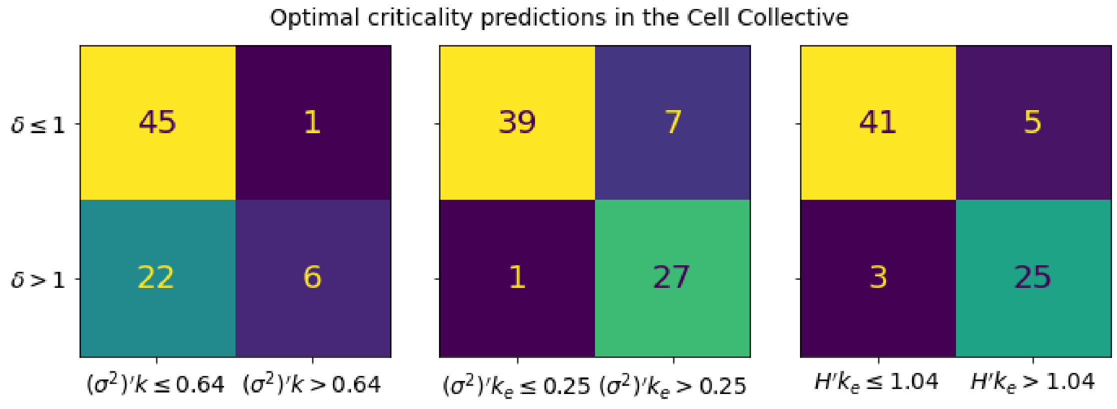

Figure S12: Same as Figure 5

3.4.3 Optimized Critical Boundary Plots

We now plot the critical boundaries we have obtained via the optimization above.

[ ]:
```
fig, ax = plt.subplots(1,3,figsize=(30, 10),facecolor='white')
bounds = ([0,0,-10],[10,10,10]) #c,m,b
fs = 36
fsa = 24
cmap = plt.cm.get_cmap('coolwarm')

varx = 'avgV'
vary = 'k'
```

```
sc=ax[0].scatter(dfcc[varx],dfcc[vary],s=100,c=dfcc['dc'],cmap=cmap,
              norm=CenteredNorm(vcenter=1,halfrange=0.5),alpha=1.0)
px = np.arange(0.01,1,0.01)
py=acut_KV/px
ax[0].plot(px,py,'-k',label='Most accurate for CC')
ax[0].set_ylim(1,7)
ax[0].set_xlim(0.125,0.25)
#ax[0].legend(loc='upper left',fontsize=fs)
ax[0].set_xlabel('$(\sigma^2)^\prime$',fontsize=fs)
ax[0].set_ylabel('$k$',fontsize=fs)
ax[0].tick_params(axis='both', which='major', labelsize=fsa)
ax[0].set_title('$(\sigma^2)^\prime k$ vs $\delta$ critical␣
 ↪boundary',fontsize=fs)

varx = 'avgV'
vary = 'ke'

sc=ax[1].scatter(dfcc[varx],dfcc[vary],s=100,c=dfcc['dc'],cmap=cmap,
              norm=CenteredNorm(vcenter=1,halfrange=0.5),alpha=1.0)
px = np.arange(0.01,1,0.01)
py=acut_KeV/px
ax[1].plot(px,py,'-k',label='Most accurate for CC')
ax[1].set_ylim(1,2)
ax[1].set_xlim(0.125,0.25)
#ax[1].legend(loc='upper left',fontsize=fs)
ax[1].set_xlabel('$(\sigma^2)^\prime$',fontsize=fs)
ax[1].set_ylabel('$k_e$',fontsize=fs)
ax[1].tick_params(axis='both', which='major', labelsize=fsa)
ax[1].set_title('$(\sigma^2)^\prime k_e$ vs $\delta$ critical␣
 ↪boundary',fontsize=fs)

varx = 'avgH'
vary = 'ke'

sc=ax[2].scatter(dfcc[varx],dfcc[vary],s=100,c=dfcc['dc'],cmap=cmap,
              norm=CenteredNorm(vcenter=1,halfrange=0.5),alpha=1.0,label=None)
px = np.arange(0.01,1.01,0.01)
py=acut_KeH/px
ax[2].plot(px,py,'-k',label='Most accurate for CC')
ax[2].set_ylim(1,2)
ax[2].set_xlim(0.5,1)
#ax[2].legend(loc='upper left',fontsize=fs)
ax[2].set_xlabel('$H^\prime$',fontsize=fs)
ax[2].set_ylabel('$k_e$',fontsize=fs)
ax[2].tick_params(axis='both', which='major', labelsize=fsa)
ax[2].set_title('$H^\prime k_e$ vs $\delta$ critical boundary',fontsize=fs)
```

```
cbar=fig.colorbar(sc,ax=ax,location='bottom',aspect=75)
cbar.ax.tick_params(axis='both',labelsize=fs)
cbar.set_label('Derrida Coefficient',fontsize=fs)

plt.savefig(f'figures/PowerLawFits_CC.pdf',bbox_inches='tight')
plt.savefig(f'figures/PowerLawFits_CC.png',bbox_inches='tight')
plt.show()
```

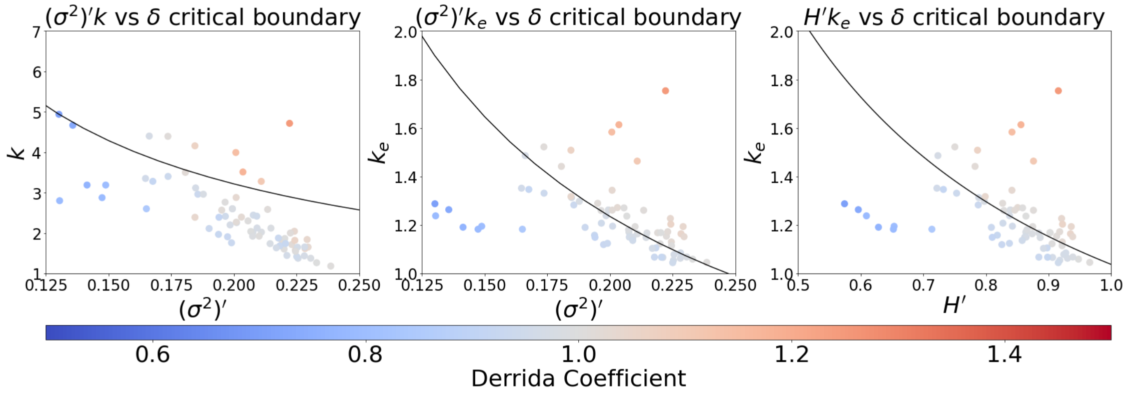

Figure S13: Same as Figure 6 - NOTE: Separation between dashed and solid line not clear, is it one on top of the other?

3.4.4 Critical Region Prediction

The critical region here is taken to be the range of δ values (predicted by the connectivity-spread products or measured) that is centered at $\delta = 1$ and has width equal to the IQR of the δ distribution (predicted or measured). We use the optimal boundaries obtained earlier.

[ ]:
```
from sklearn.metrics import matthews_corrcoef
fs = 20
mpl.rcParams["font.size"] = 20
dev_frac = 2
critical_range = np.subtract(*np.percentile(dfcc['dc'], [75, 25]))/
↪dev_frac#dfcc['dc'].std()/dev_frac
dd = critical_range
truth = (dfcc['dc'] > (1+critical_range)).astype(int) - \
    (dfcc['dc'] < (1-critical_range)).astype(int)

fig, ax = plt.subplots(1, 3, figsize=(
    15, 10), sharey='row', sharex='col', facecolor='white')

x = dfcc['k']*dfcc['avgV']
yfit = x/acut_KV
```

```
critical_range = np.subtract(*np.percentile(yfit, [75, 25]))/dev_frac#yfit.
 ↪std()/dev_frac

best_acc = -1
best_range = 0
for yref in sorted(yfit,key=lambda x: np.abs(1-x)):
    critical_range = np.abs(1-yref)
    preds_test = (yfit > (1+critical_range)).astype(int) - \
        (yfit < (1-critical_range)).astype(int)
    acc = np.sum(preds_test==truth)
    if acc >= best_acc:
        best_acc = acc
        preds = preds_test[:]
        best_range = critical_range
cr_KV = best_range*acut_KV
confusion_matrix = metrics.confusion_matrix(truth, preds)
cm_display = metrics.ConfusionMatrixDisplay(
    confusion_matrix=confusion_matrix, display_labels=['Ordered', 'Critical',␣
 ↪'Chaotic'])
cm_display.plot(ax=ax[0])
cm_display.im_.colorbar.remove()
label = f'$(\sigma^2)^\prime k$ boundary, fit from CC'
ax[0].set_title(f'Region Width = {np.round(2*cr_KV,2)}',fontsize=fs)
ax[0].set_xlabel(f'Dynamical regime from $(\sigma^2)^\prime k$',fontsize=fs)


x = dfcc['ke']*dfcc['avgV']
yfit = x/acut_KeV
critical_range = np.subtract(*np.percentile(yfit, [75, 25]))/dev_frac#yfit.
 ↪std()/dev_frac
preds = (yfit > (1+critical_range)).astype(int) - \
    (yfit < (1-critical_range)).astype(int)

best_acc = -1
best_range = 0
for yref in sorted(yfit,key=lambda x: np.abs(1-x)):
    critical_range = np.abs(1-yref)
    preds_test = (yfit > (1+critical_range)).astype(int) - \
        (yfit < (1-critical_range)).astype(int)
    acc = np.sum(preds_test==truth)
    if acc >= best_acc:
        best_acc = acc
        preds = preds_test[:]
        best_range = critical_range
cr_KeV = best_range*acut_KeV
confusion_matrix = metrics.confusion_matrix(truth, preds)
```

```
cm_display = metrics.ConfusionMatrixDisplay(
    confusion_matrix=confusion_matrix, display_labels=['Ordered', 'Critical', 'Chaotic'])
cm_display.plot(ax=ax[1])
cm_display.im_.colorbar.remove()
label = f'$(\sigma^2)^\prime k_e$ boundary, fit from CC'
ax[1].set_title(f'Region Width = {np.round(2*cr_KeV,2)}',fontsize=fs)
ax[1].set_xlabel(f'Dynamical regime from $(\sigma^2)^\prime k_e$',fontsize=fs)

x = dfcc['ke']*dfcc['avgH']
yfit = x/acut_KeH
critical_range = np.subtract(*np.percentile(yfit, [75, 25]))/dev_frac#yfit.std()/dev_frac
preds = (yfit > (1+critical_range)).astype(int) - \
    (yfit < (1-critical_range)).astype(int)

best_acc = -1
best_range = 0
for yref in sorted(yfit,key=lambda x: np.abs(1-x)):
    critical_range = np.abs(1-yref)
    preds_test = (yfit > (1+critical_range)).astype(int) - \
        (yfit < (1-critical_range)).astype(int)
    acc = np.sum(preds_test==truth)
    if acc >= best_acc:
        best_acc = acc
        preds = preds_test[:]
        best_range = critical_range
cr_KeH = best_range*acut_KeH
confusion_matrix = metrics.confusion_matrix(truth, preds)
cm_display = metrics.ConfusionMatrixDisplay(
    confusion_matrix=confusion_matrix, display_labels=['Ordered', 'Critical', 'Chaotic'])
cm_display.plot(ax=ax[2])
cm_display.im_.colorbar.remove()
label = f'$H^\prime k_e$ boundary, fit from CC'
ax[2].set_title(f'Region Width = {np.round(2*cr_KeH,2)}',fontsize=fs)
ax[2].set_xlabel(f'Dynamical regime from $H^\prime k_e$',fontsize=fs)

# fix font sizes
for axis in ax:
    axis.tick_params(axis='both', which='major', labelsize=fs)
    #axis.set_xlabel('');
    axis.set_ylabel('');
    #axis.xaxis.set_ticklabels(['Below', 'Above']);
    axis.yaxis.set_ticklabels(['Ordered', 'Critical', 'Chaotic']);
ax[0].set_ylabel(f'Dynamical regime from $\delta$\nRegion Width = {np.round(2*dd,2)}',fontsize=fs)
```

```
fig.suptitle(f'Critical interval predictions in the Cell␣
↪Collective',fontsize=fs)
fig.tight_layout()
fig.subplots_adjust(top=1.4)

plt.savefig(f'figures/ConfusionIQR_CC.pdf',bbox_inches='tight')
plt.savefig(f'figures/ConfusionIQR_CC.png',bbox_inches='tight')
plt.show()
```

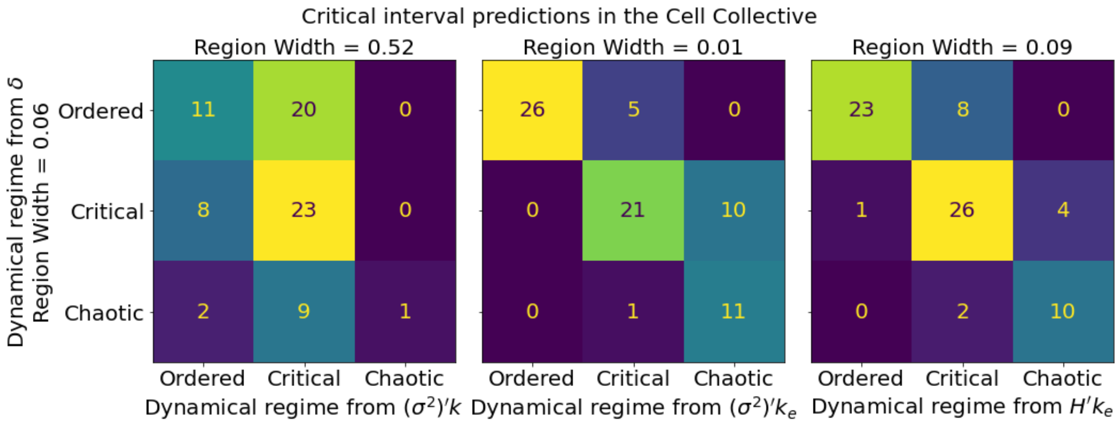

Figure S14: Same as Figure 8.

[ ]:
```
fs = 36
fsa = 24
fig, ax = plt.subplots(1,3,figsize=(30, 10),facecolor='white',sharey='row')
cmap = plt.cm.get_cmap('coolwarm')

dd=np.subtract(*np.percentile(dfcc['dc'], [75, 25]))/2
sc=ax[0].scatter(dfcc['avgV']*dfcc['k'],dfcc['dc'],s=100,c=dfcc['s'],cmap=cmap,
              norm=CenteredNorm(vcenter=1,halfrange=0.5),alpha=1.0)
ax[0].set_ylim(0.5,1.5)
ax[0].set_xlim(0,1.5)
ax[0].set_xlabel('$(\sigma^2)^\prime k$',fontsize=fs)
ax[0].set_ylabel('$\delta$',fontsize=fs)
ax[0].tick_params(axis='both', which='major', labelsize=fsa)
ax[0].hlines([1-dd,1+dd],xmin=0,xmax=1.5,colors='k',linestyles='--')
ax[0].hlines([1],xmin=0,xmax=2,colors='k',linestyles='-')
ax[0].vlines([acut_KV-cr_KV,acut_KV+cr_KV],ymin=0,ymax=1.
↪5,colors='k',linestyles='--')
ax[0].vlines([acut_KV],ymin=0,ymax=1.5,colors='k',linestyles='-')

sc=ax[1].scatter(dfcc['avgV']*dfcc['ke'],dfcc['dc'],s=100,c=dfcc['s'],cmap=cmap,
```

```
              norm=CenteredNorm(vcenter=1,halfrange=0.5),alpha=1.0)
ax[1].set_ylim(0.5,1.5)
ax[1].set_xlim(0,0.5)
ax[1].set_xlabel('$(\sigma^2)^\prime k_e$',fontsize=fs)
ax[1].tick_params(axis='both', which='major', labelsize=fsa)
ax[1].hlines([1-dd,1+dd],xmin=0,xmax=0.5,colors='k',linestyles='--')
ax[1].hlines([1],xmin=0,xmax=2,colors='k',linestyles='-')
ax[1].vlines([acut_KeV-cr_KeV,acut_KeV+cr_KeV],ymin=0,ymax=1.
 ↪5,colors='k',linestyles='--')
ax[1].vlines([acut_KeV],ymin=0,ymax=1.5,colors='k',linestyles='-')

sc=ax[2].scatter(dfcc['avgH']*dfcc['ke'],dfcc['dc'],s=100,c=dfcc['s'],cmap=cmap,
              norm=CenteredNorm(vcenter=1,halfrange=0.5),alpha=1.0)
ax[2].set_ylim(0.5,1.5)
ax[2].set_xlim(0,2)
ax[2].set_xlabel('$H^\prime k_e$',fontsize=fs)
ax[2].tick_params(axis='both', which='major', labelsize=fsa)
ax[2].hlines([1-dd,1+dd],xmin=0,xmax=2,colors='k',linestyles='--')
ax[2].hlines([1],xmin=0,xmax=2,colors='k',linestyles='-')
ax[2].vlines([acut_KeH-cr_KeH,acut_KeH+cr_KeH],ymin=0,ymax=1.
 ↪5,colors='k',linestyles='--')
ax[2].vlines([acut_KeH],ymin=0,ymax=1.5,colors='k',linestyles='-')

cbar=fig.colorbar(sc,ax=ax,location='bottom',aspect=75)
cbar.ax.tick_params(axis='both',labelsize=fsa)
cbar.set_label('Sensitivity',fontsize=fs)

plt.savefig(f'figures/DerridaConnectivitySpread_CC.pdf',bbox_inches='tight')
plt.savefig(f'figures/DerridaConnectivitySpread_CC.png',bbox_inches='tight')
plt.show()
```

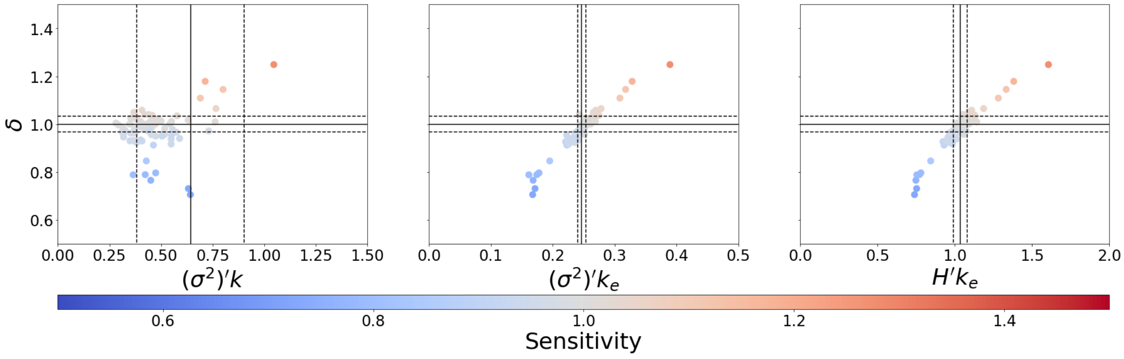

Figure S15: Same as Figure 4.

3.4.5 ROC and PRC Plots

We also explore the effect of varying the threshold for the critical boundary for the various measures. We begin by constructing the ROCs.

[ ]:
```
fs = 24
fig, ax = plt.subplots(1,1, figsize=(
        10, 10), sharey='row', sharex='col', facecolor='white')
truth = (dfcc['dc']>1)

x = dfcc['k']*dfcc['avgV']
fp = np.array([np.sum((x > cut) & ~truth) for cut in sorted(x)])
tp = np.array([np.sum((x > cut) & truth) for cut in sorted(x)])
tn = np.array([np.sum((x <= cut) & ~truth) for cut in sorted(x)])
fn = np.array([np.sum((x <= cut) & truth) for cut in sorted(x)])
fpr = fp/(tn+fp)
tpr = tp/(tp+fn)
AUC=-np.sum(tpr[0:-1]*np.diff(fpr)).round(3)
ax.plot(fpr,tpr,'-.',c='orange',label=f'$(\sigma^2)^\prime k$, {AUC=}')

x = dfcc['ke']*dfcc['avgV']
fp = np.array([np.sum((x > cut) & ~truth) for cut in sorted(x)])
tp = np.array([np.sum((x > cut) & truth) for cut in sorted(x)])
tn = np.array([np.sum((x <= cut) & ~truth) for cut in sorted(x)])
fn = np.array([np.sum((x <= cut) & truth) for cut in sorted(x)])
fpr = fp/(tn+fp)
tpr = tp/(tp+fn)
AUC=-np.sum(tpr[0:-1]*np.diff(fpr)).round(3)
ax.plot(fpr,tpr,'--',c='green',label=f'$(\sigma^2)^\prime k_e$, {AUC=}')

x = dfcc['ke']*dfcc['avgH']
fp = np.array([np.sum((x > cut) & ~truth) for cut in sorted(x)])
tp = np.array([np.sum((x > cut) & truth) for cut in sorted(x)])
tn = np.array([np.sum((x <= cut) & ~truth) for cut in sorted(x)])
fn = np.array([np.sum((x <= cut) & truth) for cut in sorted(x)])
fpr = fp/(tn+fp)
tpr = tp/(tp+fn)
AUC=-np.sum(tpr[0:-1]*np.diff(fpr)).round(3)
ax.plot(fpr,tpr,'-',c='blue',label=f'$H^\prime k_e$, {AUC=}')

ax.set_ylabel('True Positive Rate', fontsize=fs)
ax.set_xlabel('False Positive Rate', fontsize=fs)
ax.legend(fontsize=fs)
ax.tick_params(axis='both', which='major', labelsize=fs)
ax.set_title('ROCs for chaotic regime ($\delta>1$)',fontsize=fs)
plt.savefig(f'figures/ROC_DC.pdf')
plt.savefig(f'figures/ROC_DC.png')
plt.show()
```

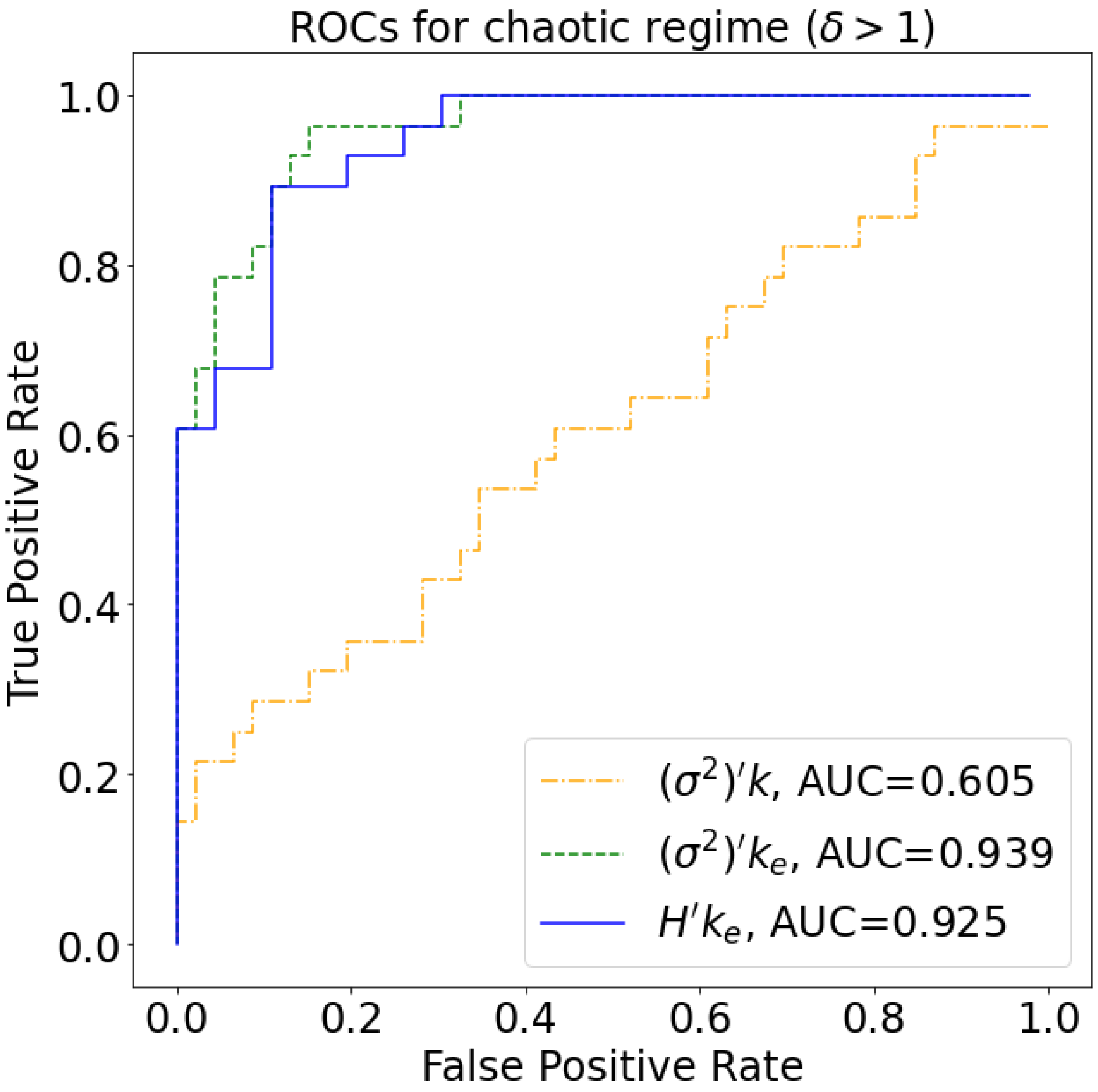

Figure S16: Same as Figure 7 (left).

We now construct the PRCs.

[ ]:
```
fs = 24
fig, ax = plt.subplots(1,1, figsize=(
        10, 10), sharey='row', sharex='col', facecolor='white')
truth = (dfcc['dc']>1)

x = dfcc['k']*dfcc['avgV']
fp = np.array([np.sum((x > cut) & ~truth) for cut in sorted(x)])
tp = np.array([np.sum((x > cut) & truth) for cut in sorted(x)])
tn = np.array([np.sum((x <= cut) & ~truth) for cut in sorted(x)])
fn = np.array([np.sum((x <= cut) & truth) for cut in sorted(x)])
```

```
prec = tp/(tp+fp)
reca = tp/(tp+fn)
AUC=-np.sum(prec[0:-1]*np.diff(reca)).round(3)
ax.plot(reca,prec,'-.',c='orange',label=f'$(\sigma^2)^\prime k$, {AUC=}')

x = dfcc['ke']*dfcc['avgV']
fp = np.array([np.sum((x > cut) & ~truth) for cut in sorted(x)])
tp = np.array([np.sum((x > cut) & truth) for cut in sorted(x)])
tn = np.array([np.sum((x <= cut) & ~truth) for cut in sorted(x)])
fn = np.array([np.sum((x <= cut) & truth) for cut in sorted(x)])
prec = tp/(tp+fp)
reca = tp/(tp+fn)
AUC=-np.nansum(prec[0:-1]*np.diff(reca)).round(3)
ax.plot(reca,prec,'--',c='green',label=f'$(\sigma^2)^\prime k_e$, {AUC=}')

x = dfcc['ke']*dfcc['avgH']
fp = np.array([np.sum((x > cut) & ~truth) for cut in sorted(x)])
tp = np.array([np.sum((x > cut) & truth) for cut in sorted(x)])
tn = np.array([np.sum((x <= cut) & ~truth) for cut in sorted(x)])
fn = np.array([np.sum((x <= cut) & truth) for cut in sorted(x)])
prec = tp/(tp+fp)
reca = tp/(tp+fn)
AUC=-np.nansum(prec[0:-1]*np.diff(reca)).round(3)
ax.plot(reca,prec,'-',c='blue',label=f'$H^\prime k_e$, {AUC=}')

noskill=np.sum(truth)/len(truth)
ax.hlines(noskill,0,1,color='k',linestyle='--',label="no skill classifier")

ax.set_xlim(0,1)
ax.set_ylim(noskill-0.05,1.05)

ax.set_xlabel('Recall', fontsize=fs)
ax.set_ylabel('Precision', fontsize=fs)
ax.legend(fontsize=fs,loc='lower left')
ax.tick_params(axis='both', which='major', labelsize=fs)
ax.set_title('PRCs for chaotic regime ($\delta>1$)',fontsize=fs)
plt.savefig(f'figures/PRC_DC.pdf')
plt.savefig(f'figures/PRC_DC.png')
plt.show()
```

```
/tmp/ipykernel_35093/58602637.py:11: RuntimeWarning: invalid value encountered
in true_divide
  prec = tp/(tp+fp)
/tmp/ipykernel_35093/58602637.py:21: RuntimeWarning: invalid value encountered
in true_divide
  prec = tp/(tp+fp)
/tmp/ipykernel_35093/58602637.py:31: RuntimeWarning: invalid value encountered
```

```
in true_divide
  prec = tp/(tp+fp)
```

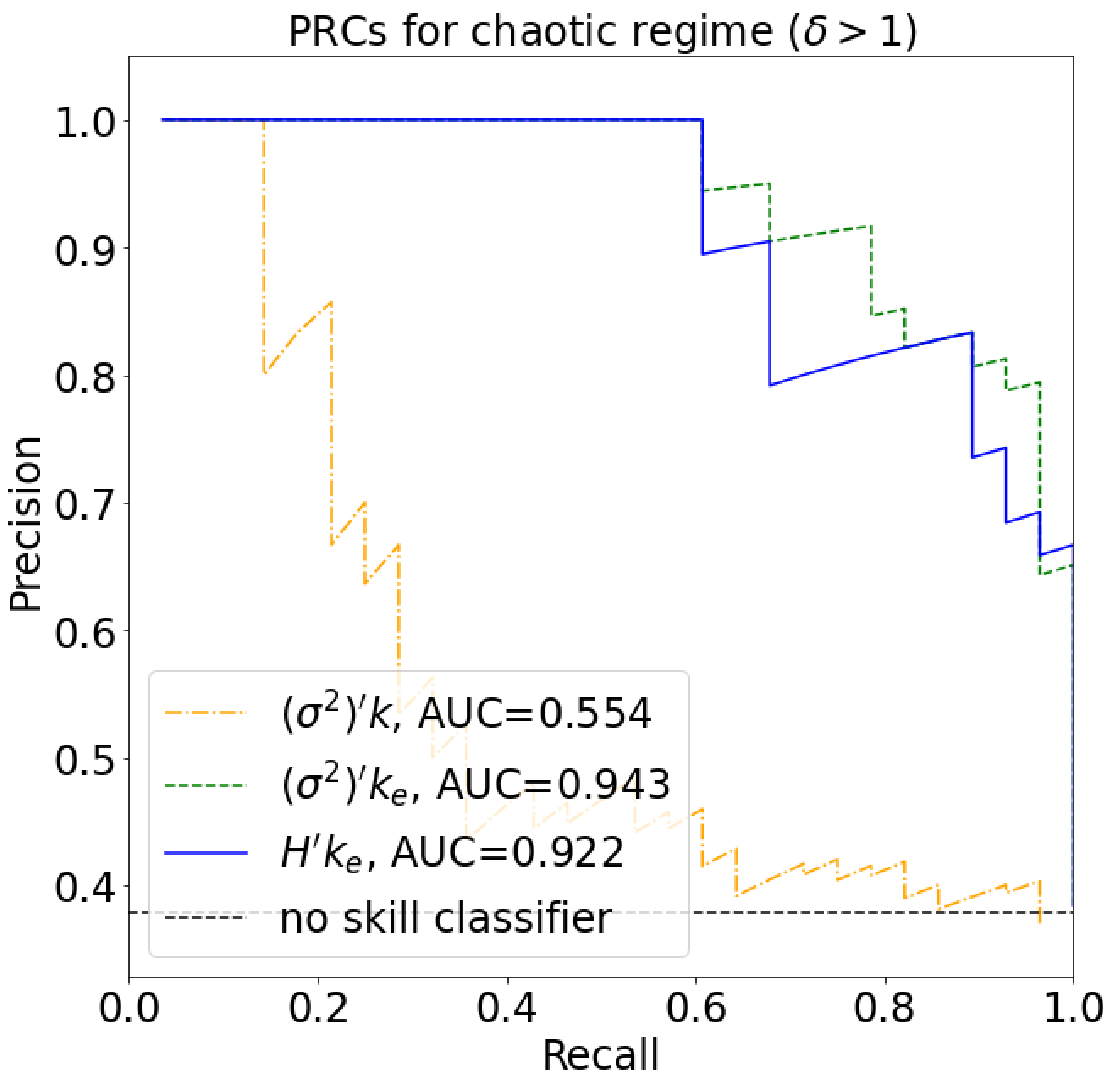

Figure S17: Same as Figure 7 (right).

3.4.6 Prediction of Dynamical Regime Using Sensitivity as Ground Truth

We also consider the ability of these parameters to predict the dynamical regime in terms of sensitivity.

[ ]:
```
fs = 24

truth = (dfcc['s'] > 1)
fig, ax = plt.subplots(3,3, figsize=(
        10, 10), sharey='row', sharex='col', facecolor='white')
```

```
for row,method in enumerate(['MCC','Accuracy','Cohen kappa']):


    x = dfcc['k']*dfcc['avgV']
    preds,cut,score=optimize_cut(x,truth,method=method)
    confusion_matrix = metrics.confusion_matrix(truth, preds)
    cm_display = metrics.ConfusionMatrixDisplay(
        confusion_matrix=confusion_matrix, display_labels=['$s\leq 1$',␣
 ↪'$s>1$'])
    cm_display.plot(ax=ax[row,0])
    cm_display.im_.colorbar.remove()
    label = f'$(\sigma^2)^\prime k$={np.round(cut,3)}, {method}={np.
 ↪round(score,2)}'
    ax[row,0].set_title(label,fontsize=fs/2)

    x = dfcc['ke']*dfcc['avgV']
    preds,cut,score=optimize_cut(x,truth,method=method)
    confusion_matrix = metrics.confusion_matrix(truth, preds)
    cm_display = metrics.ConfusionMatrixDisplay(
        confusion_matrix=confusion_matrix, display_labels=['$s\leq 1$',␣
 ↪'$s>1$'])
    cm_display.plot(ax=ax[row,1])
    cm_display.im_.colorbar.remove()
    label = f'$(\sigma^2)^\prime k_e$={np.round(cut,3)}, {method}={np.
 ↪round(score,2)}'
    ax[row,1].set_title(label,fontsize=fs/2)

    x = dfcc['ke']*dfcc['avgH']
    preds,cut,score=optimize_cut(x,truth,method=method)
    confusion_matrix = metrics.confusion_matrix(truth, preds)
    cm_display = metrics.ConfusionMatrixDisplay(
        confusion_matrix=confusion_matrix, display_labels=['$s\leq 1$',␣
 ↪'$s>1$'])
    cm_display.plot(ax=ax[row,2])
    cm_display.im_.colorbar.remove()
    label = f'$H^\prime k_e$={np.round(cut,3)}, {method}={np.round(score,2)}'
    ax[row,2].set_title(label,fontsize=fs/2)

fig.tight_layout()
plt.savefig(f'figures/SensitivityConfusionOptimized_CC.pdf',bbox_inches='tight')
plt.savefig(f'figures/SensitivityConfusionOptimized_CC.png',bbox_inches='tight')
plt.show()
```

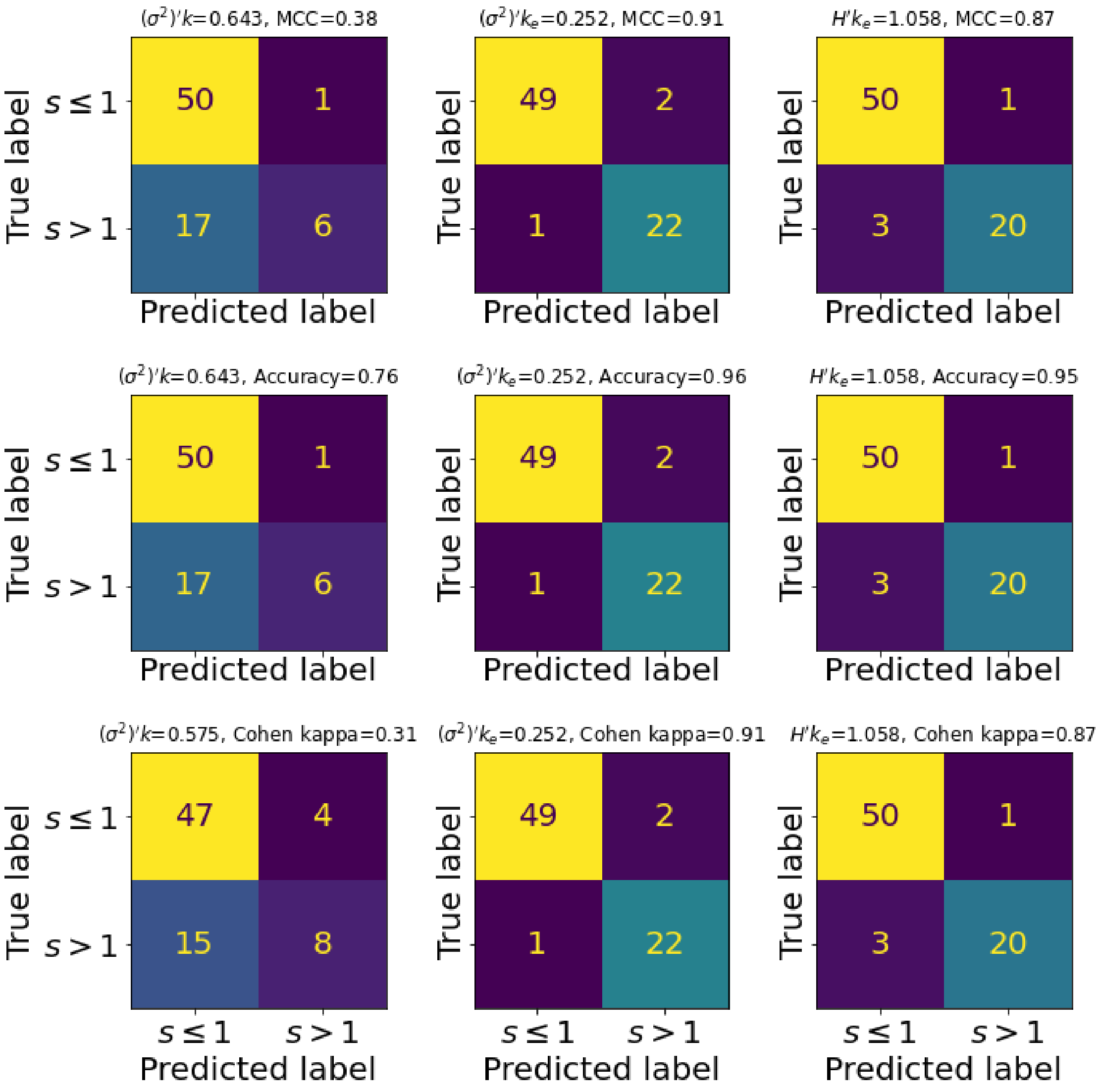

Figure S18: Confussion matrix compared to sensitivity instead of Derrida coefficient.

Notably, $(\sigma^2)'k_e$ and $H'k_e$ show very good agreement with the sensitivity.

We now build the ROCs for sensitivity prediction.

[ ]:
```
fs = 24
fig, ax = plt.subplots(1,1, figsize=(
        10, 10), sharey='row', sharex='col', facecolor='white')
truth = (dfcc['s']>1)

x = dfcc['k']*dfcc['avgV']
fp = np.array([np.sum((x > cut) & ~truth) for cut in sorted(x)])
tp = np.array([np.sum((x > cut) & truth) for cut in sorted(x)])
```

```
tn = np.array([np.sum((x <= cut) & ~truth) for cut in sorted(x)])
fn = np.array([np.sum((x <= cut) & truth) for cut in sorted(x)])
fpr = fp/(tn+fp)
tpr = tp/(tp+fn)
AUC=-np.sum(tpr[0:-1]*np.diff(fpr)).round(3)
ax.plot(fpr,tpr,'-.',c='orange',label=f'$(\sigma^2)^\prime k$, {AUC=}')

x = dfcc['ke']*dfcc['avgV']
fp = np.array([np.sum((x > cut) & ~truth) for cut in sorted(x)])
tp = np.array([np.sum((x > cut) & truth) for cut in sorted(x)])
tn = np.array([np.sum((x <= cut) & ~truth) for cut in sorted(x)])
fn = np.array([np.sum((x <= cut) & truth) for cut in sorted(x)])
fpr = fp/(tn+fp)
tpr = tp/(tp+fn)
AUC=-np.sum(tpr[0:-1]*np.diff(fpr)).round(3)
ax.plot(fpr,tpr,'--',c='green',label=f'$(\sigma^2)^\prime k_e$, {AUC=}')

x = dfcc['ke']*dfcc['avgH']
fp = np.array([np.sum((x > cut) & ~truth) for cut in sorted(x)])
tp = np.array([np.sum((x > cut) & truth) for cut in sorted(x)])
tn = np.array([np.sum((x <= cut) & ~truth) for cut in sorted(x)])
fn = np.array([np.sum((x <= cut) & truth) for cut in sorted(x)])
fpr = fp/(tn+fp)
tpr = tp/(tp+fn)
AUC=-np.sum(tpr[0:-1]*np.diff(fpr)).round(3)
ax.plot(fpr,tpr,'-',c='blue',label=f'$H^\prime k_e$, {AUC=}')

ax.set_ylabel('True Positive Rate', fontsize=fs)
ax.set_xlabel('False Positive Rate', fontsize=fs)
ax.legend(fontsize=fs)
ax.tick_params(axis='both', which='major', labelsize=fs)
ax.set_title('ROCs for chaotic regime ($s>1$)',fontsize=fs)
plt.savefig(f'figures/ROC_S.pdf')
plt.savefig(f'figures/ROC_S.png')
plt.show()
```

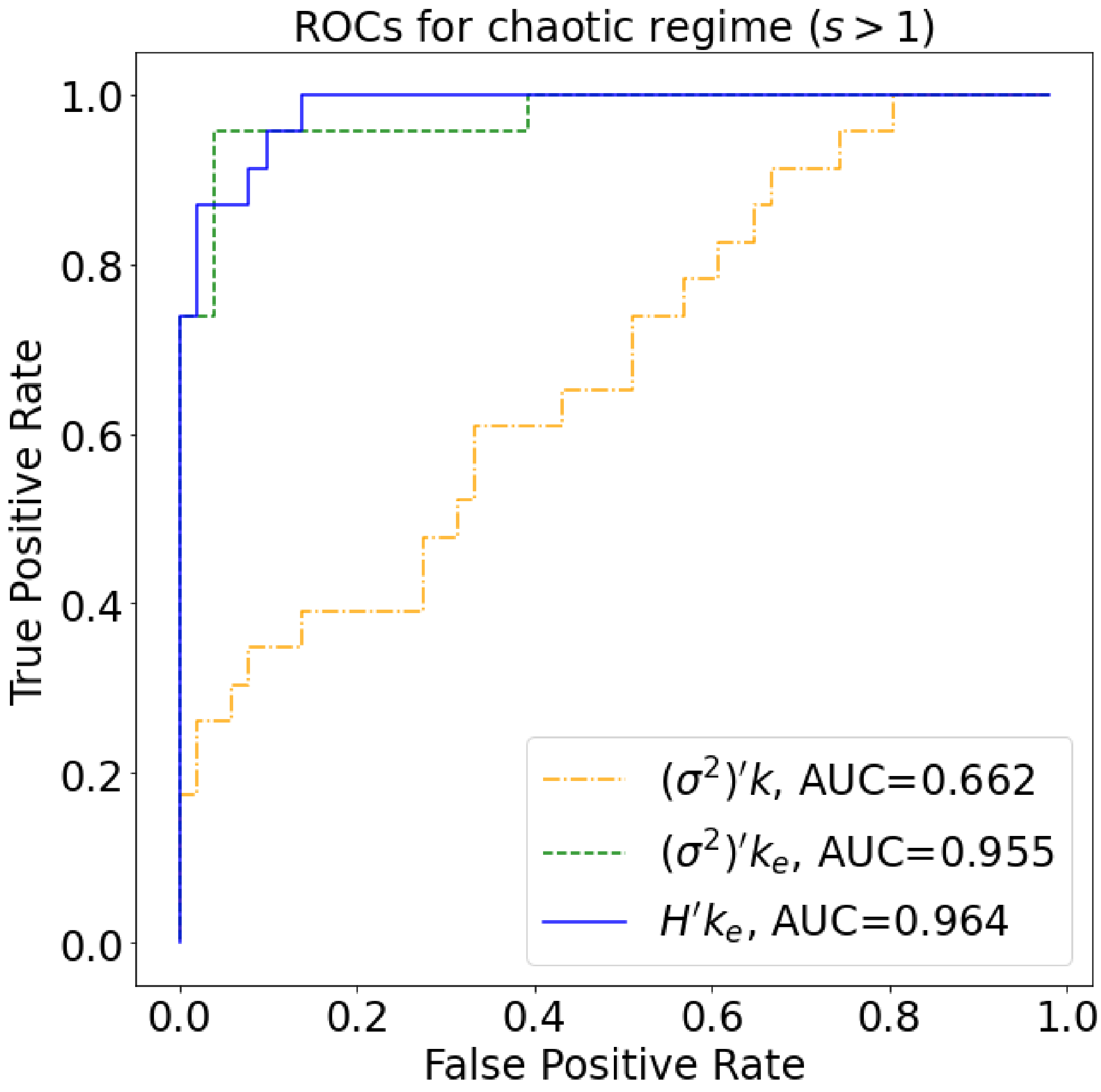

Figure S19: ROCs compared to sensitivity instead of Derrida coefficient.

We now build the PRCs for sensitivity prediction.

[ ]:
```
fs = 24
fig, ax = plt.subplots(1,1, figsize=(
        10, 10), sharey='row', sharex='col', facecolor='white')
truth = (dfcc['s']>1)

x = dfcc['k']*dfcc['avgV']
fp = np.array([np.sum((x > cut) & ~truth) for cut in sorted(x)])
tp = np.array([np.sum((x > cut) & truth) for cut in sorted(x)])
tn = np.array([np.sum((x <= cut) & ~truth) for cut in sorted(x)])
fn = np.array([np.sum((x <= cut) & truth) for cut in sorted(x)])
```

```
prec = tp/(tp+fp)
reca = tp/(tp+fn)
AUC=-np.sum(prec[0:-1]*np.diff(reca)).round(3)
ax.plot(reca,prec,'-.',c='orange',label=f'$(\sigma^2)^\prime k$, {AUC=}')

x = dfcc['ke']*dfcc['avgV']
fp = np.array([np.sum((x > cut) & ~truth) for cut in sorted(x)])
tp = np.array([np.sum((x > cut) & truth) for cut in sorted(x)])
tn = np.array([np.sum((x <= cut) & ~truth) for cut in sorted(x)])
fn = np.array([np.sum((x <= cut) & truth) for cut in sorted(x)])
prec = tp/(tp+fp)
reca = tp/(tp+fn)
AUC=-np.nansum(prec[0:-1]*np.diff(reca)).round(3)
ax.plot(reca,prec,'--',c='green',label=f'$(\sigma^2)^\prime k_e$, {AUC=}')

x = dfcc['ke']*dfcc['avgH']
fp = np.array([np.sum((x > cut) & ~truth) for cut in sorted(x)])
tp = np.array([np.sum((x > cut) & truth) for cut in sorted(x)])
tn = np.array([np.sum((x <= cut) & ~truth) for cut in sorted(x)])
fn = np.array([np.sum((x <= cut) & truth) for cut in sorted(x)])
prec = tp/(tp+fp)
reca = tp/(tp+fn)
AUC=-np.nansum(prec[0:-1]*np.diff(reca)).round(3)
ax.plot(reca,prec,'-',c='blue',label=f'$H^\prime k_e$, {AUC=}')

noskill=np.sum(truth)/len(truth)
ax.hlines(noskill,0,1,color='k',linestyle='--',label="no skill classifier")

ax.set_xlim(0,1)
ax.set_ylim(noskill-0.05,1.05)

ax.set_xlabel('Recall', fontsize=fs)
ax.set_ylabel('Precision', fontsize=fs)
ax.legend(fontsize=fs,loc='lower left')
ax.tick_params(axis='both', which='major', labelsize=fs)
ax.set_title('PRCs for chaotic regime ($s>1$)',fontsize=fs)
plt.savefig(f'figures/PRC_S.pdf')
plt.savefig(f'figures/PRC_S.png')
plt.show()
```

```
/tmp/ipykernel_35093/3588301391.py:11: RuntimeWarning: invalid value encountered
in true_divide
  prec = tp/(tp+fp)
/tmp/ipykernel_35093/3588301391.py:21: RuntimeWarning: invalid value encountered
in true_divide
  prec = tp/(tp+fp)
/tmp/ipykernel_35093/3588301391.py:31: RuntimeWarning: invalid value encountered
```

```
in true_divide
  prec = tp/(tp+fp)
```

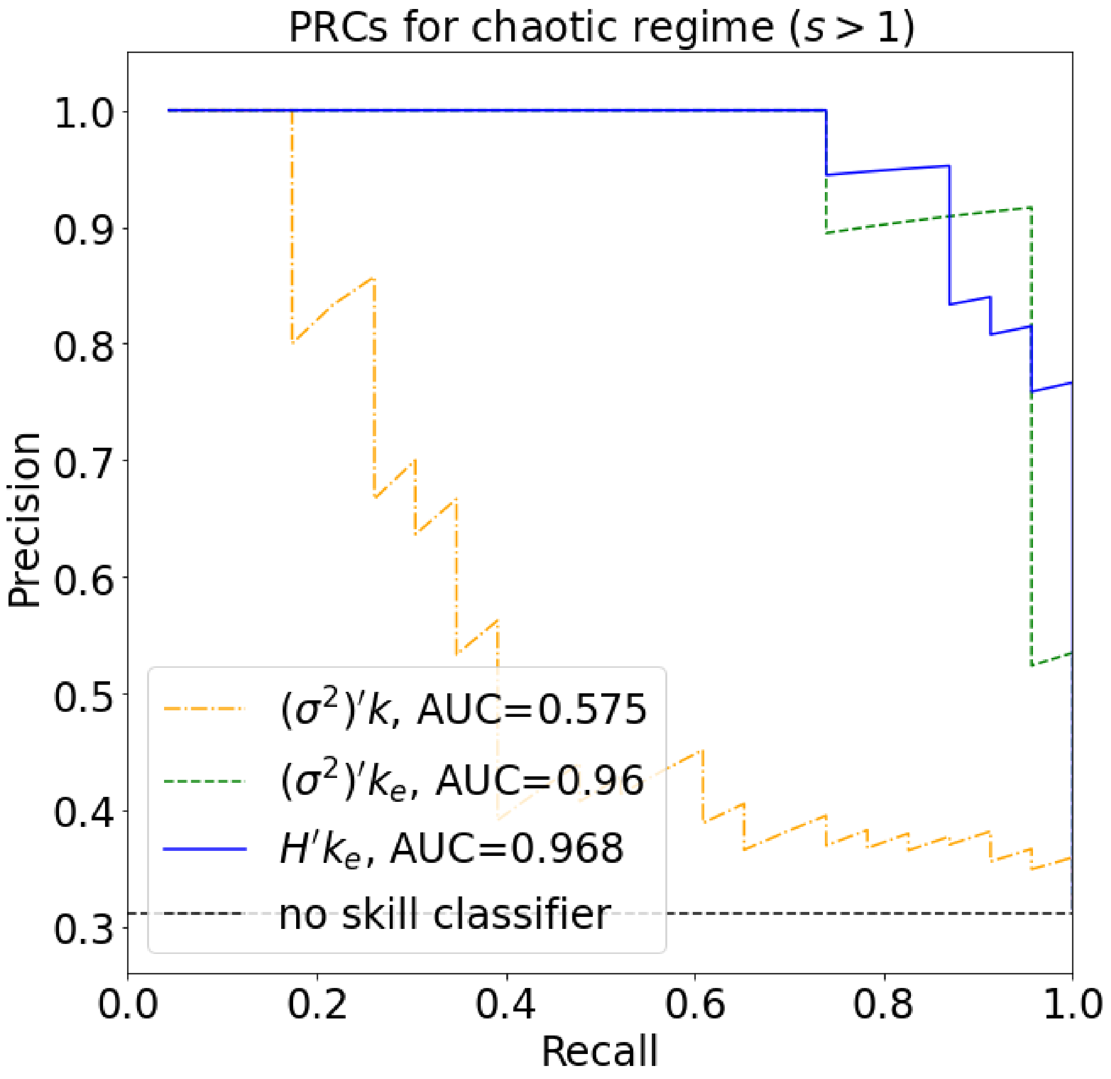

Figure S20: PRCs compared to sensitivity instead of Derrida coefficient.

```
[ ]:
```
